# Improved motor imagery BCI performance via task-unaware compression in the BELT Bayesian Edge-Cloud architecture

**DOI:** 10.1371/journal.pone.0354976

**Published:** 2026-08-21

**Authors:** Abolfazl Danayi, Hamid Soltanian-Zadeh

**Affiliations:** 1 School of Electrical and Computer Engineering, College of Engineering, University of Tehran, Tehran, Iran; 2 School of Cognitive Sciences, Institute for Research in Fundamental Sciences (IPM), Tehran, Iran; 3 Departments of Research Administration and Radiology, Henry Ford Health System, Detroit, Michigan, United States of America; Karadeniz Technical University: Karadeniz Teknik Universitesi, TÜRKIYE

## Abstract

Brain–computer interface (BCI) systems have advanced with deep learning, but they are still limited by designs tied to specific applications, poor scalability, weak portability, the need for user-specific adaptation, and privacy concerns. We present **BELT**, a modular Bayesian Edge–Cloud architecture based on three principles: (i) Bayesian priors and posteriors to balance generalization and subject-specific learning, (ii) lightweight classifiers suitable for embedded devices, and (iii) task-aware compression to reduce bandwidth and improve privacy in edge–cloud communication. To show feasibility, we implement **BELT-lite** as an instantiation of BELT, a lightweight version built only from linear time-invariant operations, making it directly compatible with digital signal processing hardware. Using the BCI Competition IV-2a and IV-2b motor imagery datasets (18 subjects total, ten-fold cross-validation), BELT-lite achieved strong posterior performance after subject-specific fine-tuning: mean accuracy of 87.9%±6.8% on Dataset B and 80.6%±8.6% on Dataset A with data augmentation. After adaptation, four subjects from Dataset B and two from Dataset A exceeded 90% accuracy. On ARM Cortex-A7 hardware, BELT-lite achieved a mean latency of 6.75 ms per sample, significantly faster than EEGNet’s 8.36 ms (*p* < 10^-17^)—a 21% speed improvement—at the cost of a modest but statistically significant accuracy reduction of approximately 2.7 percentage points compared to EEGNet. Network Tuning Blocks allowed partial parameter freezing: classifier-only fine-tuning incurred a modest 2–5% accuracy drop while substantially reducing training cost. Compression via the task-unaware autoencoder reduced data size by 3.3× while maintaining high accuracy: prior-model performance stayed within ≈1% of the uncompressed baseline (with slight improvements in some configurations), full posterior fine-tuning showed a ≈1% drop, and classifier-only fine-tuning incurred a ≈3% drop—an acceptable trade-off for privacy-preserving edge–cloud communication, where only a compressed latent representation is transmitted instead of raw EEG. Notably, this task-unaware autoencoder (trained solely to reconstruct the input) consistently outperformed autoencoders that also incorporated classification objectives (task-aware or task-only), providing the best accuracy–compression trade-off across all fine-tuning scenarios. These findings show that BELT provides a principled design for modular and scalable BCIs, while BELT-lite demonstrates that the approach supports accurate, efficient, and portable implementations. Together, they point toward BCI systems that are more practical, mass-producible, and privacy-aware, enabling wider use in real-world settings.

## Introduction

Since Hans Berger’s first scalp EEG recordings in the 1920s and Jacques J. Vidal’s coining of the term *Brain-Computer Interface* (BCI) in 1973 [1,2], the ambition to directly link the human brain to external devices has driven decades of research. Today, affordable consumer-grade EEG headsets such as the Emotiv EPOC are widely used in BCI studies [3,4], yet BCIs have not approached the ubiquity of conventional human–computer interaction (HCI) devices like the mouse or keyboard. Persistent challenges to BCI development have been reported, including biological variability, ethical and privacy concerns, and constraints of real-time portable systems [5–7]. However, limited integration of BCIs into everyday human–computer interaction cannot be attributed solely to deficiencies in processing power or hardware: as early as 1991, Wolpaw et al. demonstrated reliable cursor control using spectral analysis on hardware with minimal computational resources [8], while modern portable systems are orders of magnitude more capable. Deeper architectural and practical obstacles remain.

An underexplored challenge in BCI research is the tight coupling between BCI systems as information-transfer media and their specific end applications. In many studies, task-specific goals dominate system design to the extent that general communication components—potentially reusable across applications—are subordinated to application logic, leading to ad-hoc solutions [9,10]. Such designs hinder generalization, reuse, and broader deployment. Modular design strategies have been proposed to address this issue [11]; notably, Wolpaw advocated separating goal selection from process control to enhance architectural modularity in BCIs [12]. Adopting this approach, we propose a general-purpose BCI architecture that emphasizes modularity beyond signal processing while accommodating task-specific requirements through a dedicated Network Tuning Block (NTB). The NTB provides a structured interface for incorporating neurological and physiological task-specific findings, enabling transparent intention decoding at the architectural level [13].

Another key challenge in BCI systems is the pronounced inter- and intra-subject variability of neural signals, which necessitates user adaptation and persists even across sessions in non-invasive EEG [5,14]. Deep learning (DL) approaches have shown strong potential in addressing both inter-subject and within-subject day-to-day variability and are increasingly adopted in BCI research [15–18]. Our architecture therefore explicitly accommodates DL methods. However, the use of DL increases the computational demands on end devices, which are not only limited in processing power but are also preferred to be simple, low-cost, and energy-efficient. A promising approach to maintaining affordable end devices while preserving decoding performance is the use of cloud computing, where on-demand processing resources are accessed over the internet. However, cloud-based solutions introduce several challenges, including data transfer overhead, latency, privacy concerns, and reliance on stable internet connectivity [19–21]. Moreover, offloading all processing tasks to the cloud places additional demands on cloud-specific mechanisms such as task allocation and automatic scheduling [22]. Therefore, Edge–Cloud architectures present a more viable alternative to purely cloud-based BCI systems by introducing an intermediate tier closer to the user. This edge tier can perform selected processing tasks to reduce cloud load while also improving latency and privacy.

These observations lead to three core design rules for a practical BCI architecture:

1. **Bayesian Setup:** The system should operate effectively for a general user (prior) while also supporting subject-specific fine-tuning (posterior) to boost accuracy.
2. **Lightweight Models:** Classification models must run efficiently on mass-produced embedded microprocessors.
3. **Efficient Compression:** Data compression is essential to optimise edge-to-cloud communication in terms of bandwidth and latency, while also providing a degree of data privacy.

Together, these rules define the *Bayesian Edge-Cloud architecture with Lightweight classification and Task-aware compression (BELT)*. We also present **BELT-lite**, a lightweight deep learning model designed for Motor Imagery (MI) tasks within the BELT framework, optimised for embedded deployment.

### Main contributions

- We introduce the BELT architecture, a modular BCI design paradigm that separates general-purpose communication decoding from application-specific logic, enabling broader reusability.
- We propose a Bayesian two-phase decoding strategy that combines a general prior model with user-specific posterior adaptation, improving accuracy while reducing the need for extensive individual training.
- We emphasise practical deployment by enforcing strict design rules: lightweight classification for embedded hardware, task-aware data compression, and an Edge-Cloud infrastructure to balance computation, bandwidth, and privacy.
- We present BELT-lite, a deep learning-based model for MI tasks within the BELT framework, tailored for resource-constrained environments and demonstrating the feasibility of our approach in real-world settings.

### Related work and current challenges

The challenges facing portable BCI systems are well documented and directly motivate the design choices in BELT. We organise our review around four key areas: signal variability and adaptation, embedded deployment constraints, edge–cloud communication, and data privacy. For each area we briefly survey relevant literature and indicate how BELT addresses the identified challenge—fully or partially.

#### Variability and deep learning adaptation.

EEG signals are inherently non-stationary, with pronounced inter- and intra-subject variability that persists across sessions [5,14,23]. Deep learning (DL) has proved effective at learning such variability, and transfer learning—pre-training on large multi-subject datasets followed by fine-tuning on a small amount of target-subject data—significantly improves accuracy and reduces calibration time [24–27]. Multi-task training can further enhance generalisation [28], although it introduces additional training complexity. The BELT architecture directly addresses this challenge through its Bayesian two-phase design: a prior model is pre-trained on population data (the equivalent of the pre-training stage), while posterior fine-tuning on the target user performs the adaptation step. Our experiments confirm that this approach yields substantial accuracy gains, fully realising the benefits demonstrated in the transfer-learning literature.

#### Embedded deployment and portable hardware.

Developing portable BCI systems still presents several reported challenges such as the high cost of sensors, reimplementation difficulties due to hardware differences, reduced number of effective sensors, lower sampling rates, and narrower effective frequency ranges compared to non-portable BCI devices [29]. These factors make it challenging to run complex DL models directly on the device, and they also affect the quality of the recorded EEG. BELT-lite is designed specifically for this regime: with only 1.65 k parameters and exclusively FIR-based operations, it runs efficiently on embedded ARM processors while maintaining competitive accuracy. The latency and parameter-count benchmarks reported in this paper demonstrate that lightweight, mass-producible BCI decoders are feasible. Sensor cost and hardware miniaturisation, however, remain beyond the scope of the present architectural contribution (partially solved).

#### Edge–Cloud architectures and connectivity.

Offloading model adaptation to the cloud reduces the computational burden on the portable device but introduces latency, bandwidth, and connectivity dependencies [30]. Two-tier architectures, in which the portable device communicates directly with a cloud server, require continuous internet access, which is unrealistic in many everyday BCI scenarios. Three-tier architectures insert an intermediate edge server, improving robustness and real-time performance [31]. BELT explicitly adopts a three-tier Edge-Cloud model: the lightweight BELT-lite model runs on the edge device, while more intensive processing—including fine-tuning—can be offloaded to a nearby edge server or the cloud. Moreover, because BELT-lite is capable of performing inference entirely locally, the system can continue to operate during connectivity interruptions, a feature that directly mitigates the risk of “always-online” dependence. Seamless fallback between cloud and local modes, however, is not fully automated in our current implementation (partially solved).

#### Privacy and data exposure.

BCI technologies collect highly sensitive neural data—such as emotional states, mental intentions, and identity cues—which, if intercepted or misused, pose serious privacy and security concerns. Data transmission channels and cloud/edge storage are potential targets for eavesdropping, man-in-the-middle attacks, and insider threats, putting neural confidentiality, integrity, and availability at risk [9,32]. Minimizing data transfer through compression reduces exposure by limiting the amount of neural data communicated across insecure channels, reducing the attack surface and helping to preserve user privacy and data sovereignty [30,33]. BELT’s task-aware compression is designed precisely for this purpose: it reduces the bandwidth needed for cloud communication while transmitting only a compressed latent representation, not the raw EEG. Although this does not constitute a complete end-to-end security protocol, it provides a meaningful privacy-by-design measure that significantly limits the exposure of neural information (partially solved).

#### Remaining challenges.

Several issues that have been highlighted in the literature are only indirectly addressed by BELT. These include the management of large-scale user data in the cloud [34,35], the integration of multi-task training schemes [28], and the broader commercialisation hurdles identified in systematic reviews [5,36]. While BELT’s modular design and Edge-Cloud infrastructure provide a foundation upon which such extensions could be built, these topics remain open directions for future work.

## Materials and methods

### Architecure

**Fig 1:**
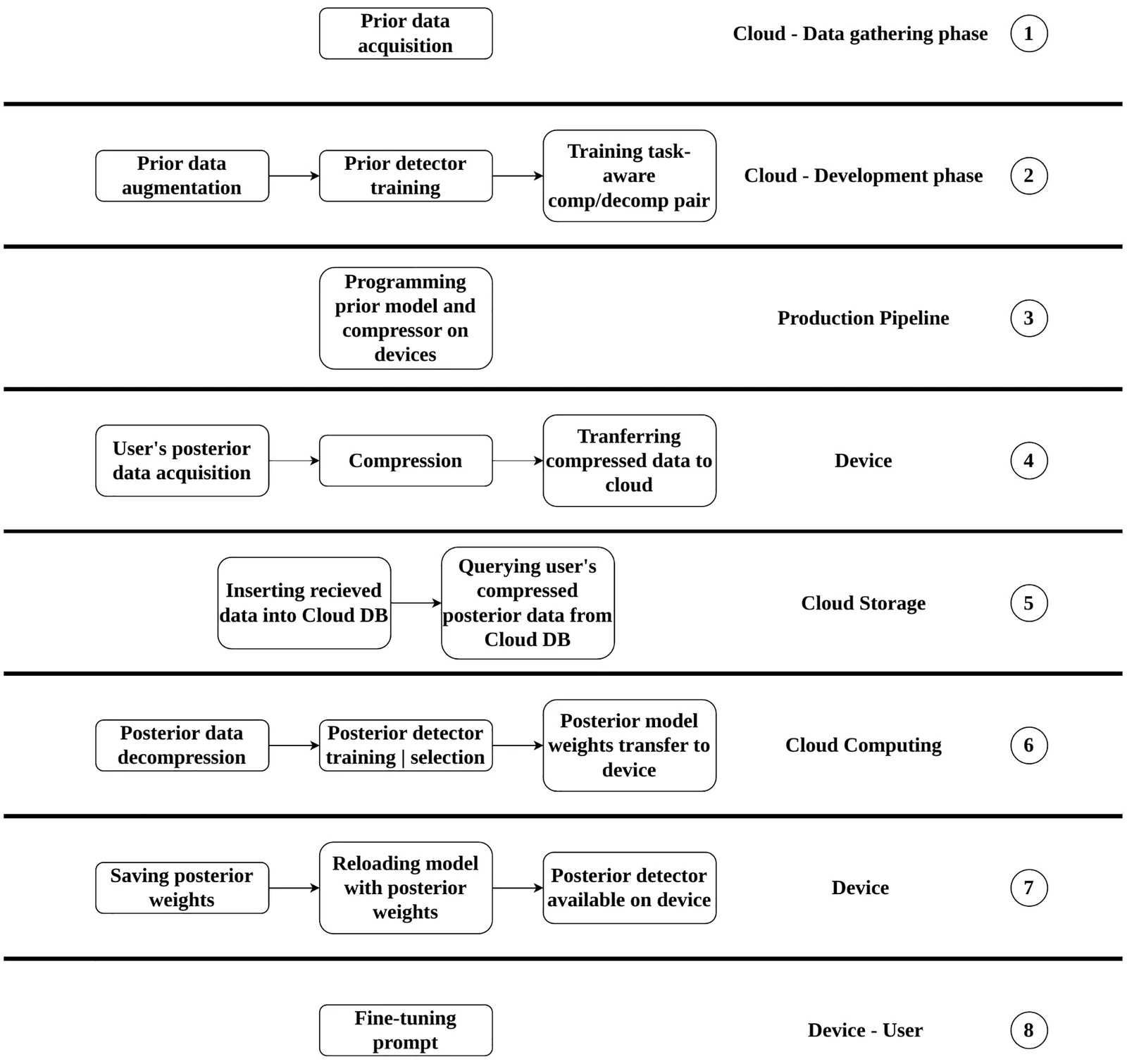
The BELT architecture pipeline. The framework consists of eight steps organized into three phases: (1) Prior phase (Steps 1-3): Prior data collection, prior model training, and compression–decompression pair training. These components are embedded in the device during production. (2) Adaptation phase (Steps 4-7): New EEG data are compressed and transmitted to the cloud (Step 4), stored and combined with existing user history (Step 5), used for posterior model training (Step 6), and the personalized posterior model is transferred back to the device (Step 7). (3) Update phase (Step 8): Optional continuous updates with explicit user consent, balancing personalization with privacy and scalability. Mathematical definitions for each step are provided in the accompanying text.

The proposed pipeline is illustrated in Fig 1 and consists of eight steps. In this section of the paper, the description and mathematical definition of steps are given. All symbols used in the equations throughout this section are summarized in Table 1, along with their corresponding types.

**Table 1:** Mathematical notation used in the BELT architecture.

Symbol	Description	Type
Pr(A|B)	Probability of event *A* given event *B*	Notation
Superscripted →	Vector or Multichannel signal	Notation
$\vec{x}$	EEG signal	Signal
*y*	User intention – target message	Symbol
$\hat{y}$	Estimated user intention – target message	Symbol
$\vec{c}$	Compressed EEG signal	Signal
${\vec{x}}_{r}$	Decompressed EEG signal	Signal
$EEG_{pr}$	Prior data	Set of signals
$EEG_{pr}^{\prime }$	Augmented prior dat	Set of signals
$D(\vec{x})$	Prior detector	System
$Comp(\vec{x})$	Compressor model	System
$Decomp(\vec{c})$	Decompressor model	System
$S_{i}$	*i*th subject data	Set of signals
$D(\vec{x}|S_{i})$	Sub-Optimal Posterior detector	System
$D^{⋆}(\vec{x}|S_{i})$	Optimal Posterior detector	System

Summary of mathematical symbols used throughout the architecture description. The table includes each symbol, its description, and its type classification (Signal, Symbol, Set, System, or Notation). Probability notation *Pr*(*A*|*B*) denotes the probability of event *A* given event *B*, and the arrow superscript (e.g., $\vec{x}$) indicates a vector or multichannel signal.

The first step involves collecting prior data. This is a critical stage, and we emphasize its importance as fundamental to the realization of BCI technologies. Therefore, we dedicate a distinct step exclusively to this process. We denote EEG signals as $\vec{x}$ and the user’s target intention—referred to as the *symbol* or *message* in telecommunication terminology—as *y*. The complete set of prior data is represented by $EEG_{pr}$ and is defined in (1) where *N* is the total number of signal segments in prior dataset.


$$\begin{array}{c} EEG_{pr}=\{({\vec{x}}_{1},y_{1}),({\vec{x}}_{2},y_{2}),...,({\vec{x}}_{N},y_{N})\} \end{array}$$ (1)

With the prior data collected, the next step is to construct the prior model. However, before model training, we emphasize the importance of prior data augmentation, as it has been shown to yield a non-negligible improvement in final classification accuracy. For example, Fahimi et al. used Deep Convolutional Generative Adversarial Networks (DC-GANs) to improve classification accuracy by 3.57% in motor imagery (MI) tasks [37]. Similarly, Al-Saegh et al. applied CutCat [38] —a technique that cuts and concatenates EEG signals from inter- and intra-subject trials—and demonstrated improved performance with smaller datasets. Another study adapted SpecAugment, a data augmentation technique originally developed for speech recognition, to steady-state visually evoked potentials (SSVEP) and reported improved classification performance [39]. Therefore, we incorporate data augmentation into the framework and denote the augmented dataset as $EEG_{pr}^{\prime }$.

Using the augmented data, we train the prior model. This model receives EEG input $\vec{x}$ and estimates the corresponding target symbol $\hat{y}$. Following telecommunication terminology, we refer to this model as the *detector*, denoted by *D*, as defined in (2):


$$\begin{array}{c} \hat{y}=D(\vec{x}) \end{array}$$ (2)

Accordingly, the optimization objective to find the optimal prior model is expressed in (3). This is a probabilistic minimization problem, where θ denotes the parameter space of the detector *D*. The term $EEG_{pr}^{\prime }$ as condition implies that $(\vec{x},y)\in EEG_{pr}^{\prime }$, and this abstraction is used for readability and brevity.


$$\begin{array}{c} D(\vec{x})=\text{arg}\min_{\theta }\;\mathrm{Pr}\left(D(\vec{x})\text{≠}y|EEG_{pr}^{\prime }\right) \end{array}$$ (3)

The next step is to find the compression pair denoted by (*Comp*, *Decomp*). *Comp* will be applied over EEG data $\vec{x}$ and gives us the compressed data $\vec{c}$ as shown in (4) and later by applying the decompressor on $\vec{c}$, we get the decompressed signal ${\vec{x}}_{r}$ depicted in (5). However, for ${\vec{x}}_{r}$, we will prefer to use the reconstructed term instead of decompressed, for better mathematical formulation.


$$\begin{array}{c} \vec{c}=Comp(\vec{x}) \end{array}$$ (4)

$$\begin{array}{c} {\vec{x}}_{r}=Decomp(\vec{c}) \end{array}$$ (5)

The detector plays a crucial role in the development of the task-aware compression and decompression pair, as it is required for computing the task-aware penalty. The overall optimization objective is shown in (6). Solving this optimization problem gives us *Comp* and *Decomp* by simultaneously minimizing $L_{r}$ and $L_{t}$ which represent the data reconstruction loss and the task-specific compression penalty, respectively. The scalar *j* serves as a trade-off parameter between reconstruction fidelity and task relevance. $\theta_{c}$ and $\theta_{d}$ are compressor and decompressor model parameters to be found.

We expect $L_{t}$ to encourage the compressor to act as a form of task-oriented preprocessing. We expect the optimization to result in a compression pair that preserves task-relevant information (via $L_{t}$) while reconstructing a signal as close as possible to the original in the mean squared error (MSE) sense (via $L_{r}$).


$$\begin{array}{c} \left\{Comp(\vec{x}),Decomp(\vec{c})\}=argmin_{\left(\theta_{c},\theta_{d}\right)}\;L_{r}(\theta_{c},\theta_{d})+jL_{t}(\theta_{c},\theta_{d}\right) \end{array}$$ (6)

We define the reconstruction loss $L_{r}$ as the mean squared error (where *E* denotes the expectation operator), given in (7), and the compression task penalty as the detector’s error when given the reconstructed EEG signal, shown in (8).


$$\begin{array}{c} L_{r}(\theta_{c},\theta_{d})=E\left[|\vec{x}r-\vec{x}{|}^{2}|EEG^{\prime }pr\right] \end{array}$$ (7)

$$\begin{array}{c} L_{t}(\theta_{c},\theta_{d})=\mathrm{Pr}\left(D(\vec{x}r)\neq y|EEG^{\prime }pr\right) \end{array}$$ (8)

In the third step, once the prior model and compression–decompression pair are trained, the device can be programmed accordingly in the production pipeline. Steps 4–7 constitute the user adaptation phase.

In Step 4, the user’s EEG signal is acquired, compressed, and transmitted to the cloud via the internet. In Step 5, the cloud first stores the received data in a dedicated cloud storage space. However, performing posterior training on this newly received data alone may be insufficient and result in overfit due to its limited size. Therefore, the second operation in this step is to query all previously stored data belonging to the same user from the cloud storage database (DB).

It is important to note that all stored data is maintained in compressed format. This design reduces storage costs and enhances data privacy, as the decompressor—required to reconstruct the raw EEG signal—is not deployed on user devices. Additionally, the task-aware penalty $L_{t}$ is expected to discard redundant and user-agnostic features, thereby improving privacy and promoting a more ethical data-handling approach.

Step 6 involves posterior training, which is performed on the cloud. First, the compressed posterior data is decompressed into reconstructed signals ${\vec{x}}_{r}$. Then, the posterior model is trained according to the objective in (9). The first term on the right-hand side represents the direct posterior training loss, where the condition $S_{i}$ is a shorthand notation for ${\vec{x}}_{r}\in \text{User[i] posterior data}$. However, training exclusively on this limited posterior data may lead to overfitting, potentially resulting in degraded performance—even for that specific user.

To address this, a second regularization term is introduced. This term acts as a constraint by referencing the prior dataset, preventing the model from deviating too far from generalizable features. The scalar $k^{\prime }$ is a balancing factor; when the user-specific dataset $S_{i}$ is sufficiently large, a smaller value of $k^{\prime }$ may lead to improved personalization without overfitting.

Once trained, the resulting personalized model is denoted as $D({\vec{x}}_{r}|S_{i})$.


$$\begin{array}{c} D({\vec{x}}_{r}|S_{i})=argmin_{\theta }\;Pr\left(D({\vec{x}}_{r})\text{≠}y\;|\;S_{i}\right)+k^{\prime }Pr\left(D(\vec{x})\text{≠}y)\;|\;EEG_{pr}^{\prime }\right) \end{array}$$ (9)

It is important to note that the prior model is trained on augmented data $EEG_{pr}^{\prime }$ and is also evaluated on raw EEG signals $\vec{x}$. In contrast, the posterior model is fine-tuned using decompressed data and thus is optimized for evaluation on reconstructed signals ${\vec{x}}_{r}$, rather than raw data. This distinction justifies the use of $D({\vec{x}}_{r}|S_{i})$ instead of $D(\vec{x}|S_{i})$ in the left-hand side of the optimization objective in (9).

There are two approaches for deploying the posterior detector on the device:

1. **Evaluate on raw data** $\vec{x}$**:** Since the reconstruction loss $L_{r}$ in (6) forces the decompressed data to resemble the raw input, it is feasible to apply the posterior detector directly on raw signals. The trade-off between reconstruction fidelity and task-specific alignment is controlled by the factor *j*. In this scenario, the device uses $D(\vec{x}|S_{i})$ as a suboptimal approximation of the posterior detector.
2. **Evaluate using the optimal posterior detector** $D^{⋆}(\vec{x})$**:** This approach resolves the sub-optimality by explicitly passing the raw input through the learned compression–decompression pipeline before detection. The formulation is given in (10). Here, the task-aware penalty $L_{t}$ effectively acts as a preprocessing objective, which may improve classification accuracy. However, this setup requires deploying the decompressor on the device, thereby revealing its parameters and compromising the privacy benefits discussed earlier.

$$\begin{array}{c} D^{⋆}(\vec{x})=D(Decomp(Comp(\vec{x}))|S_{i}) \end{array}$$ (10)

Therefore, we recommend adopting the first approach for public applications, where privacy and scalability are priorities. In contrast, the second approach is more suitable for private, controlled, and limited-use cases where maximizing accuracy is critical. This distinction mirrors the paradigm of public cloud versus private cloud in conventional cloud computing frameworks.

Finally, the trained posterior model weights are transferred to the device and stored in its persistent memory. As a result, by the end of Step 7, the personalized posterior model becomes available locally on the device for real-time inference.

Even with the posterior model deployed, further improvements remain possible. However, to uphold ethical and privacy standards, explicit user consent is required before initiating additional updates, as illustrated in Step 8 of Fig 1. If the user agrees to participate, the system re-enters the adaptation cycle starting from Step 4, enabling continuous refinement of the posterior model.

The key characteristics of the proposed architecture are outlined below:

1. The architecture adopts a Bayesian perspective, emphasizing the importance of both the prior and posterior models. The prior model is not merely a pre-training artifact—it plays a vital role in device setup, connectivity, and post-factory deployment.
2. A Cloud-Edge architecture is targeted, which we consider a promising paradigm for BCI systems. In this design, the cloud handles computation-heavy and storage-intensive tasks, while the edge device is responsible for data compression and real-time model evaluation.
3. The use of lightweight models with transferrable weights is emphasized, considering that the evaluation is performed on embedded systems with limited computational resources.
4. Task-aware data compression is a key component, both for enhancing efficiency and for offering a degree of privacy by minimizing exposure of user-specific raw data—thereby contributing to the resolution of ethical challenges.

Therefore, we designate this architecture as **Bayesian Edge-cloud architecture with Lightweight classification and Task-aware compression (BELT)**.

### Model

Although the BELT architecture provides a conceptual framework for addressing key challenges in current BCI systems, it is not directly deployable without practical instantiation. In this section, we present our proposed implementation within the BELT architecture, built upon deep learning principles. Given the emphasis on lightweight design, we refer to this model as the **BELT-lite** model.

**Fig 2:**
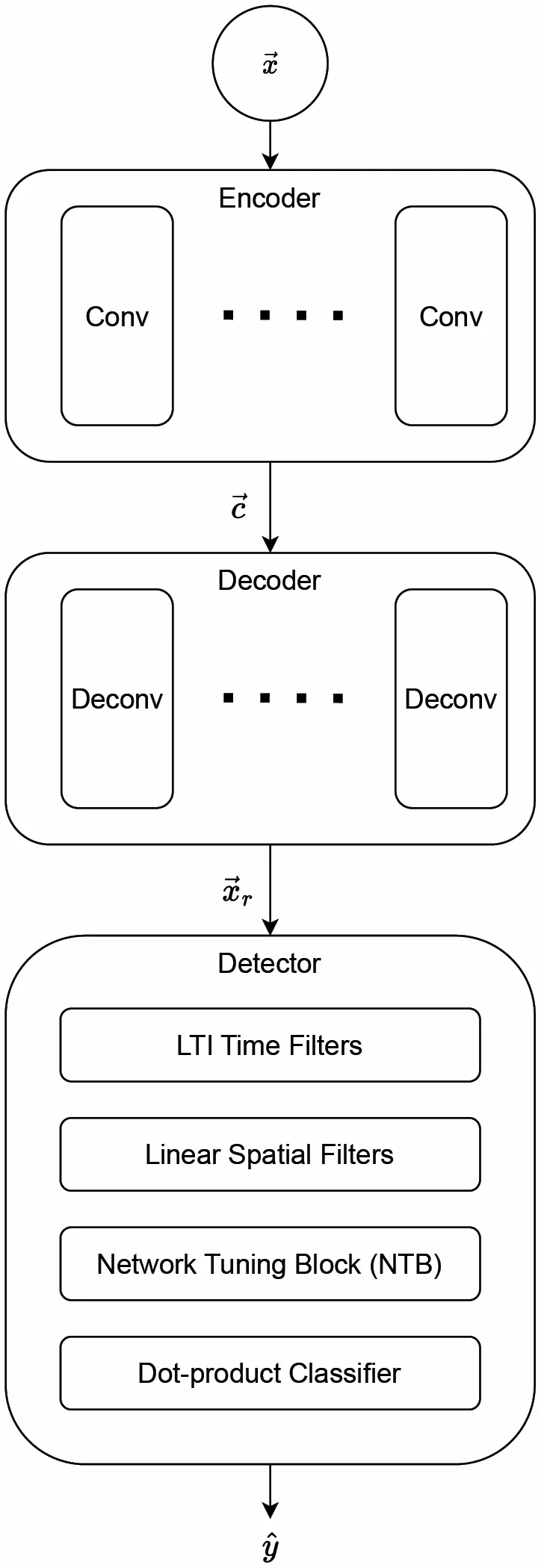
Overview of the BELT-lite model. The model consists of two main subsystems: a detector (bottom) for classification and an autoencoder (top) for data compression. The detector is optimized for embedded deployment and comprises four components: (i) linear time-invariant (LTI) temporal filters that extract frequency-specific features, (ii) linear spatial filters that combine information across channels, (iii) a Network Tuning Block (NTB) that computes log-power features and enables efficient two-phase training, and (iv) a lightweight dot-product classifier. The autoencoder compresses EEG signals for efficient edge–cloud transmission and can be used independently when compression is required. Together, these components realize a lightweight instantiation of the BELT architecture suitable for resource-constrained environments.

Despite being deep-learning-based, the BELT-lite model is optimized for embedded systems and is suitable for resource-constrained environments. An overview of the model is provided in Fig 2. As illustrated, we adopt an autoencoder architecture for data compression. The detector module consists of four core components: LTI time filters, linear spatial filters, a Network Tuning Block (NTB), and a dot-product classifier. The remainder of this section provides a detailed explanation of the detector design.

#### Detector.

The detector is implemented as a convolutional neural network with a dense (perceptron) tail and a nonlinear block between them. As illustrated in Fig 2, the detector is composed of four main subsystems:

1. **LTI Time Filters:** The multi-channel EEG input signal $\vec{x}$ is first passed through a set of Linear Time-Invariant (LTI) time filters, responsible for core time-domain signal processing. In the BELT-lite model, we adopt single-channel filtering; that is, each kernel operates exclusively on one specific channel of $\vec{x}$, with no cross-channel filtering at this stage.

The filters are implemented as Finite Impulse Response (FIR) filters of identical kernel size. Crucially, these kernels are not fixed or pre-designed—rather, their impulse responses are learned during prior model training and can be fine-tuned during the posterior training phase.

To implement LTI behavior within a neural framework, we employ one-dimensional convolutional (Conv1D) layers with the following constraints: linear activation, no bias term, and a stride of 1. This setup guarantees that the operation corresponds to a pure convolution with trainable, linear FIR filters.

The motivation for using LTI structures is rooted in hardware compatibility: LTI systems are natively supported by the Digital Signal Processing (DSP) cores often included in ARM Cortex-M microcontrollers and ARM Cortex-A processors, making this design highly suitable for embedded deployment [40].

2. **Linear Spatial Filters:** Multi-channel EEG signals contain valuable spatial information that is critical for effective classification [41,42]. To capture this, the second subsystem applies a set of linear spatial filters to the output of the time-filtered signals.

These spatial filters are implemented using Conv1D layers with the following constraints: linear activation function, kernel size of 1, stride of 1, and no bias term—ensuring strict linearity. The filters operate across all time-filtered channels simultaneously, allowing the model to extract spatial features through channel-wise mixing while preserving temporal alignment.

3. **Network Tuning Block (NTB):** In standard deep learning pipelines, convolutional layers are typically followed by a feature extractor (FE) block. In our architecture, we extend the role of this block by introducing additional objectives, renaming it to the *Network Tuning Block (NTB)*. The NTB is designed to fulfill the following purposes:
  (a) As highlighted by Yadav and Maini [6], BCI system design should incorporate insights from neurology, psychology, and physiology. Even in the context of deep learning—which traditionally offers a black-box approach to feature extraction—these domain-specific insights should be leveraged through informed network design, architectural choices, and hyperparameter tuning.
  (b) The NTB facilitates the separation of optimization responsibilities between the prior and posterior training phases. Specifically, posterior training is intended to fine-tune primarily the final classifier, while leaving earlier layers (e.g., LTI time filters and linear spatial filters) relatively unchanged. Conversely, the prior training phase focuses on optimizing these earlier components. Enforcing this behavior requires careful formulation, which is handled internally by the NTB.

Ultimately, the NTB functions as the main feature extractor. However, unlike standard FE blocks, it is designed with the aforementioned objectives in mind. Its output is a task-relevant feature vector passed to the final classification stage.

4. **Classifier:** Given the extracted feature vector, the symbol decoding task is framed as a classification problem. To ensure efficiency and compatibility with embedded systems, we adopt a minimalist yet expressive design: a domain transform followed by a correlation-based classification per target symbol.

The domain transform is implemented via a dense (fully connected) layer. Subsequently, each symbol is associated with a dedicated dot product operation, forming a lightweight linear classifier. This structure not only reduces computational overhead but also offers flexibility for adaptation during posterior training.

This two-layer structure strikes a balance between expressive power and efficiency. From a neural network perspective, this architecture is justified as two-layer perceptrons are known to be universal function approximators. From a telecommunications perspective, the first layer performs a transformation to a symbol space, while the second layer performs a distance-like operation. Although this is not a strict Euclidean (*l*_2_) distance as used in classical telecommunication systems, the dot product serves as a generalized similarity measure and is compatible with signal-space interpretations.

### Implementation

To support reproducibility and address the well-documented challenge of re-implementation in BCI research, we provide the complete source code for the BELT framework. The implementation is archived and publicly available at: https://github.com/adanayi/BELT.

#### EEG dataset and task framing.

We used EEG recordings from the BCI Competition IV-2b and BCI competition IV-2a datasets which are both widely adopted benchmarks for MI-based brain–computer interface research [43,44].

The BCI Competition IV-2b dataset includes data from nine healthy subjects, each performing two-class MI tasks (left hand vs. right hand) across five sessions, with 120 trials per session—resulting in a total of 600 trials per subject. The recordings comprise three bipolar EEG channels (C3, Cz, C4) positioned over the sensorimotor cortex, along with three monopolar EOG channels used for artifact monitoring. In this study, only the EEG channels were used, in line with our focus on developing methods suitable for low-channel, EEG-only headsets that are more practical for real-world deployment.

The BCI Competition IV-2a dataset similarly includes recordings from nine healthy subjects, but involves four-class MI tasks (left hand, right hand, both feet, and tongue) [44]. Each subject participated in two sessions conducted on different days, with each session consisting of 288 trials (72 per class). Although the original recordings used 25 EEG channels and three EOG channels, we selected only the three sensorimotor cortex channels (C3, Cz, C4) to maintain consistency with our low-channel configuration and also used two classes (left hand and right hand) to align with the IV-2b experimental setup.

To create a larger and more diverse subject pool, we combined both datasets. Subjects 1–9 correspond to the original subjects from the BCI Competition IV-2b dataset, while subjects 10–18 correspond to the nine subjects from the BCI Competition IV-2a dataset. This combined cohort enables cross-dataset evaluation and increases the statistical power of our analysis.

The BCI Competition IV datasets include trial rejection flags indicating segments affected by artifacts or acquisition errors. According to the dataset documentation, trials were marked as rejected if the EEG signal contained visible muscle artifacts, eye movements, or technical disturbances identified through visual inspection by experienced annotators. We adhered to these provided rejection flags and applied no additional artifact rejection or trial removal beyond the dataset’s own annotations, ensuring consistency with prior benchmarks. After removing rejected trials, we retained 7613 valid segments from a combined pool of 9112 total trials, representing a retention rate of approximately 83%. Each segment spans the active motor imagery period from *t* = 3 s to *t* = 7 s post-cue onset. With a sampling rate of *Fs* = 250 Hz, each segment contains 1000 time-domain samples.

Modern deep learning frameworks—including those incorporating recurrent architectures—typically assume pre-segmented inputs. Yet, our literature review revealed that segmentation procedures are rarely described in sufficient detail, hindering reproducibility. To address this, we provide both the pre-segmented datasets and the segmentation code in the GitHub repository accompanying this work. All code uses fixed random seeds, ensuring that all stochastic operations are fully reproducible.

#### Cross validation strategy.

To ensure statistical reliability and reproducibility, we fixed the random seed before any data splitting. Shuffling is essential in this dataset because each subject’s recordings span five sessions, and later segments may be more informative as subjects improve with visual feedback. Standard *k*-fold splits that preserve session order could unintentionally bias certain folds; shuffling yields *k* independent realizations of the MI-EEG process and mitigates this risk.

We employ two complementary cross-validation (CV) strategies, each serving a distinct evaluation goal.

**Subject-dependent 10-fold CV (full prior training):** All 9112 segments from all 18 subjects are pooled, shuffled, and randomly split into 10 folds, each containing approximately 90% training and 10% validation segments. This procedure is repeated identically for every experiment, using the same pre-computed fold assignments. Per-subject accuracy is then computed on the validation segments belonging to each subject. Because training and validation folds may contain different segments from the same subject, this strategy evaluates the model’s capacity to fit the population under identical training conditions, *not* its ability to generalise to completely unseen subjects. It is used for all comparisons of architectures, augmentation levels, and compression methods, where the primary interest is relative performance.

**Leave-one-subject-out with repeated folds (new-subject prior training):** To explicitly measure generalisation to new users, we conduct a separate experiment in which one subject is held out entirely. The prior model is trained on data from the remaining 17 subjects, and the held-out subject serves exclusively as the validation set. To obtain robust performance estimates despite the small per-subject sample size, this training-validation split is repeated 10 times for each held-out subject, using different random shuffles of the 17 subjects’ data (still under the fixed random seed for consistency). This yields a mean and standard deviation of the prior accuracy for each subject when treated as unseen. Posterior fine-tuning is subsequently performed using only the held-out subject’s data, mirroring a real-world onboarding scenario.

In both strategies, the validation segments are strictly excluded from the training set in each fold, so there is no data leakage. The term *validation accuracy* throughout the paper refers to the metric computed on these held-out segments.

**Fig 3:**
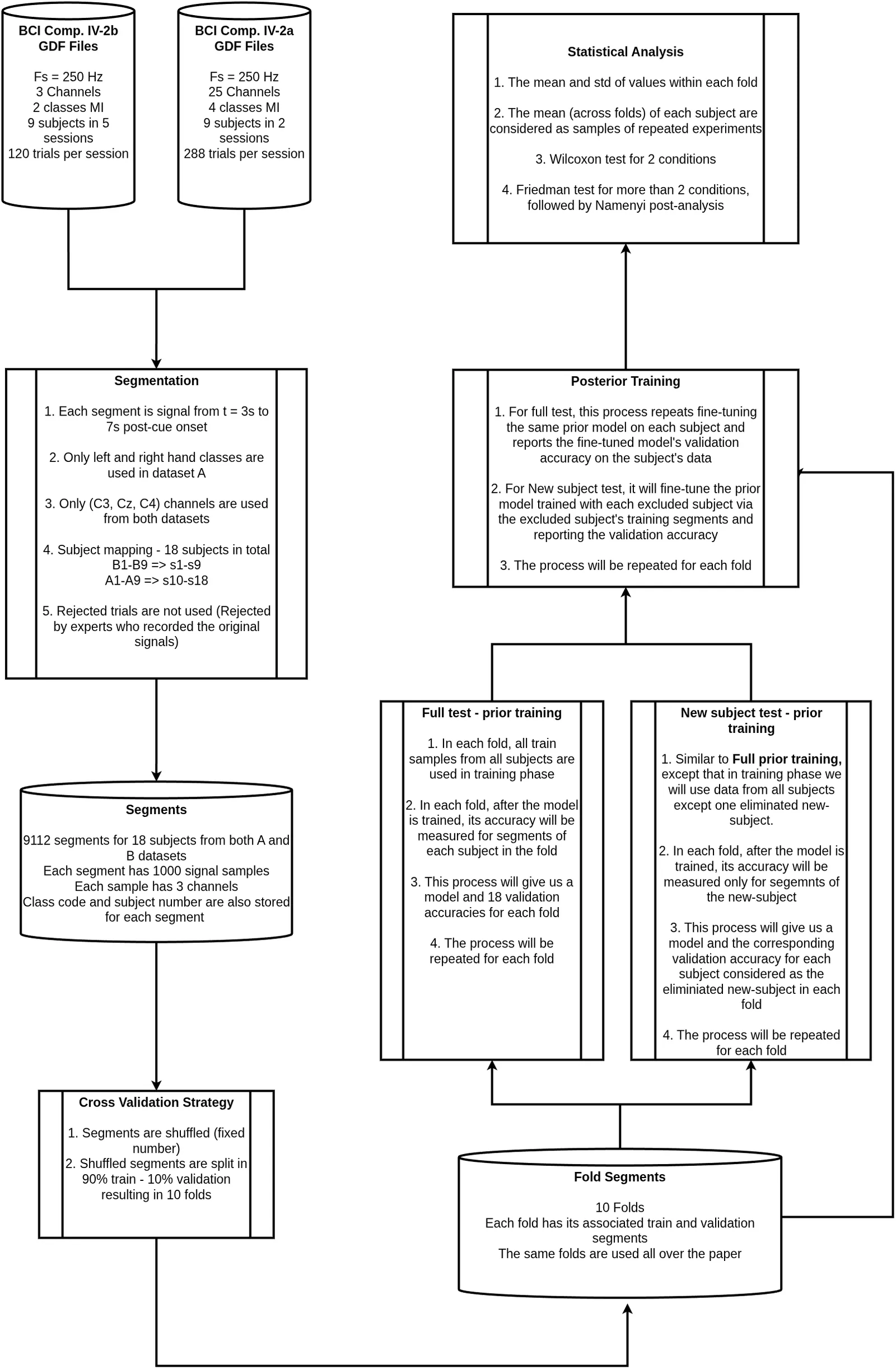
Complete data analysis pipeline. The flowchart illustrates the end-to-end experimental workflow from raw data to statistical inference, including dataset combination, segmentation, cross-validation, evaluation scenarios (full training and new-subject testing), posterior fine-tuning, and statistical testing methodology. Details of each stage are provided in the accompanying text.

Fig 3 summarises the complete analysis pipeline from raw data to statistical inference, highlighting both CV branches.

#### Statistical tests methodology.

For statistical analysis, per-subject mean accuracies and standard deviations are computed across the 10 folds. These subject-level means serve as repeated measures for hypothesis testing: Wilcoxon signed-rank tests are used for pairwise comparisons (two conditions), and Friedman tests followed by Nemenyi post-hoc analysis are used for comparisons involving three or more conditions. All tests are applied to the 18 subject-level means, properly accounting for inter-subject variability.

#### Detector.

The detector was implemented using the Keras framework [45]. The network input is a tensor of shape (ξ,1000,3), where ξ denotes the variable batch size. The first layer applies LTI time filtering, implemented as a grouped Conv1D layer with 9 kernels and a groups parameter of 3. This configuration yields 3 filters per EEG channel (3 channels total). The kernel size is 25 samples, corresponding to 100ms at a sampling frequency of $F_{s}=250\;Hz$. Using ‘same’ padding, this layer outputs a tensor of shape (ξ,1000,9).

Following this, the linear spatial filtering block is another Conv1D layer with 16 kernels of size 1 sample. Both the LTI time and spatial filter layers have no bias terms and employ linear (identity) activation functions. Consequently, the output of the spatial filtering block has shape (ξ,1000,16), which is then passed to the Network Tuning Block (NTB).

The NTB begins with an element-wise square operation defined as


$$\begin{array}{c} y(\vec{x})=[x_{1}^{2},x_{2}^{2},...,x_{N}^{2}] \end{array}$$ (11)

followed by a 1D average pooling layer, and finally a logarithmic transform given by


$$\begin{array}{c} y(\vec{x})=\left[\log(\epsilon +x_{1}),\log(\epsilon +x_{2}),...,\log(\epsilon +x_{N})\right] \end{array}$$ (12)

where ϵ is set to 10^−8^ to ensure numerical stability.

The average pooling layer uses a window size of 20 samples with a stride of 13 samples, resulting in smoothed power estimates over approximately 80ms frames with 52ms overlap.

The choice of signal power as a classification feature is motivated by findings that the power in the mu rhythm and central β bands conveys MI information [8,46].

As shown in our prior work [13], this log-power structure enables a two-phase training procedure: during early stages with larger errors, backpropagation modifies the weights of preceding layers (prior training phase), whereas in later stages with smaller errors, backpropagation is effectively suppressed to minimize changes in these layers and focus training on the subsequent classifier layers (posterior training phase). This behavior aligns well with the intended training regime of the BELT-lite model.

This log-power structure fulfills the two additional objectives outlined for the NTB. While the use of log power is common in MI-BCI literature, its effectiveness is often empirically noted without a clear explanation. Our analysis clarifies that the superior performance stems from the fulfillment of these specific training objectives. Although integrating more recent physiological insights into the NTB may further enhance performance, this work focuses on emphasizing the importance of these objectives rather than exploring alternative architectures.

It is worth mentioning that allocating three LTI filters per channel is also motivated by findings regarding the μ rhythm [8] and central β sub-bands [46] in MI classification. Accordingly, two of the filters are expected to approximate bandpass filters for the μ and β frequency bands, while the third filter provides an additional degree of freedom for data-driven adaptation.

The use of 80ms overlapping frames for power smoothing is motivated by the need to reduce the number of classifier parameters in small-dataset scenarios—such as ours—to mitigate overfitting during training.

The output of the NTB is a tensor of shape (ξ,76,16), which is then flattened into a vector of shape (ξ,1216) before being fed into the classifier’s first layer. Through the combined processing of the LTI filters, spatial filters, and NTB, the input signal size is effectively reduced to approximately 40.5% of its original dimensions ((ξ,1000,3)).

The classifier itself is a two-layer perceptron comprising 4 neurons in the first layer and 2 neurons in the second. The first layer uses a ReLU activation function, while the second layer employs a sigmoid activation, making it suitable for one-hot encoded classification tasks.

Given the limited size of the dataset, we employed several strategies to mitigate overfitting. Batch normalization layers were applied both before and after the NTB. Additionally, an *l*_2_ regularization term with a penalty coefficient of 0.01 was applied to the first dense layer of the classifier. A dropout layer with a dropout probability of 0.2 was inserted between the classifier’s dense layers.

We contend that the most complex layer in the model is the first dense layer of classifier, where each neuron connects to 1216 inputs from the previous layer. This high connectivity increases the risk of overfitting given the limited dataset. However, as discussed earlier, this layer is intended to learn the signal space or domain transformation; thus, we allow it substantial representational capacity while relying on *l*_2_ regularization to control overfitting.

Since the final classifier layer performs a distance-like measurement in the transformed space, we prefer the preceding layer to maintain expressive power rather than excessive compression to preserve generalization. Consequently, the dropout layer placed between the classifier layers plays a critical role in balancing expressiveness and regularization.

The structure of the detector is summarized in Table 2. The layers are listed in order, along with their Keras naming conventions, to facilitate reproducibility and ease of reimplementation by the reader.

**Table 2:** BELT-lite detector layer structure.

Layer name	Type	Structure	Activation
Input	Input	Input shape: (ξ,1000,3)	
LTI-Time-Filts	Conv1D	Kernels: 9 – size: 25 – groups: 3	linear
Spatial-Filts	Conv1D	Kernels: 16 – size: 1	linear
Pre-NTB	BatchNormalization	–	–
NTB-square	Lambda	Element-wise *y* = *x*^2^	–
NTB-avg	AveragePooling1D	Size: 20 – stride: 13	–
NTB-log	Lambda	Element-wise y=log(ϵ+x)	–
NTB-flat	Flatten	–	–
Post-NTB	BatchNormalization	–	–
Classifier-l1	Dense	4 Neurons with l2 norm	ReLU
Classifier-DO	Dropout	Dropout term: 0.2	–
Classifier-lo	Dense	2 Neurons	Sigmoid

Detailed layer-by-layer architecture of the BELT-lite detector module, presented in execution order using Keras terminology to facilitate reproducibility. The input shape is (ξ,1000,3) where ξ is the batch size. Total trainable parameters: approximately 1.65k. LTI layers use linear activation with no bias terms to ensure compatibility with DSP hardware. The NTB (Network Tuning Block) implements log-power feature extraction through square, average pooling, and log operations. The classifier consists of a dense layer with *l*_2_ regularization (coefficient 0.01), dropout (rate 0.2), and a final sigmoid output layer for binary c*l*assification.

For prior detector training, we used batch size 32, learning rate 10^−3^, and 100 epochs with 10-fold cross-validation (the same folds were used across all $a_{aug}$ levels).

#### Data augmentation.

To enhance training, we developed a conditional GAN (cGAN) to synthesise realistic EEG signals. The generator takes a 100-dimensional latent vector concatenated with a one-hot class label, passes it through three dense layers (32 units, ReLU), then a dense layer (3000 units, tanh) reshaped to 1000×3 (three EEG channels). The class label is repeated across time and concatenated, yielding a 1000×5 output.

The discriminator processes a 1000×5 input. EEG channels go through two convolutional layers (18 filters, kernel 25; then 16 filters, kernel 1; linear, no bias), batch norm, custom squaring, average pooling (20, stride 13), custom logarithm, flattening and batch norm. Class channels are flattened separately. Both are concatenated and fed through dense layers (256→4 units) with leaky ReLU (α=0.2) and dropout (0.2), ending with a sigmoid unit.

The cGAN was trained per fold on training splits only, using Adam (lr=$2\times 10^{-4}$, $\beta_{1}=0.5$) and binary cross-entropy for 300 epochs. Batch size was one-fifth of the training set (300 samples). All models were implemented in TensorFlow/Keras and trained on an RTX 2060 GPU running Ubuntu 24.04. For prior detector training, we used batch size 32, learning rate 10^−3^, and 100 epochs with 10-fold cross-validation.

The augmentation parameter $a_{aug}$ controls the number of synthetic samples generated per real training sample (e.g., $a_{aug}=2$ triples the dataset size). Within each cross-validation fold, we trained five BELT-lite models using $a_{aug}\in \{0,1,2,3,4\}$, resulting in five models per fold, each with a different augmentation level. Augmented data were used only for prior model training, not for posterior fine-tuning, reflecting real-world constraints where subject-specific GANs are infeasible at scale.

#### Compressor.

We have used an AutoEncoder for the compression task. While AutoEncoders are already widely adopted in EEG signal processing, the value of our approach lies in its alignment with the practical-friendly architecture of BELT via BELT-lite. Specifically, we rely on a convolution-only structure, which, as discussed earlier, is well-suited for embedded systems and real-time cloud applications. We emphasize that the current AutoEncoder design is not intended as an optimal solution, but rather as a functional and extensible starting point within the scope of this work.

The Encoder network consists of three Conv1D layers. The first layer uses 32 kernels of size 3 and stride 1, enabling it to extract 32 short temporal-spatial patterns without compressing the time dimension. The second layer has 16 kernels of size 25 (i.e., 100ms) and a stride of *E*_1_, introducing compression in the time domain. The third layer applies *N* kernels of size 2*E*_2_ with stride *E*_2_, further compressing the temporal axis. Notably, the number of parameters in this final layer grows with *N*, allowing greater expressivity when compression is high.

Letting *C* denote the number of EEG channels, the overall compression ratio *CR* is calculated as:


$$\begin{array}{c} CR=\frac{CE_{1}E_{2}}{N} \end{array}$$ (13)

For instance, with *C* = 3, *N* = 9, *E*_1_ = 5, and *E*_2_ = 2, we achieve a compression ratio of *CR* = 3.33. In contrast, we checked the ZIP standard compression and it yields a much lower ratio of 1.04 for this dataset while being unsuitable for real-time use, and being inefficient for implementation on low-cost embedded systems. Although our method is lossy, unlike ZIP, this is not inherently a drawback—it necessitates evaluating the impact of compression on final task performance.

The Decoder consists of two deconvolution (Conv1DTranspose) layers. The first layer uses 32 kernels of size $2E_{1}E_{2}$ and stride $E_{1}E_{2}$, effectively upsampling the compressed representation back to the original temporal resolution. The second layer applies *C* kernels of size 3 to reconstruct the multi-channel EEG signal. The first layer performs the main decompression, while the second maps the output to the original channel dimensionality.

Kernel size in the first layer is deliberately set proportional to the compression parameters, as higher compression typically demands greater capacity for accurate reconstruction. All encoder and decoder layers use linear activation, and unlike the classifier, we allow bias terms in all layers. This added complexity is acceptable because (1) compression is not always required in BELT, and (2) when compression is active, classification is bypassed, allowing us to tolerate this trade-off for improved reconstruction quality.

### Comparative baseline model: EEGNet

To evaluate the computational efficiency and accuracy of BELT-lite, we selected EEGNet [47] as a baseline model. EEGNet is a compact convolutional neural network specifically designed for EEG-based brain–computer interfacing. It employs depthwise and separable convolutions to achieve high classification performance with a minimal parameter count (≈2.23k parameters), making it a widely adopted benchmark for lightweight BCI decoding architectures. Its balance of accuracy and efficiency makes it an appropriate reference point for assessing BELT-lite’s suitability for embedded deployment.

While EEGNet’s inference latency is acceptable for many laboratory setups, on ultra-low-power platforms such as the ARM Cortex-A7 it still consumes significant CPU time; further reduction is desirable for prolonged battery-operated use.

For all pairwise comparisons between BELT-lite and EEGNet, we employed the Wilcoxon signed-rank test as the primary statistical measure. This nonparametric test was chosen because it makes no assumptions about the normality of the underlying data distribution and is appropriate for paired observations (same subjects or same experimental runs). For accuracy comparisons, the test was applied to the mean accuracies of each subject across 10 cross-validation folds. For runtime comparisons, we performed 100 independent measurement runs, each consisting of 100 consecutive inferences, and used the Wilcoxon test to compare the resulting latency distributions. All tests were two-tailed with a significance threshold of α=0.05. Results are reported as mean ± standard deviation unless otherwise noted, and effect sizes are discussed in the context of practical significance.

## Results

### Prior detector

We trained the prior detector on all 18 subjects using the augmentation scheme described in the Data Augmentation subsection. Table 3 reports the per-subject accuracy (mean ± std) for each augmentation level.

**Table 3:** Prior model accuracy across augmentation levels.

Subject	$a_{aug}=0$	$a_{aug}=1$	$a_{aug}=2$	$a_{aug}=3$	$a_{aug}=4$
*s*_1_	70.8±7.1	75.7±6.2	71.9±6.2	73.7±7.2	75.5±8.0
*s*_2_	64.5±5.4	66.3±6.7	67.2±3.6	68.1±6.5	69.4±5.1
*s*_3_	61.6±8.6	64.4±7.0	67.0±4.9	66.9±7.5	65.9±8.0
*s*_4_	90.6±3.3	88.7±3.8	90.6±4.1	91.4±3.3	89.2±4.9
*s*_5_	81.8±2.6	84.2±4.1	84.5±4.0	84.6±3.9	83.9±2.2
*s*_6_	72.7±2.6	79.9±5.8	78.1±6.0	78.9±6.8	80.1±6.0
*s*_7_	78.7±8.8	82.0±4.2	81.6±6.8	84.2±6.9	81.1±4.8
*s*_8_	75.7±5.2	77.4±5.9	77.7±6.0	76.1±5.8	76.1±4.9
*s*_9_	71.4±7.7	76.5±7.7	76.3±7.6	77.4±6.6	75.6±7.9
**Mean (B)**	74.2±8.8	77.2±7.9	77.2±7.8	77.9±7.9	77.4±7.1
*s*_10_	54.7±10.9	57.9±10.1	57.2±7.8	52.1±10.3	59.1±7.6
*s*_11_	48.1±11.2	51.7±10.6	45.8±6.9	48.8±13.1	48.4±11.7
*s*_12_	72.2±13.0	74.4±9.7	72.5±11.0	73.1±10.8	76.2±12.8
*s*_13_	56.6±12.0	52.0±8.9	58.6±8.7	57.3±11.9	53.9±8.4
*s*_14_	58.4±12.3	49.9±5.4	50.4±6.9	52.6±11.3	53.8±9.1
*s*_15_	51.5±11.3	53.3±11.8	51.5±16.6	51.9±14.9	55.6±9.3
*s*_16_	53.2±11.6	55.0±6.4	56.0±14.2	55.5±14.7	49.3±11.9
*s*_17_	59.8±12.0	60.8±10.6	60.2±12.2	59.5±11.0	57.5±13.3
*s*_18_	54.8±14.2	55.6±11.5	59.3±9.7	54.5±11.2	53.6±12.4
**Mean (A)**	56.6±6.8	56.7±7.4	56.8±7.6	56.2±7.1	56.4±8.2

Per-subject classification accuracy (%, mean ± standard deviation across 10 cross-validation folds) for prior models trained with different augmentation factors $a_{aug}\in \{0,1,2,3,4\}$. Subjects 1–9 correspond to Dataset B (two-class MI), and Subjects 10–18 correspond to Dataset A (four-class MI reduced to two classes). Bold rows indicate dataset-wise means. A Friedman test across the five fixed augmentation levels showed a significant overall effect ($\chi^{2}(4)=12.04$, *p* = 0.017). Nemenyi post-hoc comparisons revealed that $a_{aug}=3$ significantly outperformed the baseline $a_{aug}=0$ (adjusted *p* = 0.019), while $a_{aug}=1$ showed borderline significance (*p* = 0.048); no other fixed level differed significantly from the baseline.

To assess whether augmentation significantly influences performance, we conducted a Friedman test on the mean accuracies across all subjects, treating each fixed $a_{aug}$ level (0–4) as repeated measures. The test indicated a significant overall effect of augmentation intensity ($\chi^{2}(4)=12.04$, *p* = 0.017). A Nemenyi post-hoc test was then performed to identify which specific conditions differed.

The pairwise comparisons revealed that $a_{aug}=3$ significantly outperformed the baseline $a_{aug}=0$ (adjusted *p* = 0.019), and $a_{aug}=1$ showed a borderline significant improvement (*p* = 0.048). No other fixed augmentation level differed significantly from the baseline (all *p* > 0.05), and none of the non-zero levels ($a_{aug}\in \{1,2,3,4\}$) differed significantly from each other (adjusted p≈1.0 in all cases).

These findings lead to two nuanced conclusions:

1. Simply applying a fixed augmentation level across all subjects does *not* guarantee a statistically significant improvement over using only real data. While levels $a_{aug}=3$ (and, to a marginal extent, $a_{aug}=1$) provided reliable gains, other intensities – particularly $a_{aug}=2$ and $a_{aug}=4$ – did not reach significance. The variability across folds and subjects dampens the effect of many augmentation strengths, so the choice of intensity matters.
2. There is no evidence that any single fixed augmentation level is universally superior; the optimal intensity likely varies per subject. The significant improvement seen with $a_{aug}=3$ suggests that moderate augmentation is beneficial, and subject-specific tuning remains a promising direction for future work.

Notably, even the best fixed augmentation level ($a_{aug}=3$) remained below the highest single-subject accuracy observed in this study (e.g., subject S4 achieved 93.6±2.9%). This underscores that inter-subject variability remains a more fundamental challenge than the choice of augmentation intensity, and that real-world BCI systems will still require careful per-subject calibration.

### Posterior detector

The prior detector is then fine-tuned individually on each subject’s own data to get the posterior detector. While this follows the standard train-then-fine-tune paradigm in transfer learning and operates on point estimates rather than full probability distributions, we retain the terminology of *prior* and *posterior* to suggest the possibility of extending this framework into a fully Bayesian formulation in future work. Although this is not true Bayesian inference, the terminology anticipates the integration of Bayes’ rule as envisioned in the BELT architecture.

Please note that this fine-tuning approach does not explicitly implement the optimization defined in (9). However, the selection of training epochs and learning rates during the prior and posterior phases can be interpreted as implicit controls similar to the $k^{\prime }$ factor, balancing the influence of subject-specific data against general prior knowledge. Accordingly, no fixed numeric value is assigned to $k^{\prime }$ in our experiments. In our setup, the prior model is trained with a learning rate of 10^−3^ on data from all 18 subjects (or 17, depending on the experiment), whereas posterior fine-tuning uses a higher learning rate of $5\times 10^{-3}$ on a single subject’s data. The drastically reduced dataset size inherently limits the influence of the subject-specific loss and, together with the learning rate, provides a regularising effect analogous to a small $k^{\prime }$.

Table 4 reports the posterior model accuracies for all 18 subjects under two prior augmentation conditions: no augmentation ($a_{aug}=0$) and moderate augmentation ($a_{aug}=1$). Fine-tuning used the same fixed prior checkpoint obtained from training with the respective augmentation level. To assess whether augmentation during prior training influences posterior performance, we applied a Wilcoxon signed-rank test to the per-subject accuracies. The test revealed a significant difference between the two conditions (*W* = 30.0, *p* = 0.0139). Concretely, fine-tuning from the moderately augmented prior yielded a mean posterior accuracy of 84.3% across all subjects, compared to 82.6% for the no-augmentation prior — an average improvement of approximately 1.7 percentage points. On Dataset A the benefit is more pronounced: mean accuracy rose from 77.4% to 80.6%, while on Dataset B the means were identical (87.9%). This result shows that even a modest amount of synthetic prior data can provide a statistically reliable benefit for subject-specific adaptation, although the effect varies by dataset.

**Table 4:** Posterior model accuracy across augmentation levels.

Subject	$a_{aug}=0$	$a_{aug}=1$
*s*_1_	88.6±4.1	87.3±3.6
*s*_2_	74.6±4.6	74.8±5.1
*s*_3_	80.8±4.7	82.9±4.7
*s*_4_	97.9±2.1	98.0±1.9
*s*_5_	93.1±3.7	93.6±3.3
*s*_6_	92.1±3.6	92.1±4.0
*s*_7_	90.0±3.1	91.0±2.9
*s*_8_	88.0±2.7	86.2±2.7
*s*_9_	85.9±5.3	85.0±6.2
**Mean (B)**	87.9±6.9	87.9±6.8
*s*_10_	71.8±5.3	74.9±6.9
*s*_11_	65.7±7.1	70.3±6.1
*s*_12_	94.2±5.3	94.2±4.0
*s*_13_	70.9±5.3	74.5±9.0
*s*_14_	74.1±10.8	78.7±7.9
*s*_15_	65.1±8.7	71.5±12.1
*s*_16_	79.7±9.2	85.3±5.6
*s*_17_	85.9±4.2	86.3±5.3
*s*_18_	89.1±7.7	89.9±6.0
**Mean (A)**	77.4±10.4	80.6±8.6

Per-subject classification accuracy (%, mean ± standard deviation across 10 cross-validation folds) for posterior models fine-tuned from priors trained with two different augmentation levels. Subjects 1–9 correspond to Dataset B (two-class MI), and Subjects 10–18 correspond to Dataset A (four-class MI reduced to two classes). Bold rows indicate dataset-wise means. A Wilcoxon signed-rank test comparing $a_{aug}=0$ and $a_{aug}=1$ across all subjects indicates a significant difference (*W* = 30.0, *p* = 0.0139), with the augmented prior yielding higher overall accuracy (mean 84.3% vs. 82.6%).

Once again, we emphasize that our use of the term *posterior* does not refer to Bayesian posterior in the strict sense. However, we argue that a two-stage approach—either explicitly Bayesian or inspired by Bayesian reasoning—is essential. Both models serve distinct purposes: the *prior* model is useful for initial deployment or onboarding new users, while the *posterior* model is crucial for long-term use, as subject-specific fine-tuning significantly boosts accuracy.

**Fig 4:**
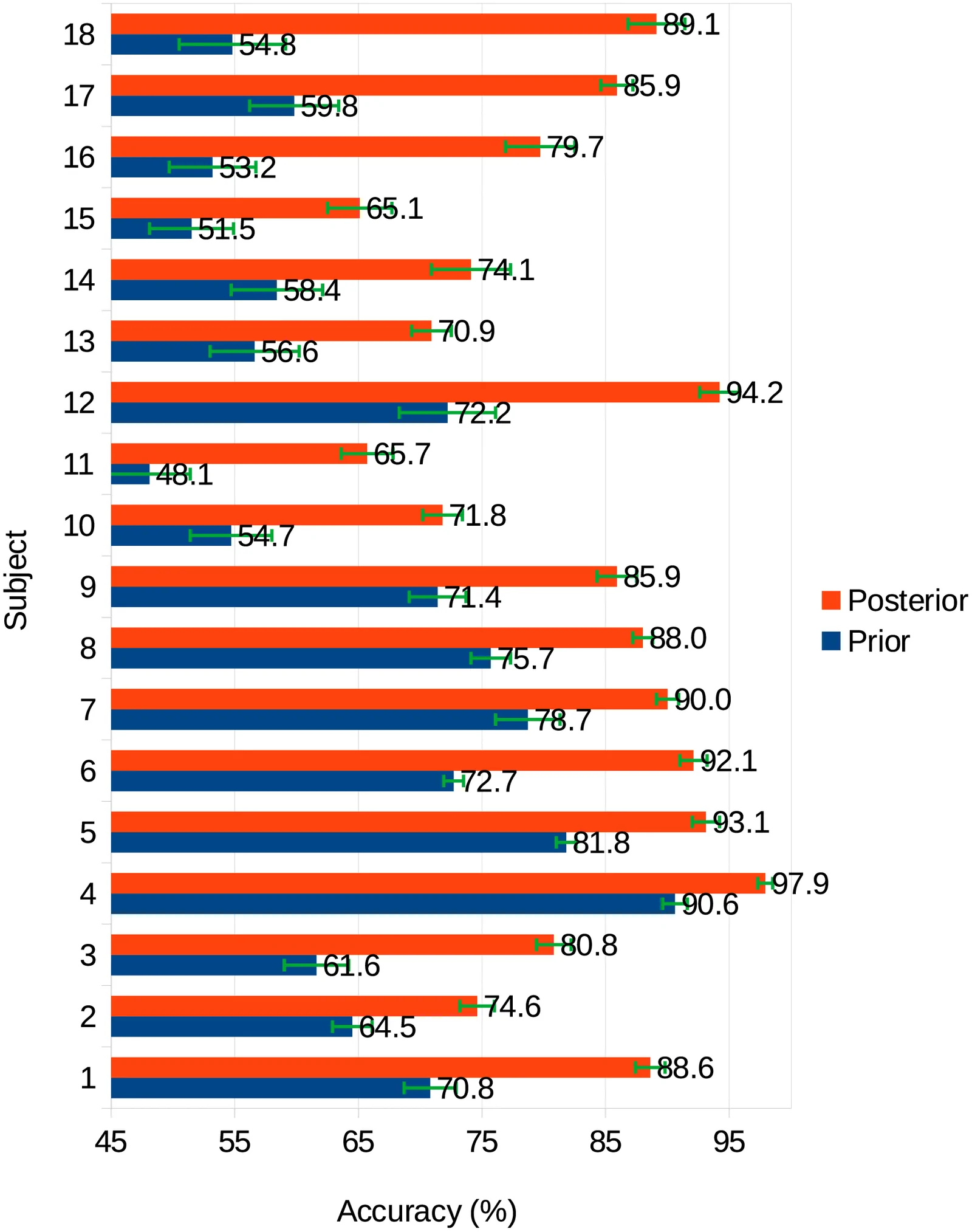
Mean prior and posterior classification accuracy for each subject ($a_{aug}=0$). Bars represent mean accuracy across 10 cross-validation folds, with error bars indicating standard error of the mean (SEM). Results are shown for all 18 subjects (Subjects 1–9 from Dataset B, Subjects 10–18 from Dataset A). Posterior fine-tuning consistently improves accuracy across every subject, with gains ranging from approximately 5 to 25 percentage points. Notably, Subject 4 (Dataset B) approaches near-perfect accuracy (97.9%±2.1%) after adaptation, while Subject 12 (Dataset A) reaches 94.2%±5.3%, demonstrating the effectiveness of subject/user training. The substantial gap between prior and posterior accuracies across all subjects underscores the necessity of user-specific adaptation in practical BCI deployment.

Comparing prior and posterior results (Table 3 vs. Table 4) confirms that posterior training is essential for achieving high accuracy and should not be omitted in practical applications. The substantial gap between prior and posterior accuracies across all subjects underscores the necessity of user-specific adaptation. This is also shown in Fig 4.

To formally assess the value of posterior training relative to prior models, we conducted a Friedman test across four conditions:

- Prior ($a_{aug}=0$)
- Prior ($a_{aug}=1$)
- Posterior ($a_{aug}=0$)
- Posterior ($a_{aug}=1$)

**Fig 5:**
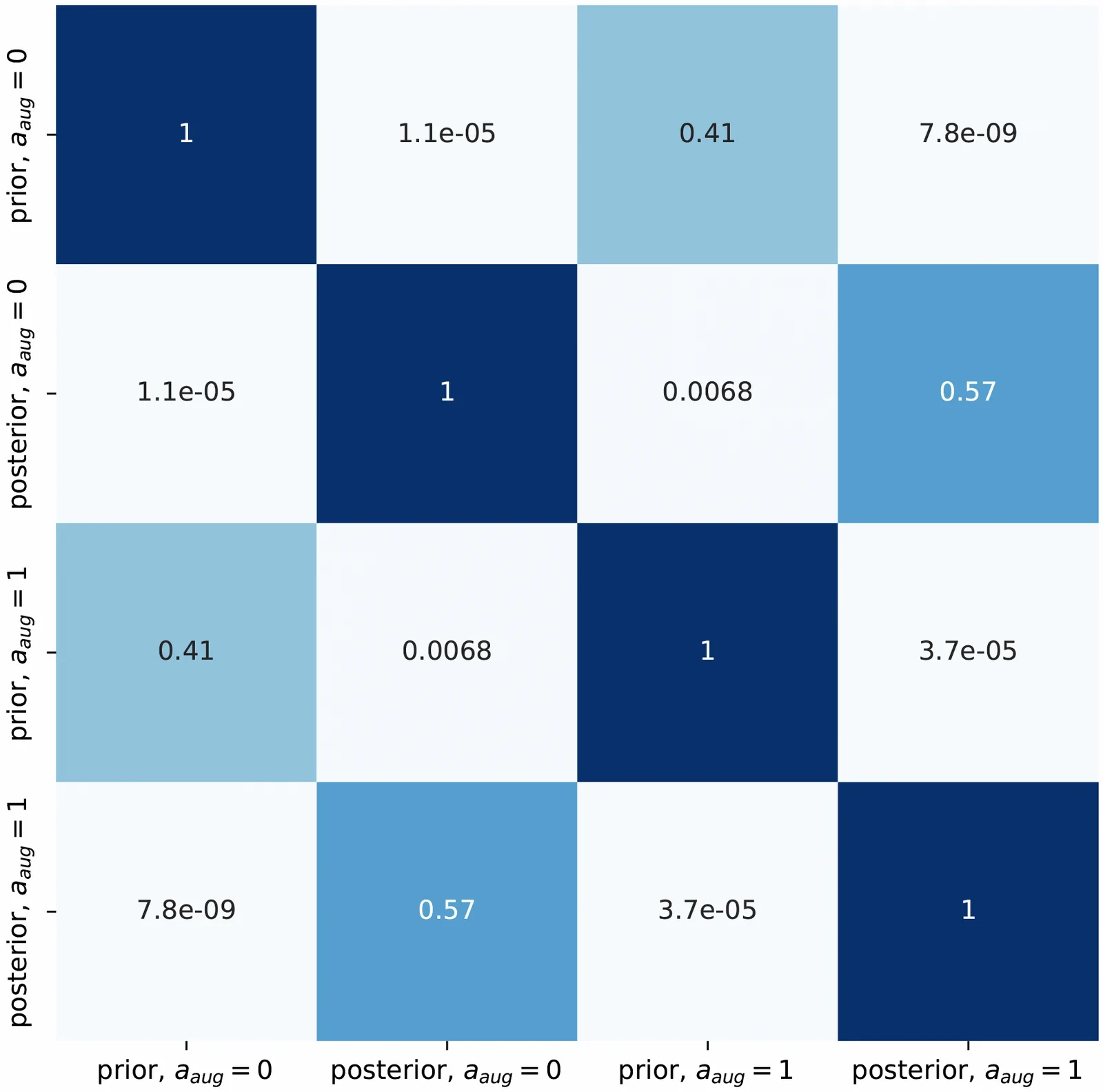
Nemenyi post-hoc test results comparing prior and posterior models under different augmentation conditions. The heatmap compares four conditions: Prior ($a_{aug}=0$), Prior ($a_{aug}=1$), Posterior ($a_{aug}=0$), and Posterior ($a_{aug}=1$). Each colored cell indicates that the row condition does not significantly outperform the column condition (*p* < 0.05) based on the Nemenyi test following a significant Friedman test ($\chi^{2}(3)=47.27$, $p=3.05\times 10^{-10}$). Key findings: (i) both posterior conditions significantly outperform both prior conditions, confirming that subject-specific fine-tuning is essential; (ii) the two prior conditions do not differ significantly; (iii) the two posterior conditions do not differ significantly after correction for multiple comparisons, though a direct pairwise Wilcoxon test indicated a modest significant difference (*p* = 0.0139); thus the effect of prior augmentation on posterior accuracy is small and not robust in all statistical contexts.

The test revealed a highly significant overall difference ($\chi^{2}(3)=47.27$, $p=3.05\times 10^{-10}$). Fig 5 presents the Nemenyi post-hoc results as a significance heatmap. Three key findings emerge:

1. Both posterior conditions significantly outperform both prior conditions (all adjusted *p* < 0.01, see heatmap). This confirms that subject-specific fine-tuning is essential for maximizing accuracy, regardless of the prior augmentation level.
2. The two prior conditions ($a_{aug}=0$ and $a_{aug}=1$) do not differ significantly from each other (*p* > 0.05). While the dedicated augmentation analysis in previous subsection indicated that some augmentation levels (e.g., $a_{aug}=3$) can significantly improve prior performance, the more limited difference between $a_{aug}=0$ and $a_{aug}=1$ is not detectable in this four-condition comparison after correction for multiple testing.
3. The two posterior conditions ($a_{aug}=0$ and $a_{aug}=1$) did not differ significantly in the Nemenyi post-hoc test (*p* > 0.05). This appears to contradict the earlier Wilcoxon test restricted to the two posterior groups (which gave *p* = 0.0139), but the discrepancy arises because the Nemenyi test applies a conservative correction for all pairwise comparisons across the four conditions. The effect of prior augmentation on posterior performance, while detectable in a focused pairwise comparison, is modest and does not remain significant under this broader correction. We therefore conclude that once fine-tuning is applied, the choice of prior augmentation level has at most a small benefit, which should be weighed against the simplicity of using no augmentation.

**Fig 6:**
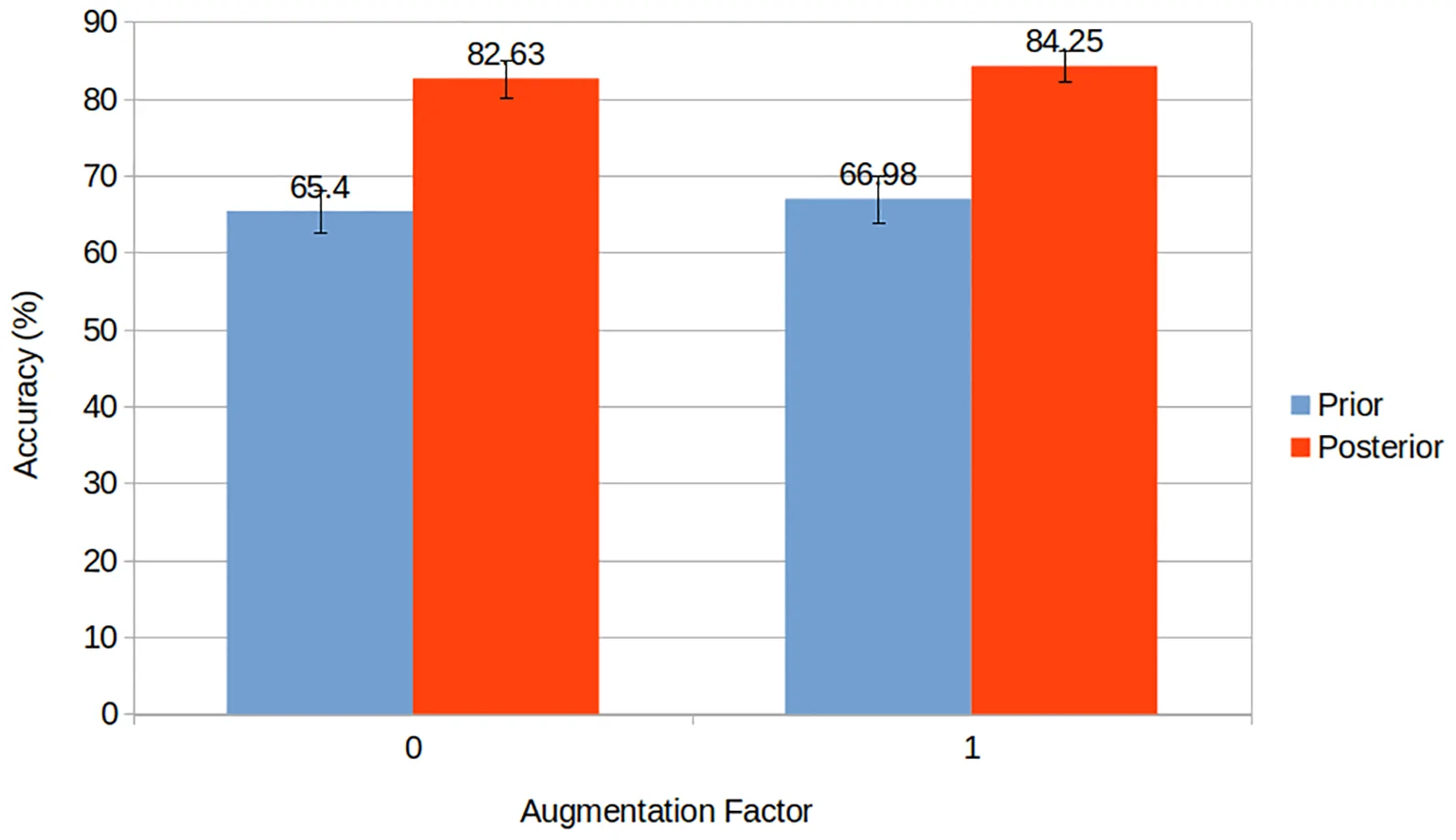
Mean prior and posterior accuracies across all 18 subjects for augmentation levels $a_{aug}=0$ and $a_{aug}=1$. Error bars indicate standard error of the mean (SEM). Posterior accuracies substantially exceed prior accuracies for both conditions, while the difference between the two augmentation levels is small and discussed in the text.

To illustrate the findings, Fig 6 shows the mean prior and posterior accuracies across all 18 subjects for the two augmentation levels $a_{aug}=0$ and $a_{aug}=1$, with error bars representing the standard error of the mean (SEM). The figure confirms that posterior accuracies consistently exceed prior accuracies for both augmentation levels, consistent with the significant effect of fine-tuning reported above.

### Training optimization

In order to reduce the computational load on the cloud, we propose to partially limit fine-tuning by freezing parts of the model. This section presents the implementation of this approach along with its results. Before presenting the results, we highlight the potential advantages of freezing parts of the model during training:

1. **Reduced computational load:** Fewer parameters are updated, leading to lower back-propagation overhead, which reduces processing demand on cloud infrastructure.
2. **Lower memory requirements:** The end device requires less programmable memory, as it no longer needs to store the coefficients of the LTI filter layers.
3. **ASIC compatibility:** Fixed LTI filter coefficients allow for hardware implementation using ASICs, which can significantly improve efficiency.
4. **Improved generalization with limited data:** A simpler model, resulting from partial freezing, is less prone to overfitting when the posterior training data is scarce. While this advantage may diminish with larger datasets, in practice, the posterior data must be collected during the initial user setup. Therefore, minimizing the required data collection enhances the user experience.

#### Frozen LTI filter layers.

As a first step, we freeze only the LTI filters layer of the model, allowing the spatial and classifier layers to be fine-tuned. This choice is motivated by the fact that LTI filters are generic task-dependent temporal feature extractors and may not require subject-specific adaptation, whereas spatial and classifier layers can benefit from personalization. By freezing only the LTI layers, we aim to balance model adaptability with computational efficiency.

The results are shown in Table 5. A Wilcoxon signed-rank test comparing the two prior augmentation levels ($a_{aug}=0$ vs. $a_{aug}=1$) revealed a significant improvement when fine-tuning from the augmented prior (*W* = 7.0, $p=1.45\times 10^{-4}$). Concretely, the dataset-wise means increased from 86.4% to 87.2% on Dataset B and from 75.5% to 78.8% on Dataset A.

**Table 5:** Posterior accuracy with frozen LTI filter fine-tuning.

Subject	$a_{aug}=0$	$a_{aug}=1$
1	85.3±3.4	85.7±3.1
2	74.6±4.8	76.1±3.9
3	80.1±4.2	82.5±4.8
4	97.9±2.0	97.9±1.9
5	89.3±3.1	91.0±2.8
6	90.4±3.7	89.9±4.1
7	89.4±4.2	90.5±2.8
8	87.1±3.4	86.9±3.4
9	83.8±3.7	84.5±5.9
**Mean (B)**	86.4±6.6	87.2±6.1
10	68.3±7.1	71.5±6.5
11	65.5±7.8	70.7±6.7
12	88.7±8.7	89.7±4.6
13	67.3±6.4	70.6±7.1
14	74.5±8.5	75.3±7.6
15	64.7±11.0	71.0±10.3
16	78.6±9.5	85.0±3.3
17	83.2±5.7	85.5±4.1
18	88.7±6.3	90.0±6.0
**Mean (A)**	75.5±9.7	78.8±8.6

Per-subject classification accuracy (%, mean ± standard deviation across 10 cross-validation folds) for posterior models where only LTI filter layers were frozen during fine-tuning, while spatial filters and classifier layers were allowed to adapt. Results are shown for two prior augmentation conditions: no augmentation ($a_{aug}=0$) and moderate augmentation ($a_{aug}=1$). Subjects 1–9 correspond to Dataset B (two-class MI), and Subjects 10–18 correspond to Dataset A (four-class MI reduced to two classes). Bold rows indicate dataset-wise means. A Wilcoxon signed-rank test indicates that the augmented prior ($a_{aug}=1$) yields significantly higher accuracy than the non-augmented prior (*W* = 7.0, $p=1.45\times 10^{-4}$). Compared to full fine-tuning (Table 4), freezing LTI layers results in a negligible accuracy drop of 0.5−1.5 percentage points, validating the NTB design for efficient adaptation.

#### Classifier only.

In the next step, both the LTI filters and the spatial linear filters are frozen during posterior training, allowing only the classifier layers to adapt. This configuration aims to further reduce the computational load while evaluating whether the remaining trainable layers can compensate for reduced flexibility. The results are presented in Table 6.

**Table 6:** Posterior accuracy with classifier-only fine-tuning.

Subject	$a_{aug}=0$	$a_{aug}=1$
1	83.9±4.8	82.4±4.3
2	75.4±5.6	76.3±3.6
3	79.5±4.1	81.2±5.1
4	96.9±2.8	97.0±2.6
5	88.7±3.9	90.3±4.0
6	89.0±2.7	89.4±3.0
7	89.8±3.9	89.6±1.8
8	86.0±3.8	86.2±3.0
9	84.0±6.8	84.0±6.9
**Mean (B)**	85.9±6.2	86.3±6.1
10	66.7±7.6	68.3±7.7
11	68.0±9.7	68.7±7.6
12	86.9±8.2	85.4±4.1
13	67.7±5.8	71.4±6.0
14	71.1±8.9	73.7±9.6
15	64.7±10.5	70.1±10.3
16	77.2±7.6	82.4±5.3
17	80.4±6.1	77.1±11.0
18	87.7±7.4	88.7±7.6
**Mean (A)**	74.5±8.9	76.2±7.6

Per-subject classification accuracy (%, mean ± standard deviation across 10 cross-validation folds) for the most constrained fine-tuning configuration, where both LTI filters and spatial linear filters are frozen, allowing only the classifier layers to adapt. Results are shown for two prior augmentation conditions: no augmentation ($a_{aug}=0$) and moderate augmentation ($a_{aug}=1$). Subjects 1–9 correspond to Dataset B (two-class MI), and Subjects 10–18 correspond to Dataset A (four-class MI reduced to two classes). Bold rows indicate dataset-wise means. A Wilcoxon signed-rank test confirms that the augmented prior ($a_{aug}=1$) yields significantly higher accuracy than the non-augmented prior (*W* = 7.0, $p=1.45\times 10^{-4}$). Compared to full fine-tuning (Table 4), classifier-only fine-tuning incurs a modest accuracy drop of 2−5 percentage points—an acceptable trade-off for resource-constrained deployment scenarios.

A Wilcoxon signed-rank test between the two prior augmentation levels ($a_{aug}=0$ vs. $a_{aug}=1$) revealed a significant improvement when fine-tuning from the augmented prior (*W* = 7.0, $p=1.45\times 10^{-4}$). Concretely, the dataset-wise means increased from 85.9% to 86.3% on Dataset B and from 74.5% to 76.2% on Dataset A.

### Summary of fine-tuning depth

**Fig 7:**
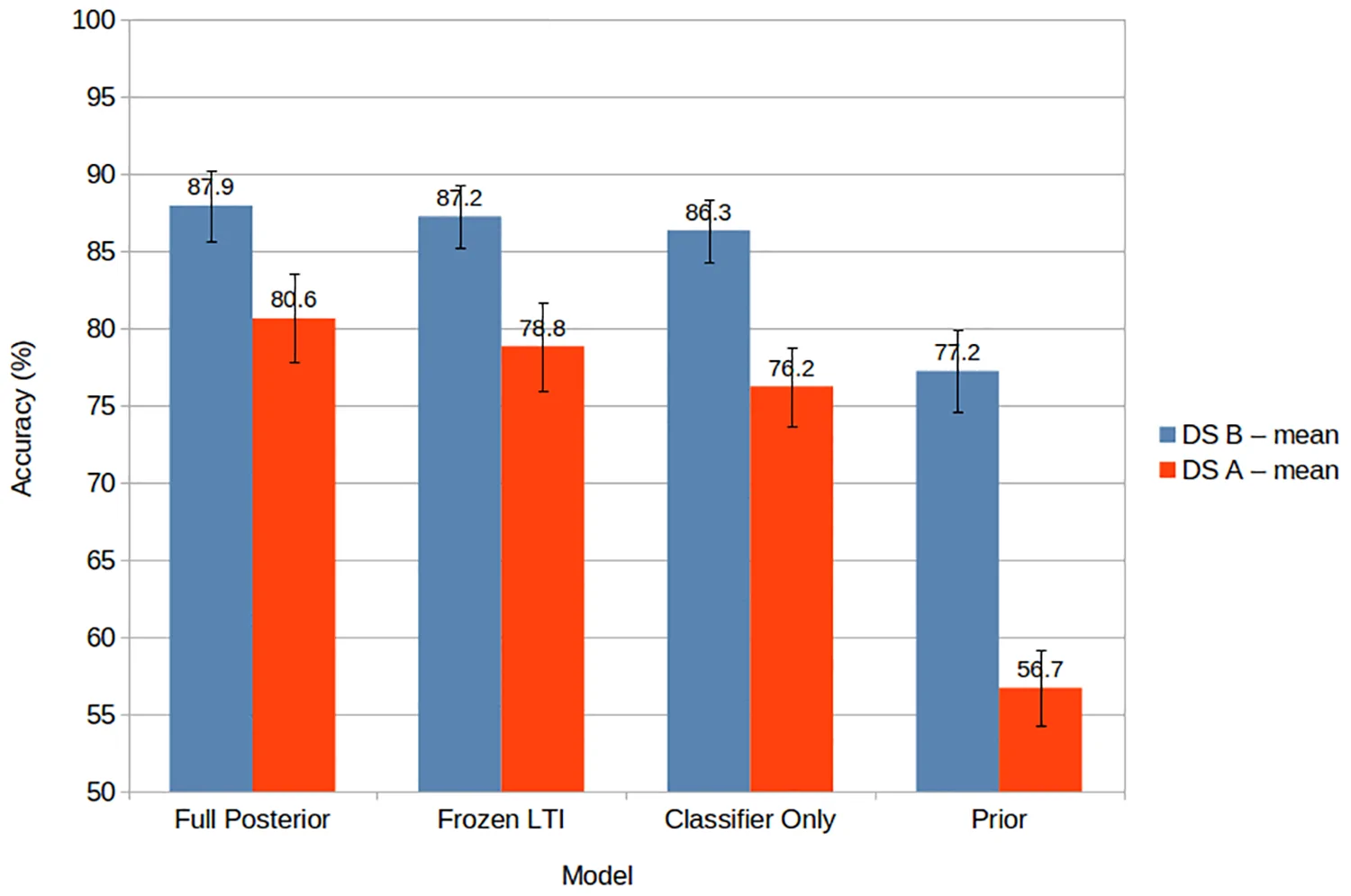
Effect of fine-tuning depth on posterior accuracy. Paired bars show mean accuracy across 18 subjects for BCI Competition IV-2a and IV-2b datasets. All posterior results use the augmented prior ($a_{aug}=1$). The prior bar is included for visual reference. Error bars indicate SEM. A Friedman test on the three posterior conditions reveals a significant effect of fine-tuning depth ($\chi^{2}(2)=22.11$, $p=1.58\times 10^{-5}$); full fine-tuning significantly outperforms classifier-only training, while frozen LTI does not differ significantly from full fine-tuning (see Fig 8).

To compare the three posterior adaptation strategies, we fixed the prior augmentation level at $a_{aug}=1$—the intensity that yielded the highest mean accuracy in the previous analyses—and evaluated each subject’s accuracy after full fine-tuning, frozen LTI, and classifier-only training. Fig 7 shows the mean accuracy for Dataset B and Dataset A under all four conditions, including the prior model for reference. Error bars denote the standard error of the mean (SEM) across subjects.

**Fig 8:**
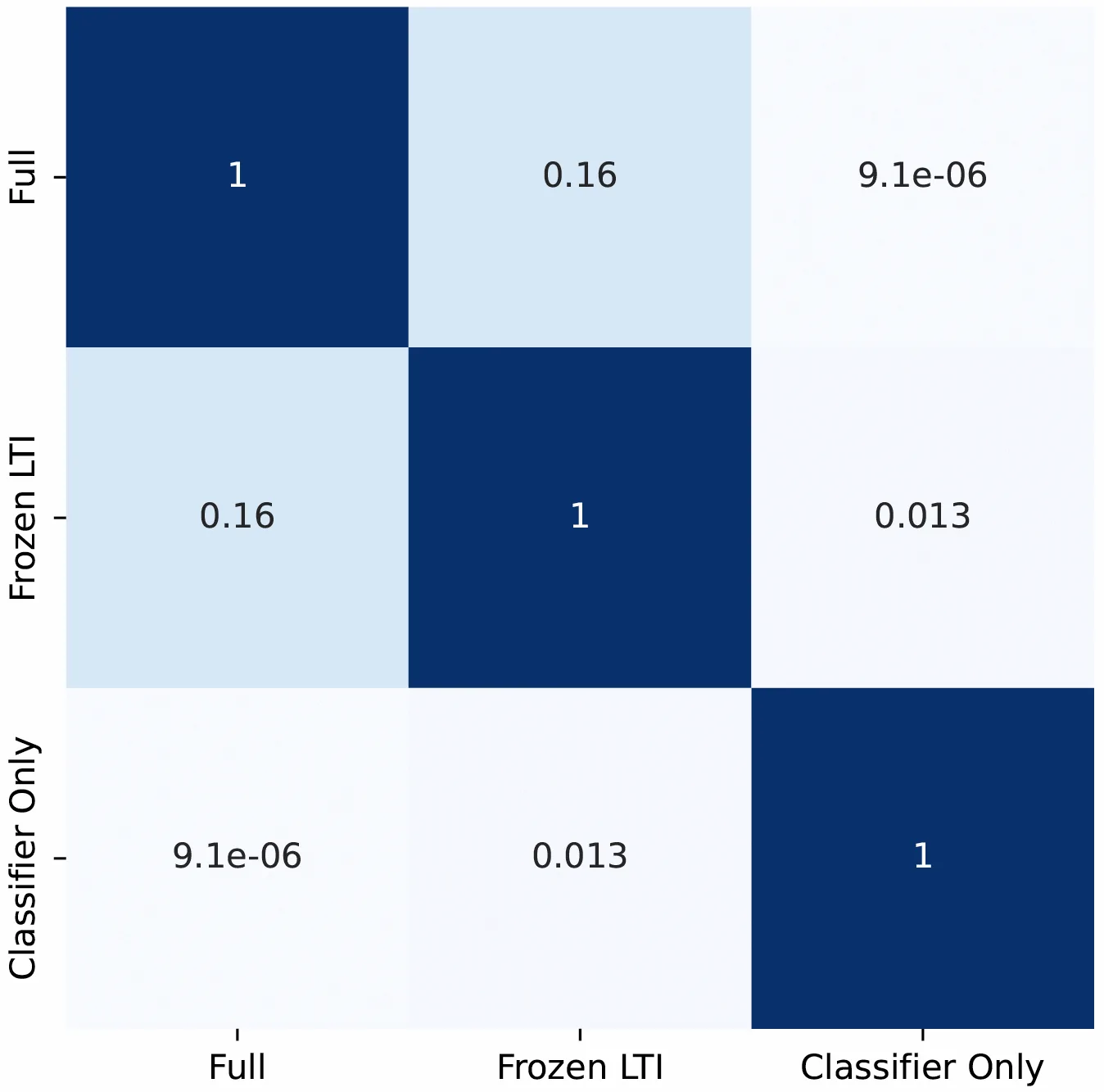
Nemenyi post-hoc test results for fine-tuning depth. The heatmap compares three posterior conditions (all using $a_{aug}=1$): full fine-tuning, frozen LTI layers, and classifier-only fine-tuning. Colored cells indicate that the row condition significantly outperforms the column condition (*p* < 0.05) after a significant Friedman test ($\chi^{2}(2)=22.11$, $p=1.58\times 10^{-5}$). Full fine-tuning significantly outperforms classifier-only ($p=9.1\times 10^{-6}$); frozen LTI is not significantly different from full (*p* = 0.16) but significantly better than classifier-only (*p* = 0.013).

A Friedman test on the three posterior conditions across all 18 subjects confirmed a significant effect of fine-tuning depth ($\chi^{2}(2)=22.11$, $p=1.58\times 10^{-5}$). Nemenyi post-hoc tests (Fig 8) revealed that full fine-tuning significantly outperforms classifier-only fine-tuning (adjusted $p=9.1\times 10^{-6}$), while the frozen LTI configuration does not differ significantly from full fine-tuning (*p* = 0.16). Notably, frozen LTI is significantly better than classifier-only training (*p* = 0.013).

These results indicate that freezing only the LTI filters preserves most of the accuracy of full fine-tuning, whereas restricting adaptation to the classifier alone incurs a statistically significant but practically modest penalty of 2–5 percentage points. Given the substantial computational savings—classifier-only fine-tuning updates only a fraction of the network parameters—this trade-off is acceptable for resource-constrained deployment scenarios, while frozen LTI provides an intermediate configuration that is statistically indistinguishable from the full model.

### New subject performance

In this experiment, we evaluate the architecture’s ability to generalize to new and unseen subjects. Due to limited data, we adopt a leave-one-subject-out approach: each time, one subject is excluded from the prior model training, and the system is later fine-tuned (posterior) using only the excluded subject’s data. Additionally, to better understand the generalization behavior, we measure the performance of subject 4 (as a good performing subject due to Fig 4) across all trained models. To reduce complexity and save time, we disable the data augmentation step and report results only for $a_{aug}=0$.

**Fig 9:**
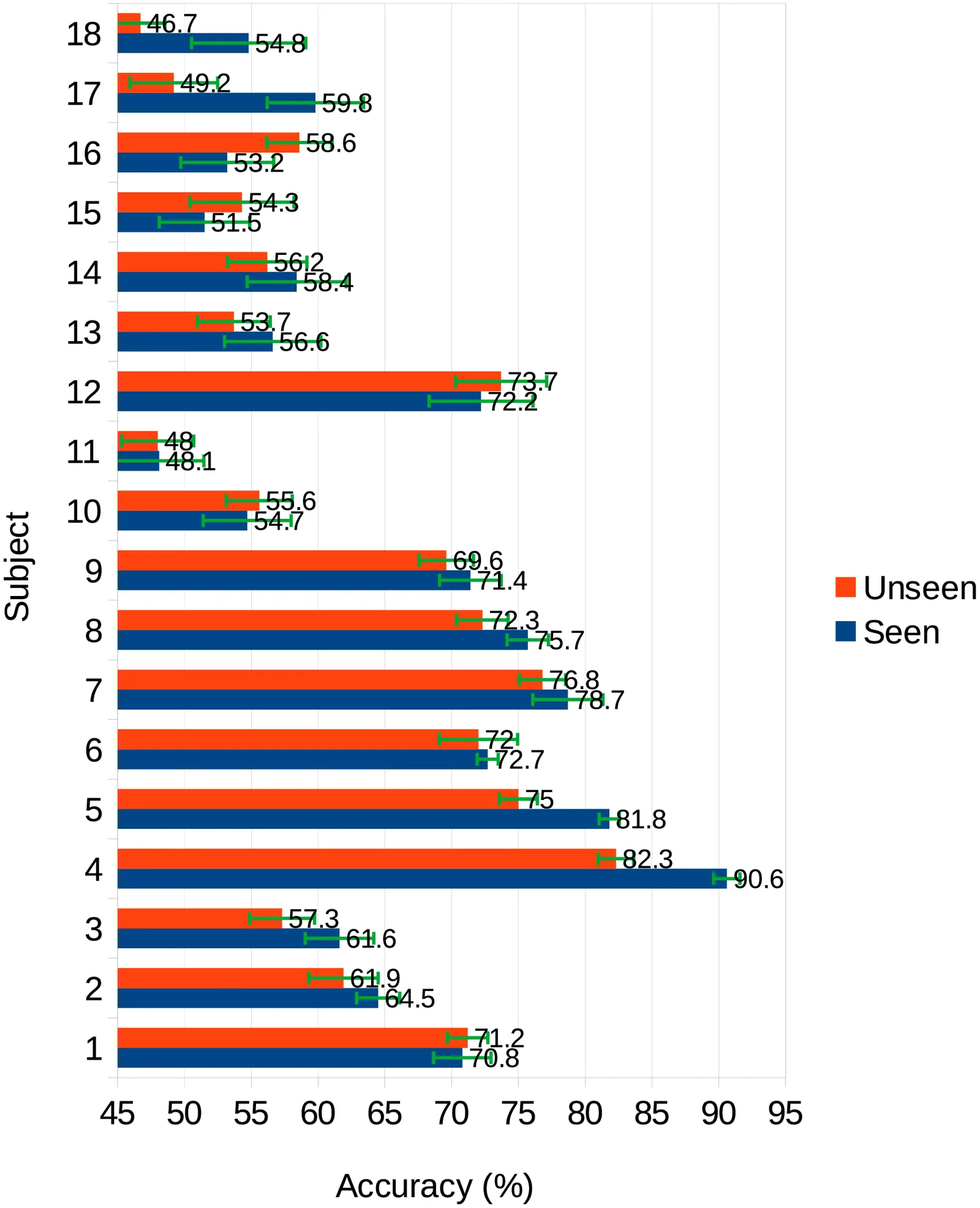
Comparison of prior model accuracy on seen vs. unseen subjects across 18 subjects. Bars represent mean accuracy with error bars indicating standard error of the mean (SEM). “Seen” refers to models trained with the target subject’s data included (results from Table 3), while “Unseen” refers to leave-one-subject-out evaluation where the target subject was excluded during prior training. Results show a statistically significant accuracy drop for unseen subjects (Wilcoxon signed-rank test: *W* = 35.0, *p* = 0.027, *N* = 18), with mean decreases of 3.3% for Dataset B and 1.5% for Dataset A. This demonstrates the generalization limitations of BCI models and underscores the necessity of subject-specific data exposure even in the prior training phase.

The performance of the prior model is reported in Table 7 and depicted in Fig 9. To quantify the cost of encountering a new subject, we compared each subject’s prior accuracy when treated as unseen (from the leave-one-subject-out procedure) against their own prior accuracy when they were part of the full prior training, as reported in Table 3 (column $a_{aug}=0$). A Wilcoxon signed-rank test on these 18 paired observations revealed a significant drop in performance (*W* = 35.0, *p* = 0.027, *N* = 18). The mean accuracy decreases were 3.3 percentage points for Dataset B and 1.5 percentage points for Dataset A. This confirms that excluding a subject from the prior training introduces a small but statistically significant penalty, even though subsequent fine-tuning can partially compensate, as will be discussed.

**Table 7:** Performance of prior models in the new subject test in percent (μ±σ format).

New subject	Accuracy for new subject	Accuracy for subject 4
*s*_1_	71.2±5.1	89.2±3.5
*s*_2_	61.9±8.6	89.3±3.6
*s*_3_	57.3±8.1	87.7±5.2
*s*_4_	82.3±4.4	82.3±4.4
*s*_5_	75.0±4.7	88.5±3.4
*s*_6_	72.0±9.8	86.3±4.6
*s*_7_	76.8±5.8	89.5±2.9
*s*_8_	72.3±6.4	91.5±3.9
*s*_9_	69.6±6.8	89.1±5.2
**Mean (B)**	70.9±7.5	88.2±2.6
*s*_10_	55.6±8.2	87.9±5.6
*s*_11_	48.0±9.0	87.1±2.7
*s*_12_	73.7±11.4	89.1±4.2
*s*_13_	53.7±9.1	88.1±3.6
*s*_14_	56.2±9.9	87.7±4.7
*s*_15_	54.3±13.0	89.3±2.8
*s*_16_	58.6±8.2	87.1±4.5
*s*_17_	49.2±11.0	87.1±3.8
*s*_18_	46.7±6.1	89.1±4.4
**Mean (A)**	55.1±8.1	88.1±0.9

Each row corresponds to a prior model trained on all subjects except one. The second column reports the accuracy of that model when tested on the excluded subject, simulating a new user scenario. The third column reports the same model’s accuracy on a fixed reference subject (Subject 4) to verify that model quality remains consistent across training runs—variation here would indicate training instability rather than genuine differences in new-subject performance.

We do not make a conclusion regarding the effect of each individual subject on the model at this point, but we strongly indicate the necessity of having larger prior datasets. We also expect that more generalized models with fewer parameters can reduce the dependency on certain subjects beyond the current level although they open the challenge of under-fitting.

**Fig 10:**
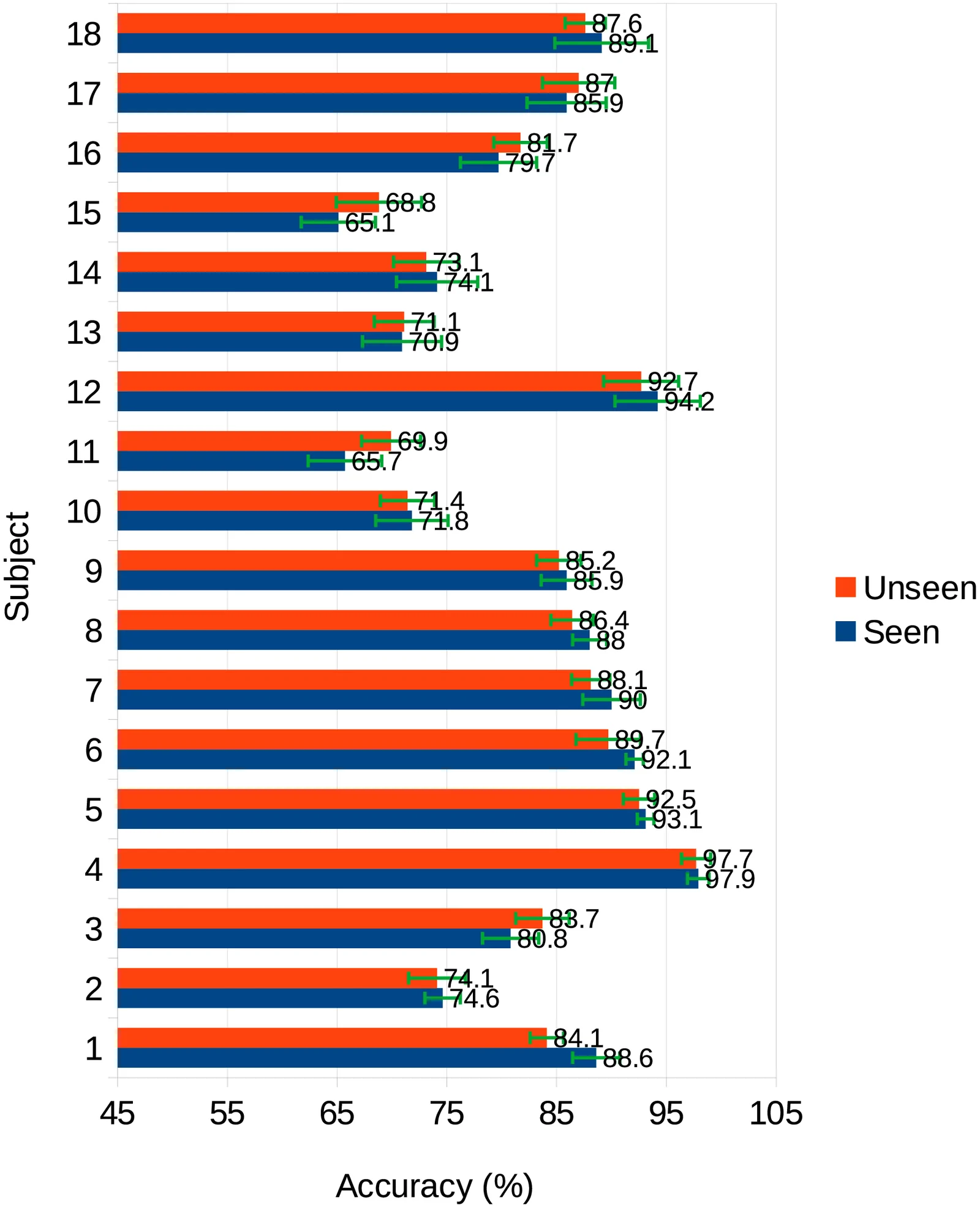
Comparison of posterior model accuracy on seen vs. unseen subjects across 18 subjects. Bars represent mean accuracy after posterior fine-tuning, with error bars indicating standard error of the mean (SEM). “Seen” refers to models where the target subject’s data was included in prior training before fine-tuning; “Unseen” refers to models where the target subject was excluded during prior training and fine-tuned only on their own held-out data. On average, unseen subjects showed a 1.1% decrease in accuracy for Dataset B and a 0.8% increase for Dataset A compared to seen subjects. A Wilcoxon signed-rank test revealed that these differences are not statistically significant (*W* = 70.0, *p* = 0.523), indicating that posterior fine-tuning is robust to new subjects and does not degrade significantly even when prior exposure is absent—a desirable outcome supporting the practical viability of the BELT architecture.

The results for posterior training are presented in Table 8 and illustrated in Fig 10. On average, accuracy decreased by 1.1% for Dataset B and, surprisingly, increased by 0.8% for Dataset A when subjects were unseen during prior training and subsequently fine-tuned in the posterior phase. However, a Wilcoxon signed-rank test revealed that these differences are not statistically significant (W = 70.0, p = 0.523). This indicates that posterior training performance does not degrade significantly for new subjects—a desirable outcome that supports the robustness of the proposed approach.

**Table 8:** Performance of posterior models in the new subject test in percent (μ±σ format).

New subject	Accuracy for subject
*s*_1_	84.1±5.3
*s*_2_	74.1±5.6
*s*_3_	83.7±5.0
*s*_4_	97.7±1.9
*s*_5_	92.5±2.5
*s*_6_	89.7±3.8
*s*_7_	88.1±3.6
*s*_8_	86.4±4.0
*s*_9_	85.2±5.6
**Mean (B)**	86.8±6.5
*s*_10_	71.4±8.9
*s*_11_	69.9±5.4
*s*_12_	92.7±3.8
*s*_13_	71.1±6.4
*s*_14_	73.1±7.3
*s*_15_	68.8±9.9
*s*_16_	81.7±4.8
*s*_17_	87.0±4.9
*s*_18_	87.6±7.2
**Mean (A)**	78.1±9.2

(%, mean ± standard deviation across 10 cross-validation folds) for posterior models evaluated on subjects that were completely excluded during prior training. Each row corresponds to a leave-one-subject-out experiment: a prior model was trained on all subjects except the target, then fine-tuned using only the target subject’s own data. Subjects 1–9 correspond to Dataset B (two-class MI), and Subjects 10–18 correspond to Dataset A (four-class MI reduced to two classes). Bold rows indicate dataset-wise means. Compared to seen subjects (Table 4), unseen subjects show a statistically non-significant accuracy change: a 1.1% decrease for Dataset B and a 0.8% increase for Dataset A (Wilcoxon signed-rank test: *W* = 70.0, *p* = 0.523). This demonstrates that posterior fine-tuning is robust to new subjects even without prior exposure—a key practical advantage of the BELT architecture.

### Compressor

We implemented the AutoEncoder with different architectural parameters. Denoting compression configuration as $\left(N/E_{1}/E_{2}\right)$, we systematically varied *N*, *E*_1_, and *E*_2_ across (1, 5, 9, 13), (2, 5, 10, 20), and (2, 5), respectively, yielding 32 combinations in total. For each configuration, an AutoEncoder was trained with a learning rate of 10^−3^ for 50 epochs using MSE loss and the ADAM optimizer. The comparison evaluation metric is the classification accuracy of the reconstructed signal (${\vec{x}}_{r}$) using the prior detector, with the reported accuracy drop representing the average across all *k*-folds. Importantly, the AutoEncoder training was performed on data from all subjects, since the focus here is on the impact of compression rather than subject transferability.

**Fig 11:**
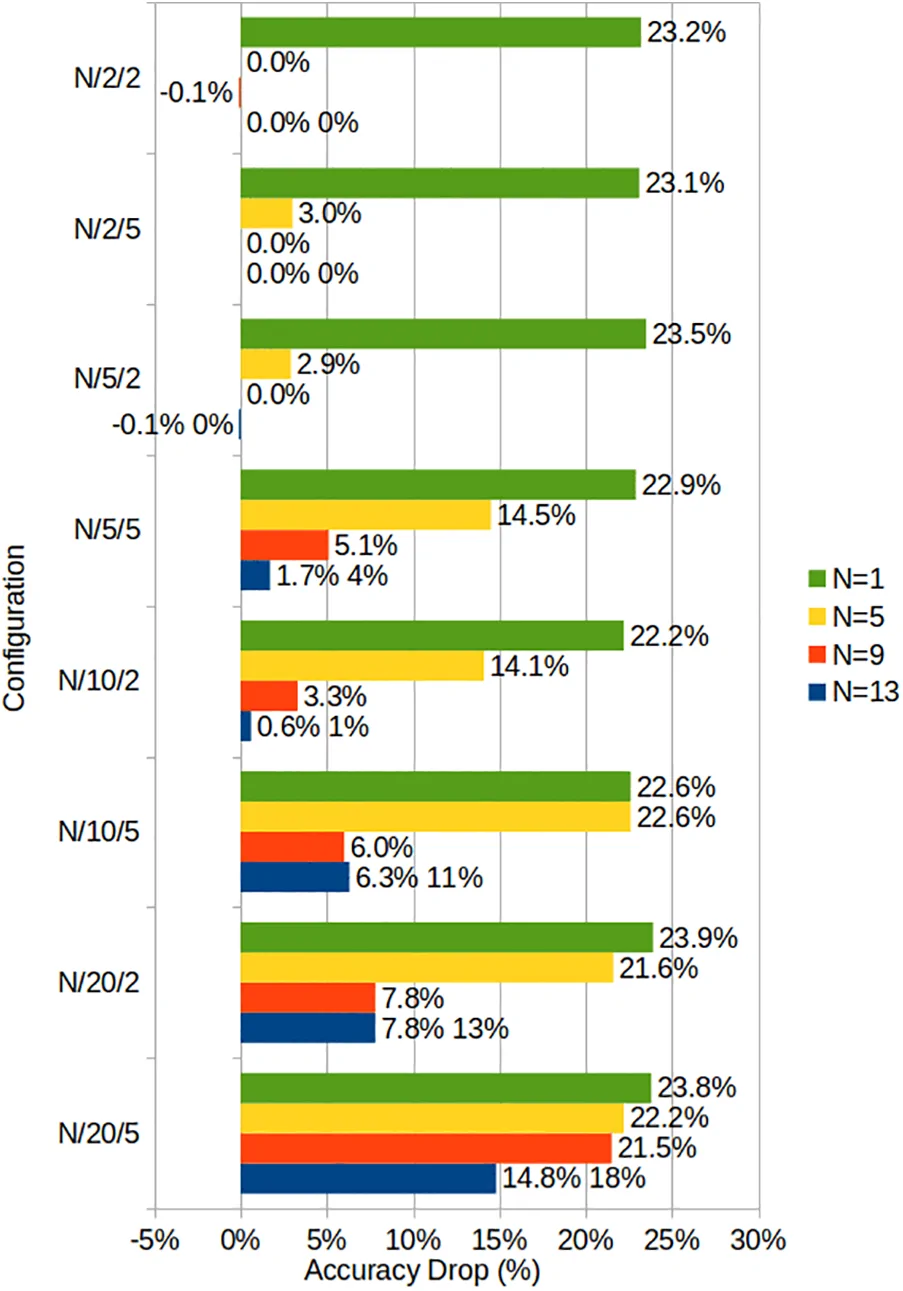
Compression configuration comparison. Positive values indicate accuracy drops (worse performance after compression), while negative values indicate accuracy improvements. An autoencoder was trained for each configuration $\left(N/E_{1}/E_{2}\right)$, and the reconstructed signal ${\vec{x}}_{r}$ was evaluated using the prior detector. The accuracy drop is defined as the change in detector performance between the original signal $\vec{x}$ and the reconstructed signal ${\vec{x}}_{r}$; all changes shown are therefore attributable to the compression–reconstruction process. Parameters $E_{1}\in 20,10,5,2$ and $E_{2}\in 5,2$ were varied, yielding eight configurations per *N* value (N∈1,5,9,13).

As shown in Fig 11, configurations with *N* = 1 consistently result in severe accuracy degradation. This outcome is expected, as collapsing all spatial information into a single mixed channel eliminates task-relevant features, forcing the optimization to prioritize reconstruction quality under the MSE penalty irrespective of classification requirements. By contrast, configurations such as (*N*/2/2) and (*N*/5/2) show minimal or no accuracy loss for *N* = 9 and *N* = 13, with occasional slight improvements observed.

Therefore, we adopt the (9/5/2) configuration (with *CR* = 3.3 and negligible accuracy gain) as the baseline for subsequent experiments. As discussed eailier, a parameter *j* was defined to control the trade-off between reconstruction fidelity and task accuracy. Based on this, we designed three model variants:

1. **Task-Unaware (TU):** *j* = 0. The model focuses solely on signal reconstruction, without regard to the classification task.
2. **Task-Only (TO):** j→∞. The model disregards reconstruction and optimizes exclusively for classification accuracy during compression.
3. **Task-Aware (TA):** 0<j<∞. The model balances reconstruction and classification objectives, with *j* determining the trade-off.

**Fig 12:**
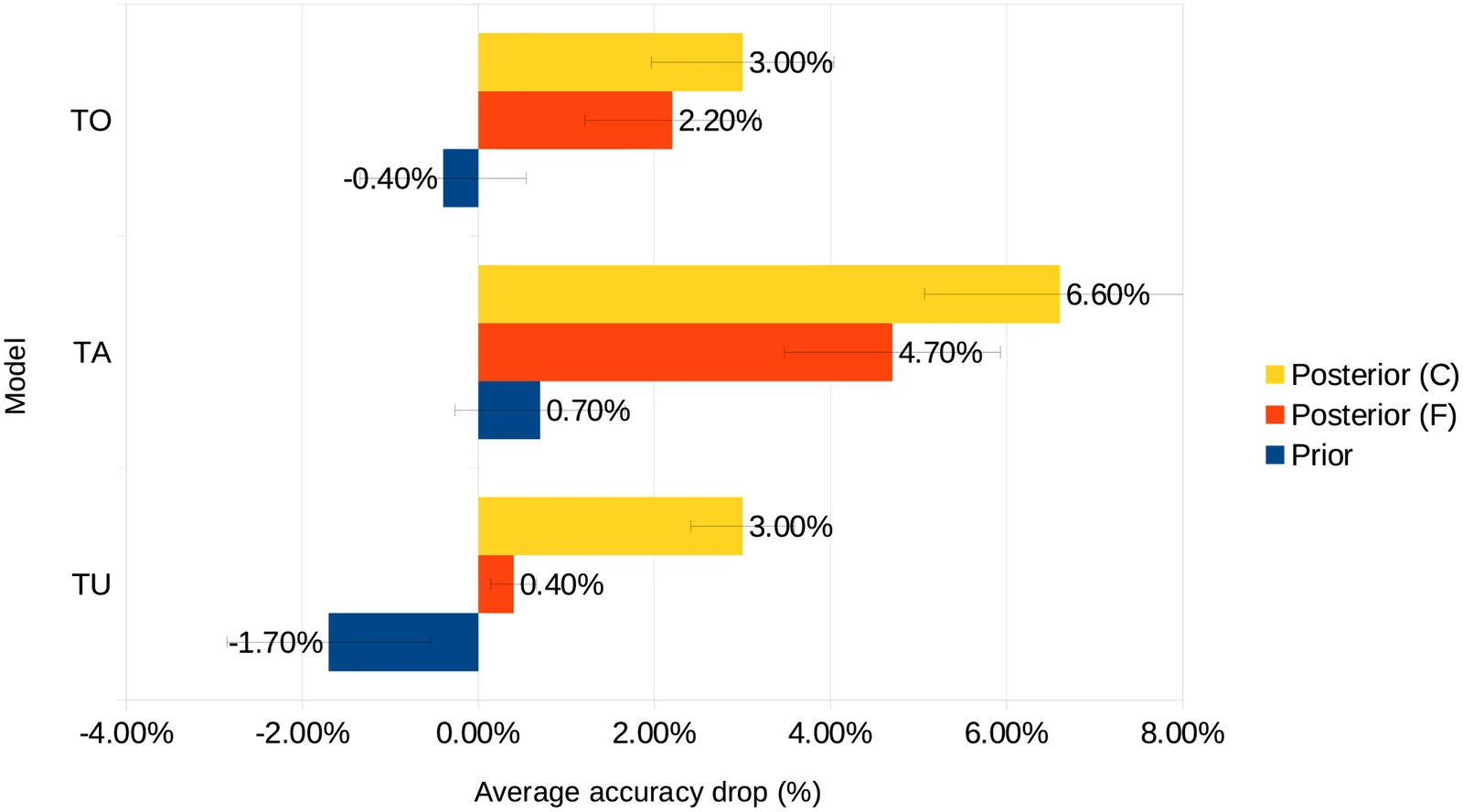
Comparison of average accuracy drop for different compression approaches. TA = Task Aware, TU = Task Unaware (MSE only). F = Full posterior training, C = Classifier-only fine-tuning. Error bars indicate standard error of the mean (SEM) across subjects. Prior models are compared to the detector-only system without compression; posterior models are compared to the posterior model without any compression and with full fine-tuning. Among posterior C models, TU achieves the smallest accuracy drop overall, with a mean reduction of only ≈3% even under classifier-only fine-tuning—a statistically significant but practically modest penalty (*W* = 2.0, $p=2.29\times 10^{-5}$).

A 10-fold experiment was conducted per subject, and average accuracy drops are reported in Fig 12, with error bars representing the standard error of the mean (SEM) across subjects. Prior and posterior detectors without compression serve as baselines.

Contrary to our initial expectations, the TU model consistently achieved the best overall performance. On prior data, TU outperformed both TO and TA, likely because the larger parameter count of TO (due to chaining the autoencoder and detector) increases overfitting risk, while the multi-objective optimisation of TA introduces additional complexity that may hinder reconstruction quality. For posterior models, TU with full fine-tuning exhibited a negligible accuracy drop relative to the no-compression baseline. Even under the more constrained classifier-only fine-tuning, TU maintained a small average drop of approximately 3%. A Wilcoxon signed-rank test confirmed that this drop is statistically significant (*W* = 2.0, $p=2.29\times 10^{-5}$), yet the absolute decrease remains modest—about 3% on average—indicating that TU compression remains viable for resource-limited deployment scenarios, such as private clouds where only classifier-side fine-tuning is feasible.

In summary, the TU model demonstrates the following:

1. Minimal degradation of prior model performance, and in some cases slight improvements.
2. An average accuracy drop of less than 0.5% when used with full posterior fine-tuning.
3. A statistically significant but practically small (≈3%) drop under classifier-only fine-tuning, making it suitable for privacy-assured but resource-constrained private cloud deployments.

These findings highlight that for our experimental setting, a simple reconstruction-based compression (TU) is more effective than task-aware or task-only alternatives, offering an attractive trade-off between accuracy and computational efficiency.

### Computational efficiency and embedded suitability

In addition to accuracy, practical deployment of BCI models depends critically on computational efficiency and hardware compatibility. To this end, we benchmarked **BELT-lite**—a constrained instantiation of the BELT architecture with only ~1.65k parameters—against **EEGNet** [47], a widely adopted lightweight CNN for EEG decoding with ~2.23k parameters. While EEGNet serves here as a strong compact baseline, it should be noted that the BELT framework is not tied to a specific feature extractor and could in principle integrate EEGNet-style blocks if desired.

#### Accuracy comparison.

Subject-level 10-fold cross-validation across all 18 subjects yielded mean accuracies of 86.1%±9.9% for BELT-lite and 88.8%±9.8% for EEGNet. A Wilcoxon signed-rank test revealed that this difference is statistically significant (*W* = 20.0, *p* = 0.0028), indicating that EEGNet maintains a small but reliable accuracy advantage over BELT-lite on the tested cohort. The absolute difference of approximately 2.7 percentage points represents the accuracy cost of BELT-lite’s extreme architectural simplicity.

#### Runtime evaluation.

Inference latency was assessed on an **OrangePi Zero** single-board computer equipped with 512 MB RAM and an ARM Cortex-A7 CPU, using TensorFlow Lite models optimized for the target architecture. For each model, we performed 100 independent measurement runs, each averaging the latency over 100 consecutive inferences (10 000 inferences total). BELT-lite achieved a mean latency of 6.75±0.010 ms per sample, while EEGNet required 8.36±0.027 ms. The difference was highly significant (Wilcoxon *W* = 0.0, $p=3.90\times 10^{-18}$), corresponding to a ~21% reduction in inference time with substantially lower variability. This speedup reflects the efficiency of BELT-lite’s FIR-style convolutions and pooling operations under resource-constrained ARM execution.

#### Embedded suitability.

The defining advantage of BELT-lite lies in its compatibility with embedded DSP pipelines. Every major computation can be reduced to FIR filtering plus a lightweight nonlinearity, enabling direct mapping to hardware primitives such as ARM Cortex-A NEON SIMD instructions. In contrast, while EEGNet is parameter-efficient, its reliance on depthwise-separable convolutions introduces more irregular memory access patterns, complicating fixed-function DSP implementation. From an embedded deployment perspective, BELT-lite thus offers a favorable trade-off: a modest (2.7 percentage point) accuracy loss is offset by significantly lower latency, lower variance, and superior structural alignment with severely resource-limited platforms.

The practical impact of this latency reduction is threefold. First, lower inference time directly translates to lower energy consumption per decision, which is critical for battery-powered wearable BCIs where every millijoule counts. Second, a 21% faster inference frees processor cycles that can be used for concurrent signal-processing tasks (e.g., artifact rejection, notch filtering) or for more frequent model updates in an online setting, improving overall system responsiveness without upgrading hardware. Third, although a 6.75 ms inference time is negligible relative to the 4 s segment length, real-world BCI pipelines often perform multiple inferences per second (e.g., sliding-window classification); the accumulated latency reduction can improve the perceived interactivity of the system. Moreover, because BELT-lite’s operations map directly onto FIR filters, the model can eventually be implemented on fixed-function DSP accelerators, which can further reduce power by an order of magnitude compared to general-purpose CPU execution — a path not available to EEGNet’s depthwise-separable convolutions.

These characteristics make BELT-lite particularly suitable for edge BCI applications where real-time performance and hardware simplicity are paramount.

## Conclusion

Brain–computer interfaces have yet to achieve adoption levels comparable to conventional human–computer interaction devices. We have argued that this gap stems not from processing limitations, but from architectural choices that couple systems too tightly to specific applications, overlook portability, and inadequately address user adaptation, cloud dependence, and privacy.

We proposed BELT—a modular Bayesian Edge–Cloud architecture designed to accommodate deep learning’s computational demands while handling EEG signal variability. By separating general-purpose communication from application-specific logic, BELT enables reusable components with task-specific customization via the Network Tuning Block.

BELT-lite demonstrated practical feasibility: with only 1.65k parameters and FIR-style operations, it matched EEGNet’s accuracy closely while reducing inference latency by 21% on ARM hardware—a highly significant advantage for embedded deployment. Moreover, the low parameter count of BELT-lite also reduces the cloud compute required for subject-specific fine-tuning, which shortens calibration turnaround and lowers cost in pay-as-you-go cloud environments — an important practical advantage for scalable BCI services. Besides, although EEGNet maintained a small (2.7%) accuracy advantage, BELT-lite’s DSP-friendly design makes it particularly suitable for mass-produced devices.

The Bayesian prior–posterior formulation proved essential: posterior fine-tuning significantly improved accuracy across all subjects. Using the augmented prior ($a_{aug}=1$), the mean accuracy increased from 77.2% to 87.9% on Dataset B (+10.7 percentage points) and from 56.7% to 80.6% on Dataset A (+23.9 percentage points). Freezing LTI filters during fine-tuning yielded performance indistinguishable from full fine-tuning, while classifier-only fine-tuning incurred a modest but statistically significant penalty of 2–5%—acceptable for resource-limited cloud scenarios.

Signal compression achieved 3.3x bandwidth reduction with negligible accuracy loss in prior models and only a practically small (≈3%) drop under classifier-only fine-tuning, enabling privacy-preserving edge–cloud communication through latent representations rather than raw neural data.

Together, these findings establish BELT as a principled foundation for scalable, portable, and privacy-aware BCI systems—a pathway toward broader adoption. Future work will focus on hardware-in-the-loop implementations and adaptive privacy mechanisms to accelerate the transition from laboratory demonstrations to everyday applications.
