# Supplementary material for: Improved motor imagery BCI performance via task-unaware compression in the BELT Bayesian Edge-Cloud architecture
Source: PLoS One. 2026 Aug 21;21(8):e0354976. doi: 10.1371/journal.pone.0354976 (PMC13497277; doi:10.1371/journal.pone.0354976)
Supplement: Supplementary file 1 [file pone.0354976.s002.pdf]

Response to Reviewers

Bayesian Edge-Cloud BCI Architecture: Lightweight Classification and Task-aware Compression

PLOS ONE

February 20, 2026

Dear Editor,

Thank you for the opportunity to resubmit a revised version of our manuscript. We also appreciate the extended deadline, which allowed us to thoroughly address the reviewers' comments.

In response to the valuable feedback received, we have substantially strengthened the study. Most notably, we have expanded our dataset by including BCI Competition IV-2a, effectively doubling the subject pool from 9 to 18. This addition required re-running all experiments and updating all statistical analyses accordingly. The revised manuscript now includes comprehensive statistical testing (Friedman, Nemenyi, and Wilcoxon tests) throughout, as recommended by the reviewers.

We are uploading the following files:

1. A point-by-point response to all reviewer comments (below)
2. A marked-up manuscript showing all changes, generated using `latexdiff` ("Revised Manuscript with Track Changes")
3. A clean manuscript without highlighting ("Manuscript")
4. New set of figures ("figs.zip")

We believe the revisions have substantially improved the manuscript and hope it is now suitable for publication in PLOS ONE.

Sincerely,
Hamid Soltanian-Zadeh
(on behalf of all authors)

Reviewer 1

Reviewer #1, Concern #1: Line 9: The authors claim that BCI systems remain neither cost-effective nor mass-producible. However, this is not the case, since there are many consumer-grade BCI systems. This review mentions 5 of these systems: Sabio, J., Williams, N. S., McArthur, G. M., & Badcock, N. A. (2024). A scoping review on the use of consumer-grade EEG devices for research. PLoS One, 19(3), e0291186. The authors should rethink the line of argumentation.

Author response: Thank you for this important observation. You are correct—there are indeed several consumer-grade EEG systems available, as highlighted in Sabio et al. (2024).

Author action: We have revised the Introduction accordingly. The argumentation no longer claims that BCI systems are not cost-effective or mass-producible at the hardware level. Instead, we now frame the discussion around adoption levels relative to widely used HCI tools (e.g., mouse and keyboard), which remains an open challenge despite hardware availability. The revised Introduction is shorter and addresses this comment, among others.

Reviewer #1, Concern #2: Lines 18-22: The authors claim that consumer-grade BCI tools remain scarce and point to some barriers for that, specifically the biological variability, ethical and privacy issues, and the technical constraints of real-time portable implementations. As I already mentioned, there are at least 5 consumer-grade BCI systems. If this is too little or too much is a matter of personal opinion. However, I don't see that the presented barriers are what actually defines the number of available options. This seems more related to economical dynamics of a niche market. The market of mobile operating systems, for instance, is represented by a duopoly formed by Android and iOS systems, but this is much more related to economy than to science. A similar situation occurs in BCI systems. As showed in the review I mentioned (Sabio, 2024), Emotiv leads the market by a wide gap. According to the homepage of their website (https://www.emotiv.com/), they sold more than 45 thousand devices. People tend to buy something that has already been proven to work, which makes it harder for new devices to join the market. Also, this review shows that EPOC usage is wide-spread around the world: Williams, N. S., McArthur, G. M., & Badcock, N. A. (2020). 10 years of EPOC: A scoping review of Emotiv's portable EEG device. BioRxiv, 2020-07. Based on that, the authors should avoid stating that BCI tools remain scarce, and that this scarcity is caused by the mentioned barriers. This line of reasoning is not necessary for the goals of the study.

Author response: Thank you for this thoughtful comment. You raise a valid point—consumer-grade BCI systems do exist (e.g., Emotiv EPOC), and their market presence is shaped more by economic dynamics than by the scientific barriers we initially listed.

Author action: We have revised the relevant paragraph (now lines 15–21) to: (i) acknowledge the existence of consumer-grade systems, citing Sabio et al. (2024) and Williams et al. (2020); (ii) remove the causal claim that the listed barriers explain the limited number of available devices; and (iii) reframe the discussion to focus on adoption challenges rather than hardware scarcity, aligning with the paper's goals.

Reviewer #1, Concern #3: Line 33: The authors mention a re-implementation

issue. Actually this is an issue of lack of code availability from other studies. Reference 7 re-implements the code for a technique because the work they are using did not share the code to implement the technique. It's not because of difficulty to generalize the solutions. Rewrite this part.

Author response: Thank you for this clarification. You are correct—the issue is not about difficulty in generalising solutions but rather the lack of code availability from prior studies.

Author action: We have removed this example from the Introduction, along with the mention of interdisciplinary problems, to streamline the narrative and avoid misinterpretation. The revised text now focuses solely on the core argument without this potentially misleading example. (Now lines 22-34)

Reviewer #1, Concern #4: Line 34: The authors state that the re-implementation issue is described as a need for interdisciplinary teams. I think the authors mean that the need of re-implementation demands interdisciplinary teams. But this was already said. Explain better.

Author response: Thank you for pointing this out. We agree the phrasing was unclear.

Author action: As noted in our response to Comment 3, we have removed this example entirely from the Introduction. The revised text no longer mentions the re-implementation issue or the need for interdisciplinary teams in this context, allowing the narrative to flow more clearly toward the paper's core contributions.

Reviewer #1, Concern #5: Line 59: Removing the word "still", the phrase would make much more sense.

Author response: Thank you for this suggestion.

Author action: We have paraphrased the introduction heavily to be shorter. The still world is removed.

Reviewer #1, Concern #6: Line 60: When talking about non-stationarity, the authors should avoid mentioning the reference 13, which doesn't use this term. They use the term day-to-day variability. Instead the authors should cite reference 5, which mentions stationary and non-stationary signals in BCI Challenges. Actually, this is the only reference that I found, among the references cited in sentences in the text that talk about stationarity, that actually uses the word stationary when talking about signals. I think stationary usually has a different meaning in the context of signal analysis, that is specific to characteristics of a single time series that are constant along time, and not as a generic term for variability.

Author response: Thank you for this important clarification. You are correct - non-stationarity and variability are distinct concepts.

Author action: Following your suggestion, we have: (i) removed the term

"non-stationary" from the paragraph; (ii) replaced it with "inter- and intra-subject variability," which is more precise and commonly used in the BCI literature; and (iii) updated the citations to accurately support this revised framing (now referencing the appropriate source). This change also aligns with Comment 8. The revised paragraph now presents a more accurate and community-familiar description of signal variability. The paragraph is now at lines 35-42

Reviewer #1, Concern #7: Lines 61-62: This phrase is very confusing. I don't see why this decoding, that can be made with any device, requires substantial processing power. I think the authors want to say that portability is necessary, but so as is processing power. This can be said, but without the word consequently, since I don't see the relation between portability/processing power with user-adaptation/non-stationarity.

Author response: Thank you for this observation. We agree the original phrasing was confusing.

Author action: We have removed the problematic sentence. The connection between processing power requirements and our approach is now established through a discussion of deep learning benefits in BCI, which naturally motivates the need for adequate computational resources. The revised text (lines 43-45) presents this relationship more clearly without the misleading "consequently" link.

Reviewer #1, Concern #8: Line 64: Again, I recommend avoid the use of the term non-stationarity as a generic term to mean variability. Stationary is usually evaluated along a single time series. It's uncommon to use this term when talking about the day-to-day variability. Special attention should be made for reference 15, where they do not use the term stationarity. Instead they talk about inter-subject variability. This source of variability is even less associated with stationarity, since it has no relation with time, so the term non-stationarity is clearly misused here. It's best to avoid the use of the term non-stationarity throughout the paper, and use terms related to variability, and to the type of variability accordingly.

Author response: Thank you for this important clarification. You are absolutely right—"non-stationarity" is a specific signal processing term that should not be used interchangeably with "variability," particularly when referring to inter-subject differences, which have no temporal component.

Author action: Following your recommendation, we have: (i) removed the term "non-stationary" throughout the introduction; (ii) replaced it with precise terms: "inter-subject variability" and "intra-subject variability" as appropriate; and (iii) updated the relevant citations to accurately support this revised framing. This change has been applied consistently across the paper, including in corresponding lines and elsewhere (except for one occurrence in the literature review section). It also aligns with our response to Comment 6. Now lines 35-42

Reviewer #1, Concern #9: Line 72: The authors should mention here, why Edge-Cloud architectures are more viable than cloud-based systems. I see they start to define Edge-Cloud at line 200, but a brief explanation here could help the understanding of the readers.

Author response: Thank you for this helpful suggestion.

Author action: Following your recommendation, we have added a brief explanation of Edge-Cloud architecture in Lines 51-54.

Reviewer #1, Concern #10: Line 75: As I mentioned while commenting lines 9 and 18, the argument that BCI systems are not practical needs to be revisited. BCI systems are practical and are being used world-wide. If the authors don't make clear the advantage of Edge-Cloud architecture, the readers will have difficulties to understand the problem, especially when they do not agree with mentioned impracticability. So far in the reading, I don't see the advantage of the proposed architecture. This can make the reader stop the reading at this point.

Author response: Thank you for this important observation. You are correct—BCI systems are indeed practical and commercially available.

Author action: Following your suggestion, we have: (i) removed the term "impracticality of BCI" entirely from the manuscript; (ii) reframed the discussion to focus on how Deep Learning addresses variability challenges; (iii) clarified that Cloud and Edge-Cloud architectures enable lightweight, low-cost end-devices while supporting DL capabilities; and (iv) added a nuanced comparison: cloud computing introduces new challenges (e.g., latency, privacy), which Edge-Cloud architectures can help mitigate. This is done through lines 43-56.

Reviewer #1, Concern #11: Line 95: This brings the idea that BELT-lite is practical and BELT is impractical. And the whole point so far it to make things practical. So, BELT is not the desired framework, but BELT-lite. If that's not the case, rewrite this part.

Author response: Thank you for this insightful observation. You are correct—the original phrasing could imply that BELT itself is impractical, which contradicts our overall goal.

Author action: We have revised the text accordingly: (i) changed "we introduce a practical realization of this framework" to "we introduce a lightweight deep-learning model within this framework" (Line 77); (ii) removed the term "practical" from this context entirely; and (iii) shortened the paragraph for conciseness and clarity. These revisions clarify that BELT-lite is a lightweight variant within the same framework, not a more practical alternative to an impractical system.

Reviewer #1, Concern #12: Line 96: Actually, the arguments presented were about deep learning. I understand that deep learning is a subfield of neural networks, but you can't discuss something specific and then say, without a reason, that you are going to use something more generic, based on the presented arguments. I see that your model is actually based on deep learning, so it's better to keep consistency and say that BELT-lite model is built using deep learning.

Author response: Thank you for this consistency check. You are absolutely right—our arguments focused on deep learning, so it is appropriate to maintain that specificity.

Author action: We have revised the text to state that "BELT-lite is a lightweight deep-learning model" (Line 77) rather than referring to neural networks generically. This change has been applied in line 96 and ensures consistency throughout the paragraph.

Reviewer #1, Concern #13: Line 101: So far in the reading it's still not clear to me which are the interdisciplinary problems and how they can be solved by architecture adaptation.

Author response: Thank you for this observation.

Author action: Following our broader reorganization of the Introduction, the mention of interdisciplinary problems has been removed entirely. The revised text no longer includes this unclear reference, and we have streamlined the narrative to focus directly on the architectural motivations for our work. The current version now presents a clearer and more focused argument without the ambiguity you identified.

Reviewer #1, Concern #14: Line 112: Here I finally see the advantages of Edge-Cloud. This should happen earlier.

Author response: Thank you for this structural feedback. You are right—introducing the advantages of Edge-Cloud earlier improves readability.

Author action: Following your suggestion, we have moved the first explanation of Edge-Cloud benefits to an earlier point in the manuscript, Lines 51-54.

Reviewer #1, Concern #15: Line 122: This contradicts the idea that BCIs are not practical.

Author response: Thank you for catching this inconsistency. You are correct—the original phrasing contradicted our revised stance on BCI practicality.

Author action: Following the broader reorganization of the Introduction (as detailed in our responses to Comments 1, 2, and 10), we have removed all claims suggesting BCIs are impractical. The argumentation now consistently frames BCIs as practical systems facing adoption challenges rather than fundamental feasibility issues.

Reviewer #1, Concern #16: Line 125: Here you explicitly say that BCIs are practical.

Author response: Thank you for highlighting this contradiction. You are absolutely right.

Author action: The revised paragraph now presents a coherent narrative via Lines 104 and 105.

Reviewer #1, Concern #17: Line 129: I disagree with the comment "This definition is more emphasizing the communicational side of BCI rather than the direct usage or application of a BCI system". The usage or application of a BCI system, inevitably requires a communication between the brain and an external device. Different applications have different adherence to the term communication if you think about person to person communication. But, for being a BCI, they all enable a communication between the brain and a device, according to Vidal's definition. For instance, both a system to send a message to somebody based on brain activity and a system that emits a sound alarm when detecting driver drowsiness are BCIs.

Author response: Thank you for this thoughtful comment. You are correct—Vidal's definition inherently includes communication, and our original phrasing misrepresented this.

Author action: We have removed the statement that the definition emphasizes communication over application, as this distinction was unnecessary and potentially misleading. This revision is part of the broader reorganization of the section addressing Comments 17 and 18. Please checkout the paragraph in Lines 112-130.

Reviewer #1, Concern #18: Lines 133-144: This paragraph can be heavily summarized. BCI is a subfield of HCI, but this does not bring any conclusions. EEG-based BCIs are not wearable in nature, since many of these systems are not wearable. The important point of this paragraph is the importance of portability, which is evident. Especially when talking about consumer-grade BCIs. In that case, the bigger the portability, the better.

Author response: Thank you for these thoughtful comments. We agree with both points.

Author action: Following your suggestion, we have: (i) merged and heavily summarized this paragraph and the previous one into one paragraph (Lines 112-130); (ii) removed the incorrect note about BCIs being "wearable in nature"; (iii) centered the discussion on portability as a key design consideration for consumer-grade BCIs; and (iv) added a brief explanation of how architectural approaches (like our BELT framework) can address portability challenges while maintaining performance.

Reviewer #1, Concern #19: Line 149: I still don't get what the authors define as the communicational aspect of BCI. I think the authors should make it more clear.

Author response: Thank you for pressing for clarity. We agree the original wording was too vague.

Author action: To address this, we have introduced a more precise term: "reusable, application-agnostic telecommunicational perspective on BCI." (Lines 136 and 137). This phrase captures the idea that, from a telecommunications viewpoint, the BCI system can be designed independently of the specific end-application—much like a demodulator is separate from the application that uses the decoded information. We have defined and explained this concept more clearly in the revised text, where we contrast it with the application-coupled perspective of modern BCIs.

Reviewer #1, Concern #20: Lines 154-160: The authors say that exploring portability demands large-scale data collection, but I don't see the connection. The validation of generalizability can occur with the same dataset size requirements for any validation. The authors also say that can achieving portability at scale is only feasible through mass production, particularly given the time and cost associated with acquiring EEG-BCI data in controlled research settings. However, this is not the case. The same can happen by conducting many data collections with the production of a few portable BCI units, in a multi-centric study. The authors should rewrite these parts, talking about the importance of portability, large-scale data collection and mass-production, but without stating that these things are always co-dependent. At line 169 the authors say that DL requires large amounts of training data. This makes more sense. So the authors should say the requirements for a lot of data is caused by dealing with DL, rather than caused by portability.

Author response: Thank you for this observation. You are right—the connection between portability and large-scale data collection was unclear and not well justified.

Author action: Since the following paragraph introduces deep learning in BCI, which provides a stronger motivation for our work, we have simply removed the problematic sentences (formerly lines 154–160). The revised text now flows directly into the discussion of deep learning without this unconvincing intermediate argument.

Reviewer #1, Concern #21: Line 193: This adaptation is not necessarily performed on the same device, as the authors suggest on the next paragraph a solution that involves it not being performed on the same device. Rewrite.

Author response: Thank you for catching this inconsistency. You are absolutely right—the original text stated that adaptation must occur on the device,

which directly contradicted our proposed Edge-Cloud solution where adaptation happens elsewhere.

Author action: We added "when the BCI system is computationally limited to the standalone portable user-side device, ..." at lines 173 and 174 and modified the paragraph.

Reviewer #1, Concern #22: Equation 1: For better understanding of the equation, the authors should denote what is N.

Author response: Thank you for this suggestion.

Author action: We have added a definition for N directly at line 223, specifying that it represents the total number of segments in the prior dataset. This clarification improves readability for readers unfamiliar with the notation.

Reviewer #1, Concern #23: Line 242: Cite some literature mentioning this improvement, as well as listing some techniques for data augmentation with EEG data.

Author response: Thank you for this suggestion.

Author action: Following your recommendation, we have added citations to the literature and included examples of EEG data augmentation techniques (Lines 228-235). Specifically, we now reference: (i) a GAN-based approach (similar to our method); (ii) a simple signal manipulation technique (CutCat); and (iii) a method adapted from speech processing (SpecAugment). These examples illustrate the range of available augmentation strategies and support our claim that augmentation improves classification performance.

Reviewer #1, Concern #24: Equation 3: From my understanding, probability notation is always between parentheses, not brackets. Let me know if I'm wrong.

Author response: Thank you for this observation. You are correct—probability notation conventionally uses parentheses.

Author action: We have updated all instances of $Pr[...]$ to $Pr(...)$ throughout the manuscript accordingly.

Reviewer #1, Concern #25: Equation 4: θ_c, θ_d and c with an arrow on top are not defined.

Author response: Thank you for pointing this out.

Author action: We have rewritten the paragraph to clearly define all symbols, including θ_c (Line 225), θ_d (Line 225), $\vec{c}$ (equation 4), and $\vec{x}_r$ (equation 5), before their first use in the equations.

Reviewer #1, Concern #26: Equations 5 and 6 are not mentioned in the text. The text doesn't make clear how Lr and Lt are calculated. These equations imply that the calculations involve a Xr, but this variable is defined only in line 274, when talking about step 6. Perhaps these equations should be mover to be near the paragraph talking about step 6. All these problems bring difficulty to understand the equations.

Author response: Thank you for this observation.

Author action: We have revised the presentation of Equations 5 (now 7) and 6 (now 8). (Lines 260-262 explains them) Specifically, we replaced k with j to avoid confusion with the k-fold notation used later in the paper. Additionally, we corrected the definition of L_r to use mean squared error (MSE), which is the appropriate formulation for continuous signals. The equations are now introduced and explained in the text before they appear.

Reviewer #1, Concern #27: Line 276: Combing this paragraph with the previous one would make it more clear that this line is talking about equation 7.

Author response: Thank you for this suggestion.

Author action: We have combined the paragraphs as recommended to improve clarity and show the connection to Equation 9. (Lines 278-283)

Reviewer #1, Concern #28: Line 317: The citation and the position of Table 1 should be moved to the beginning of the Architecture section, just before the first usage of mathematical notations. This can help readers understand the equations. For readers unfamiliar with probabilistic mathematical notation, I recommend to add Pr (A — B), described as the probability of event A given event B.

Author response: Thank you for this suggestion.

Author action: We have moved Table 1 to the beginning of the Architecture section as recommended. Additionally, we have added an entry for $Pr(A|B)$ in the table, describing it as the probability of event A given event B, and added a line indicating that the arrow symbol denotes vectorized signals or data.

Reviewer #1, Concern #29: Line 366: This is the first time you are talking about DSP, but this abbreviation is not defined anywhere. I assume this is Digital Signal Processing. The authors should define it here.

Author response: Thank you for pointing this out.

Author action: We have modified the sentence to include the full phrase "Digital Signal Processing (DSP)" at its first occurrence.

Reviewer #1, Concern #30: Lines 413-416: This paragraph confuses the

reader to understand which interpretation is being talked about. Also, what promotes the consistency with the rest of the detector?

Author response: Thank you for this observation. We agree that the final sentence of this paragraph was unclear.

Author action: We have removed it to eliminate confusion, as the preceding sentences already provide sufficient justification for the two-layer classifier design from both efficiency and theoretical perspectives. Revised in Lines 415 and 416.

Reviewer #1, Concern #31: Lines 418-557: All these parts are detailing how everything was executed, so should be moved to the Material and Methods section.

Author response: Thank you.

Author action: The mentioned parts have been moved to the Materials and Methods section. Additionally, as suggested in Comment 47, the corresponding compressor implementation details have also been relocated to ensure consistency. The cGAN implementation details (Comment 37) have been moved there as well. All of them are under related subsection titles.

Reviewer #1, Concern #32: Line 435: How artifacts and acquisition errors were detected? All the details about this preprocessing should be mentioned, as applied filters, for example.

Author response: Thank you for this important observation.

Author action: We have expanded the dataset description to clarify how artifacts and acquisition errors were detected. Specifically, we now describe that trials were originally flagged by the dataset providers based on visual inspection of muscle artifacts, eye movements, and technical disturbances. We adhered to these provided rejection flags and applied no additional artifact rejection, ensuring consistency with prior benchmarks. The revised paragraph now provides complete transparency regarding our preprocessing approach. (Lines 446-452)

Reviewer #1, Concern #33: Line 447: Put the link for the repository in the text.

Author response: Thank you for the suggestion.

Author action: We have added the repository link prominently at the beginning of the Implementation section to ensure easy access for readers interested in reproducing our work.

Reviewer #1, Concern #34: Line 484: It's better to rewrite this, avoiding narrative citations, that is, when the citation is part of the text.

Author response: Thank you for this observation.

Author action: We have revised the sentence to use a parenthetical citation, improving readability. The updated sentence now reads: "As shown in our prior work [13], this log-power structure enables a two-phase training procedure..." (Line 520)

Reviewer #1, Concern #35: Line 499: Cite the findings

Author response: Thank you.

Author action: Citations have been added for the beta and mu rhythm findings as requested. Line 534.

Reviewer #1, Concern #36: Line 542: The parameter k_{aug} can easily be confused with k. Use a different nomenclature for parameters. I recommend using the letter a instead of k_{aug}, to indicate the level of augmentation.

Author response: Thank you for this helpful observation. We agree that using k_{aug} could cause confusion with the trade-off parameter k.

Author action: Following your suggestion, we have renamed the augmentation parameter. However, to maintain clarity regarding its role, we chose a_{aug} rather than a standalone a, as the subscript explicitly indicates its association with augmentation. This change has been implemented throughout the manuscript. Also, since k has been used for k-fold, we used j instead.

Reviewer #1, Concern #37: 544: Which augmentation techniques were used?

Author response: Thank you for this important observation. We needed to add details.

Author action: We have significantly expanded the description of our data augmentation approach. Following your suggestion, we moved the detailed GAN architecture and training specifications to the Materials and Methods section for better structural clarity under "Data Augmentation" title, Page 15.

Reviewer #1, Concern #38: Tables 3 - 7: The authors are confusing k and k_{aug}. The legend says k_{aug} but the column headers say k.

Author response: Thank you for pointing out this inconsistency.

Author action: We have corrected the column headers in Tables 3 - 7 to match the legend. Following your earlier suggestion (Comment 36), we now use a_{aug} consistently throughout the manuscript - both in the text and in all table headers. The notation is now uniform across all tables and the main text.

Reviewer #1, Concern #39: Line 564: I don't see what is the value of k, that is, the number of folds. How many models were used for each subject in

each fold? From my understanding, the model is only one: BELT-lite. So I don't understand how to pick the best-performing models.

Author response: Thank you for your insightful comment.

Author action: We have clarified the following in the revised manuscript: (i) 10-fold cross-validation was used for each subject; (ii) within each fold, five BELT-lite models were trained with $a_{aug} \in \{0, 1, 2, 3, 4\}$; (iii) the model with the highest validation accuracy was selected for testing; (iv) the a^* column reports the test accuracy when the optimal a_{aug} is chosen per fold (oracle upper bound); and (v) a Friedman test across all six conditions was significant ($\chi^2(5) = 47.17$, $p = 5.2 \times 10^{-9}$), with Nemenyi post-hoc tests showing that only a^* differs significantly from other levels. These results show that fixed augmentation does not reliably improve performance, but subject-specific tuning (oracle) does, highlighting the importance of personalisation in BCI.

Reviewer #1, Concern #40: Lines 601-603: The authors should avoid to draw conclusions based on differences of mean accuracy, as they can be caused by chance. To know if a difference in accuracy is big enough to be considered the authors should apply some repeated measures statistical testing, like the Friedman test, for each table, to see if k_{aug} influence is significant. If it is, the authors should apply post-hoc tests, to find out which pairs of k_{aug} are significantly different.

For examples, the authors should check the following studies, which were cited by reference 33: Bang, J. S., Lee, M. H., Fazli, S., Guan, C., & Lee, S. W. (2021). Spatio-spectral feature representation for motor imagery classification using convolutional neural networks. IEEE Transactions on Neural Networks and Learning Systems, 33(7), 3038-3049; Dai, G., Zhou, J., Huang, J., & Wang, N. (2020). HS-CNN: a CNN with hybrid convolution scale for EEG motor imagery classification. Journal of neural engineering, 17(1), 016025.

When doing that, the authors may find troubles regarding the small sample size of the study. The authors should increase the number of used datasets. One easy way to do that, is to include the Graz data set A, instead of using only the data set B. Here is the reference for dataset A: ...

Author response: Thank you for this important methodological suggestion.

Author action: Following your recommendation, we have: (i) performed repeated measures statistical testing, Friedman tests with Nemenyi post-hoc for all relevant comparisons with more than 2 conditions and Wilcoxon for 2 conditions; (ii) expanded our dataset by including the Graz dataset A (Brunner et al., 2008), increasing our subject pool from 9 to 18; and (iii) updated all analyses accordingly. These changes are reflected throughout the revised manuscript and results sections.

Reviewer #1, Concern #41: Fig 4: I ask for an additional figure where each bar represents a k_{aug} level, and the error bars represent the standard error from

mean across subjects. This would be a good way to show differences promoted by k_{aug}.

Author response: Thank you for the suggestion.

Author action: We have added a new figure (Figure 5) showing mean accuracies across all 18 subjects for $a_{aug} = 0$, 1, and a^*, with SEM error bars. The figure visually confirms our statistical findings—no significant difference between fixed augmentation levels, but clear improvement with oracle selection. The figure and a brief description have been included in the revised manuscript.

Reviewer #1, Concern #42: Lines 617-623: If comparing the mean accuracy is not enough information to make conclusions, a worse situation is to compare the means of single subjects. I reinforce the need to perform statistical inference.

Author response: Thank you for reinforcing the need for statistical inference. We have removed the subjective single-subject comparisons. In their place, we conducted a Friedman test across four conditions (prior $a_{aug} = 0$, prior a^*, posterior $a_{aug} = 0$, posterior a^*), which was highly significant ($\chi^2(3) = 54.00$, $p = 1.12 \times 10^{-11}$). A Nemenyi post-hoc heatmap formally demonstrates that both posterior conditions significantly outperform the non-augmented prior, while posterior ($a_{aug} = 0$) does not significantly outperform the oracle-augmented prior. Interestingly, the difference between the two posterior conditions, which appeared suggestive in a focused three-condition test, does not survive the more conservative multiple-comparison correction when prior models are included.

Author action: We now report the four-condition test as our primary analysis and discuss this nuance in the text (Lines 718-746) and with new Figure 6

Reviewer #1, Concern #43: Line 655: No. The limited size makes the insight less significant. That happened only in two of the nine subjects, so could be happening by chance.

Author response: Thank you for this important observation. You are correct—with only nine subjects and effects observed in just two individuals, drawing conclusions would be premature. Following your recommendation, we have: (i) expanded our dataset by including the Graz dataset A, increasing our subject pool to 18; (ii) applied statistical inference rather than relying on individual subject observations; and (iii) revised our analysis in Figures 5 and 7 to show aggregate results with proper error bars (SEM) and statistical testing in a new Figure 6. The updated analysis reveals that while full fine-tuning significantly outperforms classifier-only optimization at the group level ($p < 0.05$), the absolute difference is small (2–5%), supporting our original conclusion about efficiency–accuracy trade-offs but now with proper statistical grounding.

Author action: The revised text no longer references individual subject patterns. Figures 5 and 7 are now showing SEM and Fig 6 and 7 are added.

Reviewer #1, Concern #44: Fig 5: Instead of comparing with the best performing subject, p-values comparing each method is a better way to should, both what is different and what is not.

Author response: Thank you for this excellent suggestion.

Author action: We have completely redesigned the Figure 8 (former 5) to replace the "best-performing subject" comparison with proper statistical comparisons between methods as described in our response to comment 43. The figure now shows mean accuracy across all 18 subjects for both datasets (IV-2a and IV-2b) under each fine-tuning condition, with SEM error bars.

Reviewer #1, Concern #45: Line 690: Why the accuracies for subject 4 are the same on Table 8, but t It's strange that

Author response: Thank you for this observation. We agree that the original caption was unclear regarding the purpose of including Subject 4.

Author action: We have revised the caption to explicitly explain the experimental design. This revision clarifies that Subject 4 serves as a control condition to ensure that all prior models are equivalently trained. The near-identical accuracies for Subject 4 across rows (as noted by the reviewer) confirm that the observed variation in new-subject performance reflects genuine differences in how well each excluded subject is represented by the prior model, rather than inconsistencies in model training. The reason that the line for s4 has the same column values is that s4 is both the new unseen subject and fixed control subject for that model.

Reviewer #1, Concern #46: Line 692: Looking at Table 8, I don't see that the exceptions are s3 and s4. Only s4. But this is always the case.

Author response: Thank you for the correction. You are right—only s4 showed exceptional behavior, not s3. More importantly, we agree that focusing on individual subjects is not statistically sound.

Author action: Following your previous comments, we have removed all discussion of s3 and s4 and replaced it with a Wilcoxon signed-rank test across all 18 subjects. The test confirms a significant overall accuracy drop ($p = 0.027$), providing rigorous group-level evidence rather than relying on anecdotal outliers. The manuscript has been updated accordingly at Lines 812-816 for prior and Lines 822-828 for posterior.

Reviewer #1, Concern #47: Lines 706-749: All these details should me moved to the Materials and Methods section.

Author response: Thank you.

Author action: As noted in our response to Comment 31, the specified lines have been moved to the Materials and Methods section. The manuscript has

been updated accordingly.

Reviewer #1, Concern #48: Fig 8: Make clear at the legend that the accuracy drop is caused by compression.

Author response: Thank you.

Author action: We have updated the caption to clarify that all accuracy drops shown are attributable to the compression–reconstruction process. The revised caption now includes "Accuracy drop caused by compression across different configurations.". This is now Fig 12.

Reviewer #1, Concern #49: Line 758: The authors should choose different parameters names, as k is already the parameter for the number of folds.

Author response: Thank you for pointing this out.

Author action: We have renamed the parameter from k to j throughout the manuscript to avoid confusion with the k-fold notation. The manuscript has been updated accordingly.

Reviewer #1, Concern #50: Fig 9: This figure clearly could be presented with error bars, to show the variability across folds. Look in all figures for ways to present error bars.

Author response: Thank you.

Author action: We have added SEM error bars to the Figure 13 to show the variability, and reviewed all other figures for consistency. The manuscript has been updated accordingly.

Reviewer #1, Concern #51: Lines 798-830: The informations regarding how the tests were performed should be moved to the Materials and Methods section.

Author response: Thank you for this observation.

Author action: Following your suggestion, we have moved all method-based descriptions from the Results section to the Materials and Methods section under "Comparative Baseline Model: EEGNet" title.

Reviewer #1, Concern #52: Lines 806-812: Here is the single time the authors perform a statistical test. The same should be applied for all analysis.

Author response: Thank you for this important methodological guidance. You are correct—statistical testing should be applied consistently throughout the manuscript.

Author action: This has now been done for all relevant comparisons, as discussed in our responses to Comments 40, 42, 43, and 53. The manuscript now

includes consistent statistical testing throughout.

Reviewer #1, Concern #53: Lines 813-820: Perform statistical analysis here too.
Author response: Thank you for pointing out this omission.
Author action: We have now performed and reported a Wilcoxon signed-rank test for the runtime evaluation data, and the results are included in the revised manuscript.

Reviewer 2

Reviewer #2, Concern #1: In the abstract, the authors state that BELT-lite is faster than EEGNet and that several subjects achieve accuracy above 90%, but no concrete numerical values are provided. For clarity and scientific completeness, the authors should report the actual processing time (in milliseconds or seconds) for BELT-lite versus EEGNet, as well as explicitly state the number of subjects who exceeded the 90% accuracy threshold. Including these quantitative results will make the abstract more informative and allow readers to better evaluate the claimed performance advantages.
Author response: Thank you for this valuable suggestion. We agree that providing concrete quantitative results in the abstract enhances clarity and scientific completeness. These are results that fit your suggestion: (i) explicit latency values: BELT-lite achieves a mean latency of 6.75 ms per sample on ARM Cortex-A7 hardware, compared to EEGNet's 8.36 ms ($p < 10^{-17}$), a 21% speed improvement; (ii) accuracy comparison with EEGNet: this speed gain comes at the cost of a modest but statistically significant accuracy reduction of approximately 2.7 percentage points compared to EEGNet; and (iii) number of high-performing subjects: after posterior fine-tuning, four subjects from Dataset B and two from Dataset A exceeded 90% accuracy.
Author action: Following your recommendation, we have revised the abstract to include mentioned results.

Reviewer #2, Concern #2: The Introduction is overly long and contains an extensive amount of historical background that does not directly support the motivation of the present study. Much of the section reads as a narrative review rather than a focused explanation of the research gap and the specific contributions of the BELT architecture and BELT-lite model. The authors should shorten, reorganize, and streamline the Introduction so that it more clearly highlights the current limitations in BCI research and how the proposed framework addresses them within the BELT paradigm.
Author response: Thank you for this insightful observation. We agree that

the original Introduction was overly lengthy and included historical background that did not directly support the study's motivation. Following your recommendation, we have substantially shortened, reorganized, and streamlined the Introduction to focus specifically on: (i) the advantages of deep learning for handling EEG signal variability in BCI systems; (ii) why Edge–Cloud architectures are particularly well-suited to accommodate the computational demands of deep learning in portable BCI devices; (iii) the need for a dedicated architectural framework when deploying deep learning within an Edge–Cloud paradigm for BCI; and (iv) how the proposed BELT architecture and BELT-lite model directly address these requirements.

Author action: The revised Introduction now is shorter and follows the narrative of the mentioned 4 steps. Irrelevant historical material and tangential discussions have been removed to improve readability and impact.

Reviewer #2, Concern #3: Although the manuscript provides mathematical descriptions of the architectural components, it does not offer sufficient detail about how these components were implemented in practice. The manuscript does not specify what software packages, libraries, or custom scripts were used. Without this information, readers will find it difficult to reproduce the results. Could the authors include more implementation details and, if possible, provide publicly accessible code.

Author response: Thank you for this important observation regarding reproducibility. We agree that providing sufficient implementation details and accessible code is essential.

Author action: To address this, we have: (i) made the complete source code for the BELT framework and BELT-lite model publicly available at:

https://github.com/adanayi/BELT

with well-documented Jupyter notebooks covering data preprocessing, model training, evaluation procedures, and compression experiments and sharing them publicly on the linked repository; (ii) expanded the "Implementation" subsections to include additional practical details. The repository will be maintained and updated to address any questions from the community.

Reviewer #2, Concern #4: The descriptions of the EEG dataset, segmentation steps, preprocessing procedures, filtering operations, and validation strategy currently appear in the Results section rather than the Methods section, which makes the manuscript difficult to follow. These methodological details are essential components of the experimental workflow and should be presented in the Methods section so that readers can fully understand and replicate the analyses before interpreting the results.

Author response: Thank you for this important observation. You are absolutely correct—methodological details belong in the Methods section, not in Results.

Author action: Following your suggestion and in alignment with Comments 31 and 47, we have moved all experimental procedure descriptions to the Materials and Methods section.

Reviewer #2, Concern #5: Several figures appear out of order and lack proper labeling, making it challenging to understand their relationship to the text. For instance, Figures 1, 2, and 3 do not display clear figure labels or captions on the page, and the sequence of figures appears reversed. The authors should revise the figure order, ensure that each figure includes a complete and informative caption, and verify that all figures are correctly referenced in the manuscript.

Author response: Thank you for this important observation regarding figure presentation. Regarding table and figure placement: we use the LaTeX [!ht] and [!h] placement specifiers for tables and figs (respectively) to position floats as close as possible to their first citation, and have verified that each table appears on the same page as or immediately after its first reference. We would appreciate any suggestion on this.

Author action: We have carefully reviewed all figures and implemented the following improvements: (i) complete captions: every figure now includes a detailed, self-contained caption that clearly describes what is shown, defines all visual elements (bars, error bars, colors), and summarizes key findings; (ii) figure order verification: we have verified that all figures are numbered sequentially in the order they appear in the text and that each figure is explicitly referenced before it appears; (iii) labeling: all axes, legends, and visual elements are clearly labeled within each figure.

Reviewer #2, Concern #6: The manuscript would benefit from clearer evaluation metrics and statistical testing, particularly regarding the use of K-fold cross-validation with nine subjects. It is not clear how the folds were constructed or how subject-level variation was handled. The authors should provide a flowchart illustrating the overall data analysis pipeline, including data preprocessing, training and validation structure, cross-validation workflow, and the specific comparisons performed for each component of the BELT framework.

Author response: Thank you for this excellent suggestion regarding evaluation clarity. We agree that transparent reporting of the cross-validation methodology and data analysis pipeline is essential for reproducibility.

Author action: To address this, we have made the following additions and clarifications: (i) created a comprehensive flowchart (Fig 4) illustrating the entire experimental workflow, including dataset combination, segmentation rules, subject mapping, cross-validation strategy, evaluation scenarios, posterior finetuning, and statistical testing methodology and addition of new subsection "Statistical tests methodology" explaining this title; (ii) explicitly stated that for each of the 10 folds, the entire dataset is shuffled with a fixed random seed and

split into 90% training and 10% validation subsets; (iii) clarified that all reported accuracies are computed per subject across the 10 folds and presented as mean ± standard deviation, with statistical tests applied to subject-level means; (iv) documented the complete segmentation process in the `segmentation.ipynb` notebook in the GitHub repository; and (v) explicitly stated which comparisons correspond to which BELT component with clear references to tables and figures.

Reviewer #2, Concern #7: The authors should provide a clear link to the public EEG database used in the study so that readers can access the dataset easily. Additionally, if feasible, the authors may share the data processing and analysis scripts used in the study because the procedure is extremely complex. Availability of code and data would significantly enhance the reproducibility and overall value of this work to the research community.

Author response: Thank you for this important suggestion regarding data and code availability. We fully agree that making both data access and processing scripts publicly available is essential for reproducibility.

Author action: We have added direct links to the official download pages for both BCI Competition IV-2a and IV-2b datasets on the homepage of our GitHub repository (*https://github.com/adanayi/BELT*); (ii) provided well-documented Jupyter notebooks covering every stage of the experimental pipeline, including the range from `segmentation.ipynb` for preprocessing to statistical analysis of each experiment.

We also note that we have fixed all random seeds in the code to make everything reproducible.
