# Supplementary material for: Improved motor imagery BCI performance via task-unaware compression in the BELT Bayesian Edge-Cloud architecture
Source: PLoS One. 2026 Aug 21;21(8):e0354976. doi: 10.1371/journal.pone.0354976 (PMC13497277; doi:10.1371/journal.pone.0354976)
Supplement: Supplementary file 2 [file pone.0354976.s003.pdf]

Response to Reviewers

Improved Motor Imagery BCI Performance via Task-Unaware Compression in the BELT Bayesian Edge-Cloud Architecture

PLOS ONE

June 1, 2026

Dear Editor,

We are grateful for the opportunity to revise our manuscript and for the thorough, constructive comments from both reviewers. The feedback has led to substantial improvements in the rigour, clarity, and focus of the paper. In particular, we have corrected a methodological flaw (the oracle selection), restructured and tightened the introduction, clarified the cross-validation strategy, and updated all statistical tests accordingly.

We are uploading the following files:

1. A point-by-point response to all reviewer comments (below)
2. A marked-up manuscript showing all changes, generated using `latexdiff` ("Revised Manuscript with Track Changes")
3. A clean revised manuscript without highlighting ("Manuscript")
4. Updated figures

We trust that the revised manuscript now meets the expectations of PLOS ONE.

Sincerely,
Hamid Soltanian-Zadeh
(on behalf of all authors)

Reviewer 1

Reviewer #1, Concern #1: At Section Results, subsections Prior Detector and Posterior Detector: Regarding the significant result for oracle selection, both for prior and posterior detectors, the first issue to highlight is that, for what I have searched, oracle selection is a methodology created by the authors. They don't cite any reference to this methodology being used elsewhere. The second issue is that this methodology is guaranteed to always be better than the other augmentation levels, since it picks the best results for all augmentation levels. This likely leads to over-fitting. Only use oracle selection if the authors can present convincing evidence from other studies that this methodology is

appropriate.

Author response: We thank the Reviewer for this critical comment, which prompted us to re-examine the computation of the a^* column. We discovered that the "oracle" had been selected using the test set rather than a held-out validation set, constituting an invalid upper bound and over-fitting. We have therefore **removed the a^* column from all experiments** and re-ran the statistical analyses using only the fixed augmentation levels. All results sections, tables, figures, and captions have been revised accordingly. The corrected statistics are now:

- **Prior detector:** Friedman $\chi^2(4) = 12.04$, $p = 0.017$; Nemenyi post-hoc: $a_{aug} = 3$ significantly better than baseline $a_{aug} = 0$ ($p = 0.019$), $a_{aug} = 1$ borderline ($p = 0.048$); all other comparisons non-significant.
- **Posterior detector:** Two conditions $a_{aug} = 0$ and $a_{aug} = 1$; Wilcoxon test showed significant improvement with augmentation ($W = 30.0$, $p = 0.0139$; mean 84.3% vs. 82.6%). The combined prior–posterior comparison (four conditions) was updated: Friedman $\chi^2(3) = 47.27$, $p = 3.05 \times 10^{-10}$; Nemenyi shows both posterior conditions outperform both prior conditions, while the small augmentation effect within posteriors is not significant after correction.
- **Frozen LTI:** Wilcoxon test significant ($W = 7.0$, $p = 1.45 \times 10^{-4}$); means 87.2% vs. 86.4% (Dataset B) and 78.8% vs. 75.5% (Dataset A).
- **Classifier-only:** Wilcoxon test significant ($W = 7.0$, $p = 1.45 \times 10^{-4}$); means 86.3% vs. 85.9% (Dataset B) and 76.2% vs. 74.5% (Dataset A).
- **Summary of fine-tuning depth:** New subsection using $a_{aug} = 1$; Friedman $\chi^2(2) = 22.11$, $p = 1.58 \times 10^{-5}$; Nemenyi: full > classifier-only ($p = 9.1 \times 10^{-6}$), frozen LTI not different from full ($p = 0.16$) but better than classifier-only ($p = 0.013$).
- **Conclusion gain values:** Updated from the invalid oracle gains to those obtained with $a_{aug} = 1$: Dataset B prior 77.2% → 87.9% (+10.7 pp); Dataset A 56.7% → 80.6% (+23.9 pp).

We appreciate the Reviewer's vigilance, which allowed us to correct this error and present a transparent analysis.

Author action: Removed the a^* column from all tables and figures; re-ran and updated all relevant statistical tests (Friedman, Nemenyi, Wilcoxon); revised all associated text, captions, and the Conclusion. The affected sections are:

- Prior detector: lines 584–605, Table 3.
- Posterior detector: lines 632–642, Table 4; lines 649–686, Figs. 4–6.
- Frozen LTI: lines 712–715, Table 5.
- Classifier-only: lines 721–724, Table 6.

- Summary of Fine-Tuning Depth (new subsection): lines 726–744, Figs. 7–8.
- Conclusion: lines 893–895.

.

Reviewer #1, Concern #2: These sections should be bigger to include more details on the relevance of the faster runtime. This is the major advantage of the architecture and should be spotlighted. What are the benefits of a faster BCI? EEGNet was not fast enough? Does this impact possible sampling rates of the BCI? Does this impact the amount of time between recording the EEG of the subject, training, and recording again on a model trained for this subject? How many samples were used in the runtime evaluations? All these points would contribute to highlight the impact of the study.

Author response: We have expanded the "Embedded Suitability" subsection and the Conclusion to detail the practical benefits of faster inference. Specifically, we now discuss (i) lower energy consumption and battery-life extension, (ii) freed CPU resources for concurrent signal-processing tasks, (iii) reduced accumulated latency in sliding-window or multi-channel pipelines, (iv) the potential for fixed-function DSP acceleration, and (v) faster and cheaper cloud fine-tuning due to the low parameter count, which shortens the calibration loop for pay-as-you-go cloud environments. We also clarified that the runtime benchmark used 10,000 total inferences and explicitly noted that EEGNet's latency, while acceptable in the lab, is suboptimal on ultra-low-power hardware. These additions address all points raised without requiring new citations.

Author action: Added detailed paragraphs to "Embedded Suitability" (lines 823–876) and the Conclusion (lines 893–895); added sentence clarifying the total number of runtime inferences.

Reviewer #1, Concern #3: Include the results for task unaware at the abstract.

Author response: We have added a sentence to the Abstract explicitly reporting that the task-unaware autoencoder outperformed task-aware and task-only alternatives, providing the best accuracy-compression trade-off.

Author action: Revised the Abstract (third paragraph now begins "Compression via the task-unaware autoencoder...").

Reviewer #1, Concern #4: The current title should summarize the most relevant contributions. The title should emphasize the gain in performance. A change in the title is recommended, but the architecture name should not be changed.

Author response: We thank the Reviewer for this constructive suggestion.

We have changed the title to “Improved Motor Imagery BCI Performance via Task-Unaware Compression in the BELT Bayesian Edge-Cloud Architecture”. This title highlights the central performance-improvement result (task-unaware compression) while keeping the architecture name BELT intact.

Author action: Changed the title in the manuscript.

Reviewer #1, Concern #5: The link to the data on Google Drive requires acceptance of the authors. The data are not fully available without restriction.

Author response: We appreciate the Reviewer for identifying this oversight. The sharing settings have been changed so that the link is publicly accessible without any permission step. The data are now fully available without restriction.

Author action: Updated the sharing settings of the Google Drive link.

Reviewer #1, Concern #6: (Minor issues) Various typographical and formatting issues throughout the manuscript.

Author response: All listed minor issues have been addressed:

- Abstract: added space before the compression sentence (now revised).
- Line 77: corrected “light-wieght” to “light-weight”.
- Line 230: added missing citation for Al-Saegh et al. and corrected spelling of the author name.
- Line 247: clarified that we prefer the term “reconstructed” for $\vec{x}_r$.
- Line 299: replaced k with j as previously agreed.
- Line 471: simplified to “We fixed the random seed”.
- Lines 479–494: moved the “Statistical tests methodology” heading to the correct position.
- Line 569: the abbreviation GAN was already defined earlier (DC-GANs, lines 229–231); no further change required.
- Line 639: removed doubled plus-minus sign.
- Lines 643–654: moved methodological description to the Data Augmentation subsection in Methods.
- Line 745: corrected “accuracie” to “accuracies”.
- Figures 10 and 11: swapped x- and y-axis labels.

.

Author action: Applied all minor corrections as listed.

Reviewer #1, Concern #7: (Line 147-150) Rephrase the sentence about publicly available datasets and DL data needs.

Author response: We rephrased the sentence to state that deep learning requires large amounts of data, and that the increase in publicly available datasets helps meet this demand. All original citations are retained.

Author action: Revised the sentence as recommended.

Reviewer #1, Concern #8: (Line 394) The definition of the chosen value for k' is not given anywhere.

Author response: In our implementation the posterior objective is not instantiated with a numeric k'; instead, the balance is controlled implicitly through the training protocol: the prior model uses a learning rate of 10^{-3} on multi-subject data, while posterior fine-tuning uses a higher learning rate of 5×10^{-3} on a single subject's data. The small dataset acts as a regulariser, removing the need for an explicit k'. We have added a clarifying sentence in the Implementation section.

Author action: Added explanation of how k' is implicitly controlled (lines 619–628).

Reviewer #1, Concern #9: (Lines 813-814) The comparison of 18 unseen subjects against 9 seen values is unbalanced.

Author response: We have corrected the text to clarify that the Wilcoxon test compares the 18 subjects' prior accuracies in the unseen condition against their own prior accuracies when they were included in the full prior training (seen), drawn from Table X ($a_{aug} = 0$). This is now a proper paired comparison with $N = 18$ pairs. The test results and mean drops are unchanged. Also, we corrected a misstatement in the EEGNet accuracy comparison (line 836) where only 9 participants were mentioned; it is now stated as all 18 subjects.

Author action: Revised the descriptions at lines 754–756 and 836.

Reviewer #1, Concern #10: (Line 891, 901-902) Report accuracies in percentage and latencies in ms.

Author response: We have verified that all accuracies are reported in percent (%) and all latencies in milliseconds (ms) throughout the manuscript.

Author action: Units verified and corrected where necessary.

Reviewer 2

Reviewer #2, Concern #1: Please shorten and better focus the Introduction, particularly the "Literature Review and Current Challenges" section.

Author response: We have restructured the introductory material. The opening Introduction now presents a tight narrative that derives BELT's design rules. The former "Literature Review and Current Challenges" has been replaced by a new "Related Work and Current Challenges" section, organised around four themes (variability and adaptation, embedded deployment, edge–cloud communication, privacy) and explicitly stating how BELT addresses each challenge. The combined length has been reduced from approximately 4 1/3 pages to 3 pages, retaining all pertinent citations.

Author action: Rewrote and significantly shortened the Introduction and Related Work sections. Additionally, removing the pipeline figure (previously Fig. 1) caused all subsequent figure numbers to shift backward by one; we have verified that all cross-references now point to the correct figures.

Reviewer #2, Concern #2: Clarify the cross-validation strategy; address potential concerns regarding data leakage, particularly in the "full prior training using all data" approach, and clarify how generalization performance is ensured.

Author response: We have rewritten the Cross Validation Strategy subsection to explicitly distinguish the two evaluation schemes: (i) subject-dependent 10-fold CV (full prior training), which evaluates population fitting and is used for all method comparisons (lines 395–404), and (ii) leave-one-subject-out with repeated folds, which guarantees a leakage-free assessment of cross-subject generalization (lines 405–419). We clearly explain that validation segments are never used for training, so no leakage occurs. The term "validation accuracy" consistently refers to performance on the held-out portion.

Author action: Rewrote the Cross Validation Strategy subsection to fully detail the two CV schemes and their leakage-free nature.
