# Supplementary material for: Improved motor imagery BCI performance via task-unaware compression in the BELT Bayesian Edge-Cloud architecture
Source: PLoS One. 2026 Aug 21;21(8):e0354976. doi: 10.1371/journal.pone.0354976 (PMC13497277; doi:10.1371/journal.pone.0354976)
Supplement: Peer review history

# Peer review: Decision Letter 0

**DOI:** 10.1371/journal.pone.0354976.r001

**Authors:** Ahmed Shaheen

11 Dec 2025

PONE-D-25-53502

Bayesian Edge-Cloud BCI Architecture: Lightweight Classification and Task-Aware Compression

PLOS One

Dear Dr. Soltanian-Zadeh,

Thank you for submitting your manuscript to PLOS ONE. After careful consideration, we feel that it has merit but does not fully meet PLOS ONE’s publication criteria as it currently stands. Therefore, we invite you to submit a revised version of the manuscript that addresses the points raised during the review process.

Please submit your revised manuscript by Jan 25 2026 11:59PM. If you will need more time than this to complete your revisions, please reply to this message or contact the journal office at plosone@plos.org. When you're ready to submit your revision, log on to https://www.editorialmanager.com/pone/ and select the 'Submissions Needing Revision' folder to locate your manuscript file.

Please include the following items when submitting your revised manuscript:

- A rebuttal letter that responds to each point raised by the academic editor and reviewer(s). You should upload this letter as a separate file labeled 'Response to Reviewers'.
- A marked-up copy of your manuscript that highlights changes made to the original version. You should upload this as a separate file labeled 'Revised Manuscript with Track Changes'.
- An unmarked version of your revised paper without tracked changes. You should upload this as a separate file labeled 'Manuscript'.

If you would like to make changes to your financial disclosure, please include your updated statement in your cover letter. Guidelines for resubmitting your figure files are available below the reviewer comments at the end of this letter.

If applicable, we recommend that you deposit your laboratory protocols in protocols.io to enhance the reproducibility of your results. Protocols.io assigns your protocol its own identifier (DOI) so that it can be cited independently in the future. For instructions see: https://journals.plos.org/plosone/s/submission-guidelines#loc-laboratory-protocols. Additionally, PLOS ONE offers an option for publishing peer-reviewed Lab Protocol articles, which describe protocols hosted on protocols.io. Read more information on sharing protocols at https://plos.org/protocols?utm_medium=editorial-email&utm_source=authorletters&utm_campaign=protocols.

We look forward to receiving your revised manuscript.

Kind regards,

Ahmed Shaheen

Guest Editor

PLOS One

Journal Requirements:

When submitting your revision, we need you to address these additional requirements.

1.Please ensure that your manuscript meets PLOS ONE's style requirements, including those for file naming. The PLOS ONE style templates can be found at

https://journals.plos.org/plosone/s/file?id=wjVg/PLOSOne_formatting_sample_main_body.pdf and

https://journals.plos.org/plosone/s/file?id=ba62/PLOSOne_formatting_sample_title_authors_affiliations.pdf

2. Please note that PLOS One has specific guidelines on code sharing for submissions in which author-generated code underpins the findings in the manuscript. In these cases, we expect all author-generated code to be made available without restrictions upon publication of the work. Please review our guidelines at https://journals.plos.org/plosone/s/materials-and-software-sharing#loc-sharing-code and ensure that your code is shared in a way that follows best practice and facilitates reproducibility and reuse.

3. When completing the data availability statement of the submission form, you indicated that you will make your data available on acceptance. We strongly recommend all authors decide on a data sharing plan before acceptance, as the process can be lengthy and hold up publication timelines. Please note that, though access restrictions are acceptable now, your entire data will need to be made freely accessible if your manuscript is accepted for publication. This policy applies to all data except where public deposition would breach compliance with the protocol approved by your research ethics board. If you are unable to adhere to our open data policy, please kindly revise your statement to explain your reasoning and we will seek the editor's input on an exemption. Please be assured that, once you have provided your new statement, the assessment of your exemption will not hold up the peer review process.

4. Please ensure that you refer to Figure 4 in your text as, if accepted, production will need this reference to link the reader to the figure.

5. If the reviewer comments include a recommendation to cite specific previously published works, please review and evaluate these publications to determine whether they are relevant and should be cited. There is no requirement to cite these works unless the editor has indicated otherwise.

[Note: HTML markup is below. Please do not edit.]

Reviewers' comments:

Reviewer's Responses to Questions

**Comments to the Author**

1. Is the manuscript technically sound, and do the data support the conclusions?

The manuscript must describe a technically sound piece of scientific research with data that supports the conclusions. Experiments must have been conducted rigorously, with appropriate controls, replication, and sample sizes. The conclusions must be drawn appropriately based on the data presented.

Reviewer #1: Partly

Reviewer #2: Yes

**********

2. Has the statistical analysis been performed appropriately and rigorously?

Reviewer #1: No

Reviewer #2: No

**********

3. Have the authors made all data underlying the findings in their manuscript fully available?

The PLOS Data policy requires authors to make all data underlying the findings described in their manuscript fully available without restriction, with rare exception (please refer to the Data Availability Statement in the manuscript PDF file). The data should be provided as part of the manuscript or its supporting information, or deposited to a public repository. For example, in addition to summary statistics, the data points behind means, medians and variance measures should be available. If there are restrictions on publicly sharing data—e.g. participant privacy or use of data from a third party—those must be specified.

Reviewer #1: No

Reviewer #2: No

**********

4. Is the manuscript presented in an intelligible fashion and written in standard English?

PLOS ONE does not copyedit accepted manuscripts, so the language in submitted articles must be clear, correct, and unambiguous. Any typographical or grammatical errors should be corrected at revision, so please note any specific errors here.

Reviewer #1: Yes

Reviewer #2: Yes

**********

5. Review Comments to the Author

Please use the space provided to explain your answers to the questions above. You may also include additional comments for the author, including concerns about dual publication, research ethics, or publication ethics. (Please upload your review as an attachment if it exceeds 20,000 characters)

Reviewer #1: Review questions:

1. Is the manuscript technically sound, and do the data support the conclusions?

Partly. The authors did few statistical tests, relying more on descriptive statistics. The conclusions would be more rigorously supported with statistical tests.

2. Has the statistical analysis been performed appropriately and rigorously?

No. The suggestions about statistical test are described on the comments.

3. Have the authors made all data underlying the findings in their manuscript fully available?

No. The authors say that All code underlying the findings is available at: https://github.com/adanayi/BELT, in the "research" folder. The repository will be made public upon acceptance, but until now I or the public have no access to it.

4. Is the manuscript presented in an intelligible fashion and written in standard English?

Yes. The language is clear, without typographical or grammatical errors.

Overall comments:

The focus of the study is the proposal of BELT (Bayesian Edge-Cloud architecture with Lightweight classification and Task-aware compression), a BCI architecture, and BELT-lite, a model of this architecture focused on embedded systems.

The authors compared the proposed system with EEGNet. They achieved similar accuracy and faster performance, which are both the main contributions of BELT-lite. A common strategy to achieve smaller processing times is to sacrifice accuracy, but this doesn’t seem to be the case.

To achieve similar accuracy the authors relied on an approach with a basic similarity with EEGNet: both are based on deep learning (DL). In order to do DL, the authors deal with the common issues of DL, like the demand for a lot of training data, the associated computational cost and the issue of portability of putting all the necessary processing units into a portable device.

However, along the introduction the authors talk about many of these problems as being fundamentally related to the area of BCI. However, BCI approaches that don’t use DL or any type of artificial intelligence, will encounter different issues. The authors should make this clear at the introduction. Also, some of the mentioned problems of BCIs can be questioned of actually existing, for instance: BCIs are not cost-effective, mass-producible, remain scarce, have re-implementation issues, have interdisciplinary issues and are not practical. Some other aspects of BCIs are actually problematic, but the approach of this study is not

dealing with those, specifically the issues of non-stationarity and portability. I present these aspects in detail, while pointing to the parts of the text where they appear.

To optimize the system the authors tested the effects of many parameters. However, they mostly do this by comparing the accuracies of the average of all subjects or of single subjects. This is not a consistent approach. Significant p-values would tell if a difference in accuracy is consistent across subjects. That should be the goal: to find parameter values that are better to almost everyone. That brings interpretability to the results. For all parameters choices that are user-specific but don't follow a rule known to humans, this means that only the computer will know what is being done. The authors should obtain significant p-values, as this brings to humans the information about how something happens. Science is about bringing knowledge to humans, not to computers.

While evaluating the p-values the authors may deal with another issue of the study, that is the small sample size of the dataset. By using more datasets, the authors may increase the sample size. My suggestion is to include the Graz data set A, instead of using only the data set B.

Overall the study has potential, but the decision to accept or to reject must be based on the results obtained by evaluating p-values, after the authors modify the manuscript based on the suggestions of this review.

Detailed comments:

Comment 1. Line 9: The authors claim that BCI systems remain neither cost-effective nor mass-producible. However, this is not the case, since there are many consumer-grade BCI systems. This review mentions 5 of these systems:

Sabio, J., Williams, N. S., McArthur, G. M., & Badcock, N. A. (2024). A scoping review on the use of consumer-grade EEG devices for research. PLoS One, 19(3), e0291186.

The authors should rethink the line of argumentation.

Comment 2. Lines 18-22: The authors claim that consumer-grade BCI tools remain scarce and point to some barriers for that, specifically the biological variability, ethical and privacy issues, and the technical constraints of real-time portable implementations. As I already mentioned, there are at least 5 consumer-grade BCI systems. If this is too little or too much is a matter of personal opinion. However, I don’t see that the presented barriers are what actually defines the number of available options. This seems more related to economical dynamics of a niche market. The market of mobile operating systems, for instance, is represented by a duopoly formed by Android and iOS systems, but this is much more related to economy than to science. A similar situation occurs in BCI systems. As showed in the review I mentioned (Sabio, 2024), Emotiv leads the market by a wide gap. According to the homepage of their website (https://www.emotiv.com/), they sold more than 45 thousand devices. People tend to buy something that has already been proven to work, which makes it harder for new devices to join the market. Also, this review shows that EPOC usage is wide-spread around the world:

Williams, N. S., McArthur, G. M., & Badcock, N. A. (2020). 10 years of EPOC: A scoping review of Emotiv’s portable EEG device. BioRxiv, 2020-07.

Based on that, the authors should avoid stating that BCI tools remain scarce, and that this scarcity is caused by the mentioned barriers. This line of reasoning is not necessary for the goals of the study.

Comment 3. Line 33: The authors mention a re-implementation issue. Actually this is an issue of lack of code availability from other studies. Reference 7 re-implements the code for a technique because the work they are using did not share the code to implement the technique. It’s not because of difficulty to generalize the solutions. Rewrite this part.

Comment 4. Line 34: The authors state that the re-implementation issue is described as a need for interdisciplinary teams. I think the authors mean that the need of re-implementation demands interdisciplinary teams. But this was already said. Explain better.

Comment 5. Line 59: Removing the word “still”, the phrase would make much more sense.

Comment 6. Line 60: When talking about non-stationarity, the authors should avoid mentioning the reference 13, which doesn’t use this term. They use the term day-to-day variability. Instead the authors should cite reference 5, which mentions stationary and non-stationary signals in BCI Challenges. Actually, this is the only reference that I found, among the references cited in sentences in the text that talk about stationarity, that actually uses the word stationary when talking about signals. I think stationary usually has a different meaning in the context of signal analysis, that is specific to characteristics of a single time series that are constant along time, and not as a generic term for variability.

Comment 7. Lines 61-62: This phrase is very confusing. I don’t see why this decoding, that can be made with any device, requires substantial processing power. I think the authors want to say that portability is necessary, but so as is processing power. This can be said, but without the word consequently, since I don’t see the relation between portability/processing power with user-adaptation/non-stationarity.

Comment 8. Line 64: Again, I recommend avoid the use of the term non-stationarity as a generic term to mean variability. Stationary is usually evaluated along a single time series. It’s uncommon to use this term when talking about the day-to-day variability. Special attention should be made for reference 15, where they do not use the term stationarity. Instead they talk about inter-subject variability. This source of variability is even less associated with stationarity, since it has no relation with time, so the term non-stationarity is clearly misused here. It’s best to avoid the use of the term non-stationarity throughout the paper, and use terms related to variability, and to the type of variability accordingly.

Comment 9. Line 72: The authors should mention here, why Edge-Cloud architectures are more viable than cloud-based systems. I see they start to define Edge-Cloud at line 200, but a brief explanation here could help the understanding of the readers.

Comment 10. Line 75: As I mentioned while commenting lines 9 and 18, the argument that BCI systems are not practical needs to be revisited. BCI systems are practical and are being

used world-wide. If the authors don’t make clear the advantage of Edge-Cloud architecture, the readers will have difficulties to understand the problem, especially when they do not agree with mentioned impracticability. So far in the reading, I don’t see the advantage of the proposed architecture. This can make the reader stop the reading at this point.

Comment 11. Line 95: This brings the idea that BELT-lite is practical and BELT is impractical. And the whole point so far it to make things practical. So, BELT is not the desired framework, but BELT-lite. If that’s not the case, rewrite this part.

Comment 12. Line 96: Actually, the arguments presented were about deep learning. I understand that deep learning is a subfield of neural networks, but you can’t discuss something specific and then say, without a reason, that you are going to use something more generic, based on the presented arguments. I see that your model is actually based on deep learning, so it’s better to keep consistency and say that BELT-lite model is built using deep learning.

Comment 13. Line 101: So far in the reading it’s still not clear to me which are the interdisciplinary problems and how they can be solved by architecture adaptation.

Comment 14. Line 112: Here I finally see the advantages of Edge-Cloud. This should happen earlier.

Comment 15. Line 122: This contradicts the idea that BCIs are not practical.

Comment 16. Line 125: Here you explicitly say that BCIs are practical.

Comment 17. Line 129: I disagree with the comment “This definition is more emphasizing the communicational side of BCI rather than the direct usage or application of a BCI system”. The usage or application of a BCI system, inevitably requires a communication between the brain and an external device. Different applications have different adherence to the term communication if you think about person to person communication. But, for being a BCI, they all enable a communication between the brain and a device, according to Vidal’s definition. For instance, both a system to send a message to somebody based on brain activity and a system that emits a sound alarm when detecting driver drowsiness are BCIs.

Comment 18. Lines 133-144: This paragraph can be heavily summarized. BCI is a subfield of HCI, but this does not bring any conclusions. EEG-based BCIs are not wearable in nature, since many of these systems are not wearable. The important point of this paragraph is the importance of portability, which is evident. Especially when talking about consumer-grade BCIs. In that case, the bigger the portability, the better.

Comment 19. Line 149: I still don’t get what the authors define as the communicational aspect of BCI. I think the authors should make it more clear.

Comment 20. Lines 154-160: The authors say that exploring portability demands large-scale data collection, but I don’t see the connection. The validation of generalizability can occur with the same dataset size requirements for any validation. The authors also say that can achieving portability at scale is only feasible through mass production, particularly given the

time and cost associated with acquiring EEG-BCI data in controlled research settings. However, this is not the case. The same can happen by conducting many data collections with the production of a few portable BCI units, in a multi-centric study. The authors should rewrite these parts, talking about the importance of portability, large-scale data collection and mass-production, but without stating that these things are always co-dependent. At line 169 the authors say that DL requires large amounts of training data. This makes more sense. So the authors should say the requirements for a lot of data is caused by dealing with DL, rather than caused by portability.

Comment 21. Line 193: This adaptation is not necessarily performed on the same device, as the authors suggest on the next paragraph a solution that involves it not being performed on the same device. Rewrite.

Comment 22. Equation 1: For better understanding of the equation, the authors should denote what is N.

Comment 23. Line 242: Cite some literature mentioning this improvement, as well as listing some techniques for data augmentation with EEG data.

Comment 24. Equation 3: From my understanding, probability notation is always between parentheses, not brackets. Let me know if I’m wrong.

Comment 25. Equation 4: θc, θd and c with an arrow on top are not defined.

Comment 26. Equations 5 and 6 are not mentioned in the text. The text doesn’t make clear how Lr and Lt are calculated. These equations imply that the calculations involve a Xr, but this variable is defined only in line 274, when talking about step 6. Perhaps these equations should be mover to be near the paragraph talking about step 6. All these problems bring difficulty to understand the equations.

Comment 27. Line 276: Combing this paragraph with the previous one would make it more clear that this line is talking about equation 7.

Comment 28. Line 317: The citation and the position of Table 1 should be moved to the beginning of the Architecture section, just before the first usage of mathematical notations. This can help readers understand the equations. For readers unfamiliar with probabilistic mathematical notation, I recommend to add Pr (A | B), described as the probability of event A given event B.

Comment 29. Line 366: This is the first time you are talking about DSP, but this abbreviation is not defined anywhere. I assume this is Digital Signal Processing. The authors should define it here.

Comment 30. Lines 413-416: This paragraph confuses the reader to understand which interpretation is being talked about. Also, what promotes the consistency with the rest of the detector?

Comment 31. Lines 418-557: All these parts are detailing how everything was executed, so should be moved to the Material and Methods section.

Comment 32. Line 435: How artifacts and acquisition errors were detected? All the details about this preprocessing should be mentioned, as applied filters, for example.

Comment 33. Line 447: Put the link for the repository in the text.

Comment 34. Line 484: It’s better to rewrite this, avoiding narrative citations, that is, when the citation is part of the text.

Comment 35. Line 499: Cite the findings

Comment 36. Line 542: The parameter k_aug can easily be confused with k. Use a different nomenclature for parameters. I recommend using the letter a instead of k_aug, to indicate the level of augmentation.

Comment 37. 544: Which augmentation techniques were used?

Comment 38. Tables 3 - 7: The authors are confusing k and k_aug. The legend says k_aug but the column headers say k.

Comment 39. Line 564: I don’t see what is the value of k, that is, the number of folds. How many models were used for each subject in each fold? From my understanding, the model is only one: BELT-lite. So I don’t understand how to pick the best-performing models.

Comment 40. Lines 601-603: The authors should avoid to draw conclusions based on differences of mean accuracy, as they can be caused by chance. To know if a difference in accuracy is big enough to be considered the authors should apply some repeated measures statistical testing, like the Friedman test, for each table, to see if k_aug influence is significant. If it is, the authors should apply post-hoc tests, to find out which pairs of k_aug are significantly different. For examples, the authors should check the following studies, which were cited by reference 33:

Bang, J. S., Lee, M. H., Fazli, S., Guan, C., & Lee, S. W. (2021). Spatio-spectral feature representation for motor imagery classification using convolutional neural networks. IEEE Transactions on Neural Networks and Learning Systems, 33(7), 3038-3049.

Dai, G., Zhou, J., Huang, J., & Wang, N. (2020). HS-CNN: a CNN with hybrid convolution scale for EEG motor imagery classification. Journal of neural engineering, 17(1), 016025.

When doing that, the authors may find troubles regarding the small sample size of the study. The authors should increase the number of used datasets. One easy way to do that, is to include the Graz data set A, instead of using only the data set B. Here is the reference for dataset A:

Brunner C, Leeb R, Müller-Putz G, Schlögl A, Pfurtscheller G (2008) BCI Competition 2008–Graz data set A. Inst Knowl Discov Graz Univ Technol 16:1–6

Comment 41. Fig 4: I ask for an additional figure where each bar represents a k_arg level, and the error bars represent the standard error from mean across subjects. This would be a good way to show differences promoted by k_arg.

Comment 42. Lines 617-623: If comparing the mean accuracy is not enough information to make conclusions, a worse situation is to compare the means of single subjects. I reinforce the need to perform statistical inference.

Comment 43. Line 655: No. The limited size makes the insight less significant. That happened only in two of the nine subjects, so could be happening by chance.

Comment 44. Fig 5: Instead of comparing with the best performing subject, p-values comparing each method is a better way to should, both what is different and what is not.

Comment 45. Line 690: Why the accuracies for subject 4 are the same on Table 8, but t It’s strange that

Comment 46. Line 692: Looking at Table 8, I don’t see that the exceptions are s3 and s4. Only s4. But this is always the case.

Comment 47. Lines 706-749: All these details should me moved to the Materials and Methods section.

Comment 48. Fig 8: Make clear at the legend that the accuracy drop is caused by compression.

Comment 49. Line 758: The authors should choose different parameters names, as k is already the parameter for the number of folds.

Comment 50. Fig 9: This figure clearly could be presented with error bars, to show the variability across folds. Look in all figures for ways to present error bars.

Comment 51. Lines 798-830: The informations regarding how the tests were performed should be moved to the Materials and Methods section.

Comment 52. Lines 806-812: Here is the single time the authors perform a statistical test. The same should be applied for all analysis.

Comment 53. Lines 813-820: Perform statistical analysis here too.

Reviewer #2: This manuscript, “Bayesian Edge-Cloud BCI Architecture: Lightweight Classification and Task-Aware Compression,” addresses a timely and important topic in the field of brain–computer interface (BCI) research, particularly with respect to portable and embedded applications. The authors propose a new architecture (BELT) and a lightweight implementation (BELT-lite), which together aim to improve scalability, efficiency, and practicality in real-world BCI systems. several major issues must be addressed before the paper can be considered suitable for publication in PLOS ONE.

1. In the abstract, the authors state that BELT-lite is faster than EEGNet and that several subjects achieve accuracy above 90%, but no concrete numerical values are provided. For clarity and scientific completeness, the authors should report the actual processing time (in milliseconds or seconds) for BELT-lite versus EEGNet, as well as explicitly state the number of subjects who exceeded the 90% accuracy threshold. Including these quantitative results will make the abstract more informative and allow readers to better evaluate the claimed performance advantages.

2. The Introduction is overly long and contains an extensive amount of historical background that does not directly support the motivation of the present study. Much of the section reads as a narrative review rather than a focused explanation of the research gap and the specific contributions of the BELT architecture and BELT-lite model. The authors should shorten, reorganize, and streamline the Introduction so that it more clearly highlights the current limitations in BCI research and how the proposed framework addresses them within the BELT paradigm.

3. Although the manuscript provides mathematical descriptions of the architectural components, it does not offer sufficient detail about how these components were implemented in practice. The manuscript does not specify what software packages, libraries, or custom scripts were used. Without this information, readers will find it difficult to reproduce the results. Could the authors include more implementation details and, if possible, provide publicly accessible code.

4. The descriptions of the EEG dataset, segmentation steps, preprocessing procedures, filtering operations, and validation strategy currently appear in the Results section rather than the Methods section, which makes the manuscript difficult to follow. These methodological details are essential components of the experimental workflow and should be presented in the Methods section so that readers can fully understand and replicate the analyses before interpreting the results.

5. Several figures appear out of order and lack proper labeling, making it challenging to understand their relationship to the text. For instance, Figures 1, 2, and 3 do not display clear figure labels or captions on the page, and the sequence of figures appears reversed. The authors should revise the figure order, ensure that each figure includes a complete and informative caption, and verify that all figures are correctly referenced in the manuscript.

6. The manuscript would benefit from clearer evaluation metrics and statistical testing, particularly regarding the use of K-fold cross-validation with nine subjects. It is not clear how the folds were constructed or how subject-level variation was handled. The authors should provide a flowchart illustrating the overall data analysis pipeline, including data preprocessing, training and validation structure, cross-validation workflow, and the specific comparisons performed for each component of the BELT framework.

7. The authors should provide a clear link to the public EEG database used in the study so that readers can access the dataset easily. Additionally, if feasible, the authors may share the data processing and analysis scripts used in the study because the procedure is extremely complex. Availability of code and data would significantly enhance the reproducibility and overall value of this work to the research community.

**********

6. PLOS authors have the option to publish the peer review history of their article (what does this mean?). If published, this will include your full peer review and any attached files.

If you choose “no”, your identity will remain anonymous but your review may still be made public.

**Do you want your identity to be public for this peer review?**  For information about this choice, including consent withdrawal, please see our Privacy Policy.

Reviewer #1: **Yes:** Daniel Soares Brandão

Reviewer #2: No

**********

[NOTE: If reviewer comments were submitted as an attachment file, they will be attached to this email and accessible via the submission site. Please log into your account, locate the manuscript record, and check for the action link "View Attachments". If this link does not appear, there are no attachment files.]

To ensure your figures meet our technical requirements, please review our figure guidelines: https://journals.plos.org/plosone/s/figures

You may also use PLOS’s free figure tool, NAAS, to help you prepare publication quality figures: https://journals.plos.org/plosone/s/figures#loc-tools-for-figure-preparation.

NAAS will assess whether your figures meet our technical requirements by comparing each figure against our figure specifications.

# Author comment: Author response to Decision Letter 1

**DOI:** 10.1371/journal.pone.0354976.r002

26 Feb 2026

We thank the reviewers for their helpful comments. We have revised the manuscript based on the comments and provided our point-by-point responses.

Attachment Submitted filename: Danayi.PLOS.ONE.Response To Reviewers.pdf

# Peer review: Decision Letter 1

**DOI:** 10.1371/journal.pone.0354976.r003

**Authors:** Onder Aydemir

21 Apr 2026

PONE-D-25-53502R1

Bayesian Edge-Cloud BCI Architecture: Lightweight Classification and Task-aware compression

PLOS One

Dear Dr. Soltanian-Zadeh,

Thank you for submitting your manuscript to PLOS ONE. After careful consideration, we feel that it has merit but does not fully meet PLOS ONE’s publication criteria as it currently stands. Therefore, we invite you to submit a revised version of the manuscript that addresses the points raised during the review process.

Please submit your revised manuscript by Jun 05 2026 11:59PM. If you will need more time than this to complete your revisions, please reply to this message or contact the journal office at plosone@plos.org. When you're ready to submit your revision, log on to https://www.editorialmanager.com/pone/ and select the 'Submissions Needing Revision' folder to locate your manuscript file.

Please include the following items when submitting your revised manuscript:

- A letter that responds to each point raised by the academic editor and reviewer(s). You should upload this letter as a separate file labeled 'Response to Reviewers'.
- A marked-up copy of your manuscript that highlights changes made to the original version. You should upload this as a separate file labeled 'Revised Manuscript with Track Changes'.
- An unmarked version of your revised paper without tracked changes. You should upload this as a separate file labeled 'Manuscript'.

If you would like to make changes to your financial disclosure, please include your updated statement in your cover letter. Guidelines for resubmitting your figure files are available below the reviewer comments at the end of this letter.

If applicable, we recommend that you deposit your laboratory protocols in protocols.io to enhance the reproducibility of your results. Protocols.io assigns your protocol its own identifier (DOI) so that it can be cited independently in the future. For instructions see: https://journals.plos.org/plosone/s/submission-guidelines#loc-laboratory-protocols. Additionally, PLOS ONE offers an option for publishing peer-reviewed Lab Protocol articles, which describe protocols hosted on protocols.io. Read more information on sharing protocols at https://plos.org/protocols?utm_medium=editorial-email&utm_source=authorletters&utm_campaign=protocols.

As the corresponding author, your ORCID iD is verified in the submission system and will appear in the published article. PLOS supports the use of ORCID, and we encourage all coauthors to register for an ORCID iD and use it as well. Please encourage your coauthors to verify their ORCID iD within the submission system before final acceptance, as unverified ORCID iDs will not appear in the published article. *Only*  the individual author can complete the verification step; PLOS staff *cannot*  verify ORCID iDs on behalf of authors.

We look forward to receiving your revised manuscript.

Kind regards,

Onder Aydemir

Academic Editor

PLOS One

Journal Requirements:

If the reviewer comments include a recommendation to cite specific previously published works, please review and evaluate these publications to determine whether they are relevant and should be cited. There is no requirement to cite these works unless the editor has indicated otherwise.

Please review your reference list to ensure that it is complete and correct. If you have cited papers that have been retracted, please include the rationale for doing so in the manuscript text, or remove these references and replace them with relevant current references. Any changes to the reference list should be mentioned in the rebuttal letter that accompanies your revised manuscript. If you need to cite a retracted article, indicate the article’s retracted status in the References list and also include a citation and full reference for the retraction notice.

Additional Editor Comments:

Dear Authors,

Thank you for submitting your manuscript and for your efforts in revising it. After evaluation by the reviewers, I am pleased to inform you that your manuscript requires minor revision before it can be considered for publication. Both reviewers find the study relevant and generally well-structured. However, several points—mainly related to clarity, presentation, and some methodological aspects—should be addressed to improve the quality and transparency of the manuscript. Please carefully consider and respond to the following comments:

Reviewer 1 Comments

Please address minor typographical and formatting issues (e.g., spacing in the abstract, correction of “light-weight,” “accuracies,” and consistency in notation such as k/j).

Clarify ambiguous statements (e.g., Line 247 and the definition of k′).

Improve clarity in the discussion of publicly available datasets (Lines 147–150).

Reconsider or better justify the use of the “oracle selection” methodology. As noted, this approach may introduce bias and potential overfitting unless supported by prior literature.

Clarify the comparison between seen and unseen subjects and ensure consistency in sample sizes.

Ensure consistent reporting of units (accuracies in %, latencies in ms).

Expand the discussion on the importance and implications of faster runtime, particularly in BCI applications.

Ensure that the data availability statement complies with journal requirements (i.e., data must be fully accessible without restrictions).

Correct figure labeling issues (Figures 10 and 11).

Reviewer 2 Comments

Please shorten and better focus the Introduction, particularly the “Literature Review and Current Challenges” section.

Clarify the cross-validation strategy. The current description (10-fold vs. leave-one-subject-out) is inconsistent and should be explicitly explained.

Address potential concerns regarding data leakage, particularly in the “full prior training using all data” approach, and clarify how generalization performance is ensured.

Please revise your manuscript accordingly and provide a detailed, point-by-point response to all reviewer comments.

We look forward to receiving your revised manuscript.

Kind regards,

Önder Aydemir

[Note: HTML markup is below. Please do not edit.]

Reviewers' comments:

Reviewer's Responses to Questions

**Comments to the Author**

1. If the authors have adequately addressed your comments raised in a previous round of review and you feel that this manuscript is now acceptable for publication, you may indicate that here to bypass the “Comments to the Author” section, enter your conflict of interest statement in the “Confidential to Editor” section, and submit your "Accept" recommendation.

Reviewer #1: (No Response)

Reviewer #2: (No Response)

**********

2. Is the manuscript technically sound, and do the data support the conclusions?

The manuscript must describe a technically sound piece of scientific research with data that supports the conclusions. Experiments must have been conducted rigorously, with appropriate controls, replication, and sample sizes. The conclusions must be drawn appropriately based on the data presented.

Reviewer #1: Yes

Reviewer #2: Yes

**********

3. Has the statistical analysis been performed appropriately and rigorously?

Reviewer #1: Yes

Reviewer #2: No

**********

4. Have the authors made all data underlying the findings in their manuscript fully available?

The PLOS Data policy requires authors to make all data underlying the findings described in their manuscript fully available without restriction, with rare exception (please refer to the Data Availability Statement in the manuscript PDF file). The data should be provided as part of the manuscript or its supporting information, or deposited to a public repository. For example, in addition to summary statistics, the data points behind means, medians and variance measures should be available. If there are restrictions on publicly sharing data—e.g. participant privacy or use of data from a third party—those must be specified.

Reviewer #1: No

Reviewer #2: Yes

**********

5. Is the manuscript presented in an intelligible fashion and written in standard English?

PLOS ONE does not copyedit accepted manuscripts, so the language in submitted articles must be clear, correct, and unambiguous. Any typographical or grammatical errors should be corrected at revision, so please note any specific errors here.

Reviewer #1: Yes

Reviewer #2: Yes

**********

6. Review Comments to the Author

Please use the space provided to explain your answers to the questions above. You may also include additional comments for the author, including concerns about dual publication, research ethics, or publication ethics. (Please upload your review as an attachment if it exceeds 20,000 characters)

Reviewer #1: - Concern # 1 (Minor issues):

At the Abstract:

Add a space before the sentence starting with "Compression reduced data size".

Line 77:

Replace light-wieght with light-weight

Line 247: It's not clear what this preference means. Is it for using Xr instead of Xd? Make it clearer.

Line 299:

As the authors mentioned in the response to my Concern #36, they replaced k with j. However, this was not done on this line.

Line 745:

Replace accuracie with accuracies

On Figures 10 and 11:

The x and y axis labels are swapped.

- Concern # 2 (Is there enough public data?):

Line 147-150: I recommend rephrasing the sentence. What are the issues with the publicly available datasets? If there are no pointed issues, the fact that DL needs a lot of data is not a problem. Just say that DL needs a lot of data, and that this has been solved by the increase in publicly available datasets.

- Concern # 3 (The definition of k′):

Line 394:

I don't see anywhere the definition of the chosen value for k′.

- Concern # 4 (Oracle selection):

At Section Results, subsections Prior Detector and Posterior Detector:

Regarding the significant result for oracle selection, both for prior and posterior detectors, the first issue to highlight is that, for what I have searched, oracle selection is a methodology created by the authors. They don't cite any reference to this methodology being used elsewhere. The second issue is that this methodology is guaranteed to always be better than the other augmentation levels, since it picks the best results for all augmentation levels. This likely leads to over-fitting. Only use oracle selection if the authors can present convincing evidence from other studies that this methodology is appropriate.

- Concern # 5 (Number of seen and unseen subjects):

Lines 813-814:

Why compare 18 unseen with 9 seen? Is it possible to use 18 on both? If not, the N is 9, and not 18.

- Concern # 6 (Units of accuracies and latencies):

Line 891:

As done on the rest of the manuscript, report accuracies in percentage.

Lines 901-902:

Report latencies in ms, for being a more conventional unit of time.

- Concern # 7 (Highlight the relevance of faster runtime):

At the "Comparative Baseline Model: EEGNet", "Embedded suitability", and "Conclusion" sections:

These sections should be bigger to include more details on the relevance of the faster runtime. This is the major advantage of the architecture and should be spotlighted. What are the benefits of a faster BCI? EEGNet was not fast enough? Does this impact possible sampling rates of the BCI? Does this impact the amount of time between recording the EEG of the subject, training, and recording again on a model trained for this subject? How many samples were used in the runtime evaluations? All these points would contribute to highlight the impact of the study.

- Concern # 8 (Data availability):

The manuscript is submitted stating that all data are fully available without restriction. The code is, in fact, available on GitHub, where there is a link to the data on Google Drive. However, this link requires acceptance of the authors. So, right now, the data is not fully available without restriction, which is a requirement at Plos One.

Reviewer #2: The manuscript is generally well structured; however, several methodological and presentation issues require clarification.

First, the Introduction is comprehensive but remains overly long. In particular, the “Literature Review and Current Challenges” section includes substantial detail that appears more appropriate for the Discussion section. The Introduction would benefit from being more concise and focused on clearly motivating the study and defining the research gap.

Second, the cross-validation strategy is unclear. The manuscript refers to both “10-fold cross-validation” and a “leave-one-subject-out” approach, which are conceptually different. With 18 subjects, a leave-one-subject-out design would result in 18 folds (one subject tested per fold), not 10. It is therefore difficult to understand how the folds are defined and how many subjects are included in each test set.

In addition, the description of “full prior training using all data” raises concerns about potential data leakage. If all segments, including those from test subjects, are used during training, the evaluation may not reflect true generalization performance.

**********

7. PLOS authors have the option to publish the peer review history of their article (what does this mean?). If published, this will include your full peer review and any attached files.

If you choose “no”, your identity will remain anonymous but your review may still be made public.

**Do you want your identity to be public for this peer review?**  For information about this choice, including consent withdrawal, please see our Privacy Policy.

Reviewer #1: **Yes:** Daniel Soares Brandão

Reviewer #2: No

**********

[NOTE: If reviewer comments were submitted as an attachment file, they will be attached to this email and accessible via the submission site. Please log into your account, locate the manuscript record, and check for the action link "View Attachments". If this link does not appear, there are no attachment files.]

To ensure your figures meet our technical requirements, please review our figure guidelines: https://journals.plos.org/plosone/s/figures

You may also use PLOS’s free figure tool, NAAS, to help you prepare publication quality figures: https://journals.plos.org/plosone/s/figures#loc-tools-for-figure-preparation.

NAAS will assess whether your figures meet our technical requirements by comparing each figure against our figure specifications.

# Author comment: Author response to Decision Letter 2

**DOI:** 10.1371/journal.pone.0354976.r004

3 Jun 2026

Please see the uploaded file named Response to Reviewers. Thank you!

Attachment Submitted filename: Response To Reviewers.pdf

# Editor report: Decision Letter 2

**DOI:** 10.1371/journal.pone.0354976.r005

**Authors:** Onder Aydemir

16 Jul 2026

Improved Motor Imagery BCI Performance via Task-Unaware Compression in the BELT Bayesian Edge-Cloud Architecture

PONE-D-25-53502R2

Dear Dr. Soltanian-Zadeh,

We’re pleased to inform you that your manuscript has been judged scientifically suitable for publication and will be formally accepted for publication once it meets all outstanding technical requirements.

Within one week, you’ll receive an e-mail detailing the required amendments. When these have been addressed, you’ll receive a formal acceptance letter and your manuscript will be scheduled for publication.

An invoice will be generated when your article is formally accepted. Please note, if your institution has a publishing partnership with PLOS and your article meets the relevant criteria, all or part of your publication costs will be covered. Please make sure your user information is up-to-date by logging into Editorial Manager at Editorial Manager® and clicking the ‘Update My Information' link at the top of the page. For questions related to billing, please contact billing support.

If your institution or institutions have a press office, please notify them about your upcoming paper to help maximize its impact. If they’ll be preparing press materials, please inform our press team as soon as possible -- no later than 48 hours after receiving the formal acceptance. Your manuscript will remain under strict press embargo until 2 pm Eastern Time on the date of publication. For more information, please contact onepress@plos.org.

Kind regards,

Onder Aydemir

Academic Editor

PLOS One

Additional Editor Comments (optional):

Reviewers' comments:

# Editor report: Acceptance letter

**DOI:** 10.1371/journal.pone.0354976.r006

**Authors:** Onder Aydemir

PONE-D-25-53502R2

PLOS One

Dear Dr. Soltanian-Zadeh,

I'm pleased to inform you that your manuscript has been deemed suitable for publication in PLOS One. Congratulations! Your manuscript is now being handed over to our production team.

At this stage, our production department will prepare your paper for publication. This includes ensuring the following:

* All references, tables, and figures are properly cited

* All relevant supporting information is included in the manuscript submission,

* There are no issues that prevent the paper from being properly typeset

You will receive further instructions from the production team, including instructions on how to review your proof when it is ready. Please keep in mind that we are working through a large volume of accepted articles, so please give us a few days to review your paper and let you know the next and final steps.

Lastly, if your institution or institutions have a press office, please let them know about your upcoming paper now to help maximize its impact. If they'll be preparing press materials, please inform our press team within the next 48 hours. Your manuscript will remain under strict press embargo until 2 pm Eastern Time on the date of publication. For more information, please contact onepress@plos.org.

You will receive an invoice from PLOS for your publication fee after your manuscript has reached the completed accept phase. If you receive an email requesting payment before acceptance or for any other service, this may be a phishing scheme. Learn how to identify phishing emails and protect your accounts at https://explore.plos.org/phishing.

If we can help with anything else, please email us at customercare@plos.org.

Thank you for submitting your work to PLOS ONE and supporting open access.

Kind regards,

PLOS ONE Editorial Office Staff

on behalf of

Prof. Dr. Onder Aydemir

Academic Editor

PLOS One
